# World Congress Integrative Medicine & Health 2017: part three: Berlin, Germany. 3-5 May 2017

**DOI:** 10.1186/s12906-017-1784-2

**Published:** 2017-06-30

**Authors:** Miriam Ortiz, Katharina Schnabel, Michael Teut, Gabriele Rotter, Sylvia Binting, Margit Cree, Fabian Lotz, Ralf Suhr, Benno Brinkhaus, Mohammad M. Parvizi, Farhad Handjani, Mohammad M. Zarshenas, Mahmood R. Moein, Majid Nimrouzi, Gholamreza Hatam, Jafar Hasanzadeh, Nasrin Hamidizadeh, Mohammad M. Parvizi, Mojtaba Heydari, Mohammad R. Namazi, Zahra Parvizi, Mehdi Pasalar, Maryam Mosaffa-Jahromi, Kamran Bagheri-Lankarani, Suleiman Afsharypuor, Ali M. Tamaddon, Mohadeseh Ostovar, Giuseppe Peloni, Ingo Bolliger, Rui M. Da Cunha Faria, Pierluigi Quadri, Wilma Sanzeni, Damiano Zemp, Hilde Risvoll, Trude Giverhaug, Kjell H. Halvorsen, Marit Waaseth, Frauke Musial, Elio Rossi, Sonia Baccetti, Marco Picchi, Tommaso Conti, Fabio Firenzuoli, Carmelo Guido, Filippo Bosco, Carmelo Guido, Elio Rossi, Marialessandra Panozzo, Marco Picchi, Chiara Cervino, Linda Nurra, Elio Rossi, Marco Picchi, Fabio Firenzuoli, Antonella Traversi, Katia Vuono, Federica Sabatini, Tommaso Bellandi, Britta Rutert, Angelika Eggert, Georg Seifert, Wiebke Stritter, Christine Holmberg, Alfred Längler, Anita Salamonsen, Solveig Wiesener, Friedemann Schad, Megan Steele, Matthias Kröz, Harald Matthes, Cornelia Herbstreit, Anja Thronicke, Irene Schlingensiepen, Tido von Schoen-Angerer, Romy Schneider, Livia Waeber, Jan Vagedes, Gregor Kaczala, Cosette Pharisa, Johannes Wildhaber, Benedikt Huber, Pavel Sidorov, Evgeniya Sovershaeva, Ana P. Simões-Wüst, Anna Nietlispach, Mónica Mennet, Martin Schnelle, Ursula von Mandach, Xia Wang, Hye L. Woo, Jin M. Lee, Yuhao Wu, Yumin Cho, Younghee Yun, Hyunho Kim, Wonmo Jung, Bo-Hyung Jang, Eric Ziea, Henny Hui, Mia Li, Dora Tsui, Christine Lam, Joyce Hsieh, Edith Chan, Lynda Balneaves, Sandra Burnside, Ethel Doyle, Shelley Dorazio, Pak K. Chan, Anjali Bhagra, Po-Hsu Chen, Vincent C. H. Chung, Justin C. Y. Wu, Zhi X. Lin, Wendy Wong, Xin Y. Wu, Robin S. T. Ho, Charlene H. L. Wong, Lily Chan, Eric T. C. Ziea, William Elder, Roberto Cardarelli, Cornelia Kaspar, Robert Kempenich, Jacques Kopferschmitt, Zulj Marinko, Sebo Damir, Aleksandar Vcev, Ricardo Monezi, Eny Márcia Ruggerini, Ivna M. Fuchigami, Ana C. Moreno Mazini, Ricardo Monezi, Maria Waldenez Oliveira, Petar Papuga, Janet Schloss, Amie Steel, Márcia da Silva Jacobsen, Ricardo Monezi, Miranda Rodrigo Jacobsen, Maria T. Mangini, Gianfranco Trapani, Tiziana Di Giampietro, Luisella Zanino, Luigi Ciullo, Diego Lanaro, Francesco Cerritelli, Francesco Macrì, Andre Tsai, Chin Lin, Tu-Hsing Wu, Eduardo D’Alessandro, Sam Watts, Ying Zhang, Xufang Wu, Xun Li, Yutong Fei, Jianping Liu, Nanqi Zhao, Liyan Jia, Xiaoyi Yan, Fei Zhen, Zhaolan Liu, Jianping Liu, Jinhyang Ahn, Younghee Yun, Sulaiman AlEidi, Ashry Gad Mohamed, Abdullah M. Al-Beda, Raid A. Abutalib, Mohemmed K. M. Khalil, Hakima Amri, Sathyanarayana Badekila, Elham Behmanesh, Seyyedali Mozaffarpour, Elham Behmanesh, Seyyedali Mozaffarpour, Elham Behmanesh, Pantea Shirooye, Razie N. Meybodi, Roshanak Mokaberinejad, Mojgan Tansaz, Seyyedali Mozaffarpour, Vincent C. H. Chung, Xin Y. Wu, Justin C. Y. Wu, Babak Daneshfard, Ayda Hosseinkhani, Vahid Tafazoli, Amir M. Jaladat, Amir M. Jaladat, Hasan Sadeghi, Liyan Jia, Nanqi Zhao, Xiaoyi Yan, Li Zhou, Meng Zhao, Weiwei Li, Jianping Liu, Zhaolan Liu, Liyan Jia, Nanqi Zhao, Xiaoyi Yan, Li Zhou, Meng Zhao, Weiwei Li, Jianping Liu, Zhaolan Liu, Anette L. Larsen, Anita Salamonsen, Agnete E. Kristoffersen, Torunn Hamran, Bjørg Evjen, Trine Stub, Meiling Li, Jianxiong Cai, Taoying Lu, Lingjia Yin, Darong Wu, Lixin Wang, Siaw M. Liew, Tiegang Liu, Chen Bai, Zian Zheng, Yuxiang Wan, Jingnan Xu, Xuan Wang, He Yu, Xiaohong Gu, Zhaolan Liu, Xiaoyi Yan, Liyan Jia, Nanqi Zhao, Guoyan Yang, Jianping Liu, Seyyedali Mozaffarpour, Elham Behmanesh, Majid Nimrouzi, Vahid Tafazoli, Babak Daneshfard, Deja Ostrowski, Kealoha Fox, Mehdi Pasalar, Fatemeh Tabatabei, Fatemeh Amini, Saravanan Sathasivampillai, Pholtan Rajamanoharan, Michael Munday, Michael Heinrich, Yvonne M. Scherrer, Michael Heinrich, Carolyn Szuter, Fatemeh Amini, Fatemeh Tabatabaei, Ali Tavakoli, Fatemeh Tavakoli, Mehdi Pasalar, Mahsa rostami, Maria C. Torri, Carolyn Szuter, Harald Walach, Faith Warner, Anne Majumdar, Palitha Serasingh, Xiaoyi Yan, Liyan Jia, Nanqi Zhao, Zhaolan Liu, Jianping Liu, Nanqi Zhao, Fei Zhen, Liyan Jia, Xiaoyi Yan, Zhaolan Liu, Jianping Liu, Annemarie Abbing, Anne Ponstein, Erik Baars, Sarah Croke, Suzanne Hanser, Viola Heckel, Daniel Krüerke, Ana P. Simões-Wüst, Sebastian Weiss, Susanne Metzner, Jang W. Lee, Min K. Hyun, Morgana Masetti, Renate Oepen, Harald Gruber, Peter Heusser, Holger Pelz, Volker Perlitz, Anne Ponstein, Annemarie Abbing, Erik Baars, Nicola Robinson, Patricia Ronan, Awais Mian, Su Madge, Ava Lorenc, Penny Agent, Siobhan Carr, Patricia Ronan, Nicola Robinson, Siobhan Carr, Awais Mian, Ava Lorenc, Penny Agent, Su Madge, Monica E. Winnubst, Ricardo Monezi, Jafar Abolghasemi, Mojtaba Heydari, Sonia Baccetti, Elio Rossi, Paolo Fedi, Mariella Di Stefano, Katia Belvedere, Sonia Baccetti, Elio Rossi, Fabio Firenzuoli, Mariella Di Stefano, Katia Belvedere, Katherine Beaven, Anita Rose, Gerhard Florschutz, Nicola Brough Phil, Helen Parsons, Sarah Stewart-Brown, Katherine Burke, Martine Busch, Fenna Heyning, Jan Smit, Hans Jeekel, Hans de Goeij, Paulo Caceres Guido, Norma Barraza, Ziomara Balbarrey, Alejandra Ribas, Beatriz Jimenez, Claudia Iachino, Fabiana Quattrone, Marisa Gaioli, Marta Dell’Orso, Silvia Villanueva, Carmen Rocha, Adriana Macchi, Jianxiong Cai, Lina Chen, Darong Wu, Sicheng Wang, Eunji Choi, Namgyeong Go, Yongho Lee, Gokarna Dahal, Xaver Frauenknecht, Heike Gerhardt, Mónica Galanti, Carmen J. Cerda, Mónica Galanti, Mónica Galanti, Daniela Navarrete Heckersdorf, Héctor Jorquera, María L. Alcázar Saldivia, Daiva Jakubonienė, Bradley McEwen, Francislete Melo, Fernanda Mulinari Fontana, Ana C. Viana Valle, Maria T. Borges Neres, Abolali Mohagheghzadeh, Mohammad E. Zohalinezhad, Olja Njaradi, Momir Dunjic, Olja Njaradi, Momir Dunjic, Deja Ostrowski, Kealoha Fox, Jitka Pokladnikova, Iva Selke-Krulichova, Jinsoon Seo, Hyunchul Jang, Ana P. Simões-Wüst, Carolina Moltó-Puigmartí, Martien van Dongen, Pieter Dagnelie, Carel Thijs, Eva Tihanyi, Gabriella Hegyi, Ying Zhang, Xun Li, Yutong Fei, Jianping Liu, Ying Zhang, Jianping Liu, Xiaolin Tong

**Affiliations:** 1 Institute for Social Medicine, Epidemiology and Health Economics, Charité University Hospital, Berlin, Germany; 2 Research Centre for Traditional Medicine and History of Medicine, Shiraz University of Medical Sciences, Shiraz, Iran, Islamic Republic of; 3 Molecular Dermatology Research Centre, Shiraz University of Medicine, Shiraz, Iran, Islamic Republic of; 4 Medicinal Plants Processing Research Center, Shiraz University of Medical Sciences, Shiraz, Iran, Islamic Republic of; 5 Basic Sciences in Infectious Diseases Research Center, Shiraz University of Medical Sciences, Shiraz, Iran, Islamic Republic of; 6 Department of Epidemiology, Shiraz University of Medical Sciences, Shiraz, Iran, Islamic Republic of; 7 Research Centre for Traditional Medicine and History of Medicine, Shiraz University of Medical Sciences, Shiraz, Iran, Islamic Republic of; 8 Molecular Dermatology Research Center, Shiraz University of Medical Sciences, Shiraz, Iran, Islamic Republic of; 9 Health Policy Research Center, Shiraz University of Medical Sciences, Shiraz, Iran, Islamic Republic of; 10 Research Centre for Traditional Medicine and History of Medicine, Shiraz University of Medical Sciences, Shiraz, Iran, Islamic Republic of; 11 Health Policy Research Center, Shiraz University of Medical Sciences, Shiraz, Iran, Islamic Republic of; 12 Center for Nanotechnology in Drug Delivery, School of Pharmacy, Shiraz University of Medical Sciences, Shiraz, Iran, Islamic Republic of; 13 Ospedale Beata Vergine, Mendrisio, 6850 Switzerland; 14 NKS Kløveråsen as, Bodø, 8076 Norway; 15 RELIS North Norway, University Hospital North Norway, Tromsø, 9037 Norway; 16 Department of Pharmacy, UiT The Arctic University of Norway, Tromsø, Norway; 17 National Research Center in Complementary and Alternative Medicine (NAFKAM), Department of Community Medicine, The Arctic University of Norway UiT, Tromsø, 9037 Norway; 18 Homeopatic Clinic ASL Tuscany North West Lucca, Tuscan network for Integrative Medicine, Lucca, 55100 Italy; 19 Tuscan Network of Integrative Medicine, Florence, 50139 Italy; 20 Homeopatic Clinic ASL Tuscany North West Lucca, Tuscan network for Integrative Medicine, Lucca, 55100 Italy; 21 Homeopatic Clinic ASL Tuscany North West Lucca, Tuscan network for Integrative Medicine, Florence, Italy; 22 Tuscany Network of Integrative Medicine, Florence, 50139 Italy; 23 Regional Centre for Clinical Risk Management, Tuscany Region, Florence, 50139 Italy; 24 Pediatric Oncology, Charité University Hospital, Berlin, 10117 Germany; 25 Institute of Public Health, Charité University Hospital, Berlin, 13353 Germany; 26 Institute for Integrative Medicine, Community Hospital Herdecke, Herdecke, Germany; 27 Department of Community Medicine, National Research Center in Complementary and Alternative Medicine, UiT The Arctic University of Norway, Tromsø, 9037 Norway; 28 Research Institute Havelhöhe, Berlin, 14089 Germany; 29 Hospital Havelhöhe, Berlin, Germany; 30 Institut für wissenschaftliche Homoeopathie, Berlin, 13505 Germany; 31 Department of Pediatrics, Fribourg Hospital HFR, Fribourg, 1708 Switzerland; 32 Filderklinik, ARCIM Institute, Filderstadt, Germany; 33 Dept of Pediatrics, University Hospital Tübingen, Tübingen, Germany; 34 Department of Medicine, University of Fribourg, Fribourg, Switzerland; 35 Mental Health, Northern State Medical University, Arkhangelsk, Russian Federation; 36 Obstetrics, University Hospital Zurich, Zurich, 8091 Switzerland; 37 Weleda AG, Arlesheim, 4144 Switzerland; 38 Xiyuan Hospital of China Academy of Chinese Medical Sciences, Oncology Department, Beijing, China; 39 Department of Korean Gynecology, Kyung Hee University Hospital at Gangdong, Seoul, 05278 South Korea; 40 Chengdu University of Traditional Chinese Medicine, School of Acupuncture, Chengdu, China; 41 ULCA, David Geffen School of Medicine, Center for East West Medicine, Los Angeles, CA USA; 42 Department of Korean Medicine Dermatology, Kyung Hee University Hospital at Gangdong, Seoul, 134-806 South Korea; 43 Department of Biofunctional Medicine & Diagnostics, Kyung Hee University Korean Medicine Hospital, Seoul, South Korea; 44 Department of Science in Korean Medicine, Graduate School, Kyung Hee University, Seoul, South Korea; 45 Department of Preventive Medicine, Graduate School, Kyung Hee University, Seoul, South Korea; 46 Chinese Medicine Department, Hospital Authority, Hong Kong, Hong Kong; 47 College of Nursing, University of Manitoba, Winnipeg, R3T 2N2 Canada; 48 The Scarborough Hospital, Toronto, Canada; 49 Leslie Dan Faculty of Pharmacy, Toronto, Canada; 50 Mayo Clinic, Rochester, MN USA; 51 Chinese medicine, Changhua Christian Hospital, Changhua, 500 Taiwan; 52 Jockey Club School of Public Health and Primary Care, The Chinese University of Hong Kong, Shatin, Hong Kong Hong Kong; 53 The Chinese University of Hong Kong, Hong Kong Institute of Integrative Medicine, Hong Kong, Hong Kong; 54 Department of Medicine and Therapeutics, The Chinese University of Hong Kong, Shatin, Hong Kong Hong Kong; 55 University of Kentucky, Lexington, KY USA; 56 Simonton Cancer Center, Ulm, 89077 Germany; 57 AREMA, Strasbourg, 67000 France; 58 Unistra, Strasbourg, 67000 France; 59 Department of Integrative Medicine, Faculty of medicine Osijek, Osijek, 31000 Croatia; 60 Campus Avançado de Extensão Santo Amaro, UNIFESP, São Paulo, 04753060 Brazil; 61 Setor Espiritualidade e Saúde, Fundação Mokiti Okada, São Paulo, 04015-050 Brazil; 62 Religious Studies, Methodist University of São Paulo, São Bernardo do Campo, 09641-000 Brazil; 63 Departamento de Ciências Biológicas, Universidade Federal de São Carlos, São Carlos, 13565-905 Brazil; 64 UNIFESP, São Paulo, 04753060 Brazil; 65 Daofa, Komenda, 1218 Slovenia; 66 Office of Research, Endeavour College of Natural Health, Fortitude Valley, 4006 Australia; 67 Curso Técnico em Massoterapia, Escola de Educação Profissional Cecília Meireles – SEG, Porto Alegre, Brazil; 68 UNIFESP, São Paulo, 04753060 Brazil; 69 Fisioterapia, Centro Universitário Metodista-IPA, Porto Alegre, 90420-004 Brazil; 70 SMB ITALIA, Sanremo, 18038 Italy; 71 SIOMI, Integrative Medicine School, Firenze, Italy; 72 Istituto Europeo di Medicina Osteopatica, Osteopathy School, Genova, Italy; 73 COME Collaboration ONLUS, Fondazione per la Ricerca in Ambito Osteopatico, Vasto, Italy; 74 Acupuncture Centre of IOTHCFMSUP, São Paulo, 05410001 Brazil; 75 Acupuncture Centre of Orthopaedic and Traumatology Institute of Hospital das Clinicas, Medical School, São Paulo University, São Paulo, Brazil; 76 Primary Care, University of Southampton, Southhampton, SO16 5ST United Kingdom; 77 Center for Evidence Based Chinese Medicine, Beijing University of Chinese Medicine, Beijing, China; 78 Changying Community Health Service Center, Beijing, China; 79 Evidence-based medicine center, Beijing University of Chinese Medicine, Beijing, China; 80 Dongfang Hospital, Beijing University of Chinese Medicine, Beijing, China; 81 Kyung Hee University Hospital at Gangdong, Seoul, 05278 South Korea; 82 National Center for Complementary and Alternative Medicine, Riyadh, Saudi Arabia; 83 Family and Community Medicine Department, King Saud University, Riyadh, Saudi Arabia; 84 Orthopedic Surgery, King Fahad Hospital, Madinah, Saudi Arabia; 85 Biochemistry and Cellular and Molecular Biology, Georgetown University Medical Center, Washington, D.C, USA; 86 Ayurveda, Muniyal Institute of Ayurveda Medical Sciences, Manipal, 576104 India; 87 School of Traditional Medicine and History of Medical Science Research Center, University of Medical Sciences, Babol, 47176477 Iran, Islamic Republic of; 88 Traditional Medicine, University of Medical Science, Babol, 4717647745 Iran, Islamic Republic of; 89 Traditional Medicine, University of Medical Science, Babol, 4717647745 Iran, Islamic Republic of; 90 Traditional Medicine, Shahid eheshti University of Medical Science, Tehran, 1516745811 Iran, Islamic Republic of; 91 Jockey Club School of Public Health and Primary Care, The Chinese University of Hong Kong, Shatin, Hong Kong Hong Kong; 92 Student Research Committee, Shiraz University of Medical Sciences, Shiraz, Iran, Islamic Republic of; 93 Research Center for Traditional Medicine and History of Medicine, Shiraz University of Medical Sciences, Shiraz, Iran, Islamic Republic of; 94 Shiraz University of Medical Sciences, Shiraz, Iran, Islamic Republic of; 95 Center for Evidence-based Chinese Medicine, Beijing University of Chinese Medicine, Beijing, China; 96 Dongzhimen hospital, Beijing University of Chinese Medicine, Beijing, China; 97 Xiyuan hospital, Beijing University of Chinese Medicine, Beijing, China; 98 Center for Evidence-based Chinese Medicine, Beijing University of Chinese Medicine, Beijing, China; 99 Dongzhimen hospital, Beijing University of Chinese Medicine, Beijing, China; 100 Xiyuan hospital, Beijing University of Chinese Medicine, Beijing, China; 101 Department of Community Medicine, NAFKAM, The Arctic University of Norway UiT, Tromsø, 9037 Norway; 102 Department of Health and Care Sciences, The Artic University of Norway, Tromsø, 9037 Norway; 103 Centre for Sami studies, The Actic University of Norway, Tromsø, 9037 Norway; 104 Guangzhou University of Chinese Medicine, Guangzhou, 510000 China; 105 The Second Affiliated Hospital of Guangzhou University of Chinese Medicine, Guangzhou, 510120 China; 106 The Affiliated Hospital of Changchun University of Chinese Medicine, Changchun, China; 107 Cyberjaya University College of Medical Sciences, Cyberjaya, Malaysia; 108 Beijing University of Chinese Medicine, Beijing, 100029 China; 109 Beijing University of Chinese Medicine, Center for Evidence-based Chinese Medicine, Beijing, China; 110 Traditional Medicine and Medical history Research Centre, University of Medical Science, Babol, Iran, Islamic Republic of; 111 Shiraz University of Medical Sciences, Shiraz, Iran, Islamic Republic of; 112 Office of Hawaiian Affairs, Honolulu, 96817 HI USA; 113 Research Centre for Traditional Medicine and History of Medicine, Shiraz University of Medical Sciences, Shiraz, Iran, Islamic Republic of; 114 School of Phramacy, University College London, London, WC1N 1AX United Kingdom; 115 Provincial Department of Indigenous Medicine, Trincomalee, Sri Lanka; 116 Sustainability Research Group, University of Basel, Basel, 4051 Switzerland; 117 Research Group ‘Pharmacognosy and Phytotherapy’, Univ. London, School of Pharmacy, London, WC1N 1AX United Kingdom; 118 Sociology, University of New Brunswick, Fredericton, E3B4H7 Canada; 119 School of Medicine, Traditional Medicine, Shiraz, Iran, Islamic Republic of; 120 Research Center for Traditional Medicine and History of Medicine, Shiraz University of Medical Sciences, Shiraz, Iran, Islamic Republic of; 121 Sociology, University of New Brunswick, Fredericton, E3B4H7 Canada; 122 Pediatric Gastroenterology, Poznan Medical University, Poznan, 60-572 Poland; 123 Middlesex University, London, NW4 4BT United Kingdom; 124 SHAS, St Mary’s University Twickenham, Twickenham, TW1 4SX United Kingdom; 125 Center for Evidence-based Chinese Medicine, Beijing University of Chinese Medicine, Beijing, China; 126 Evidence-based medicine center, Beijing University of Chinese Medicine, Beijing, China; 127 Dongfang Hospital, Beijing University of Chinese Medicine, Beijing, China; 128 Anthroposphic Health Care, University of applied sciences, Leiden, 2300 AJ Netherlands; 129 School of Heathcare, University of Leeds Alumnus, Rochdale, OL12 7RX United Kingdom; 130 Music Therapy, Berklee College of Music, Boston, 02215 USA; 131 Klinik Arlesheim, Arlesheim, 4144 Switzerland; 132 Klinik Öschelbronn, Niefern-Öschelbronn, Germany; 133 University of Augsburg, Augsburg, Germany; 134 College of Korean Medicine, Dongguk University, Goyang, South Korea; 135 Preventive Medicine, College of Korean Medicine, Dongguk University, Gyeongju, South Korea; 136 Psychology, Pontificia Universidade Católica de Sao Paulo, Rio de Janeiro, 22430 Brazil; 137 Research Institute for Creative Arts Therapies (RIArT), Alanus University of Arts and Social Sciences, Alfter/Bonn, 53347 Germany; 138 Theory of Medicine, Integrative and Anthroposophic Medicine, Witten/Herdecke University, Herdecke, 58313 Germany; 139 DGOM e.V./HGMB i.G, Buxtehude, 21614 Germany; 140 Anthroposphic Health Care, University of applied sciences, Leiden, 2300 AJ Netherlands; 141 School of Health and Social Care, London South Bank University, London, SE1 0AA United Kingdom; 142 Royal Brompton and Harefield NHS Trust, London, United Kingdom; 143 School of Health and Social Care, London South Bank University, London, SE1 0AA United Kingdom; 144 Royal Brompton and Harefield NHS Trust, London, United Kingdom; 145 UNIFESP, Sao Paulo, 04753-060 Brazil; 146 Research Centre for Traditional Medicine and History of Medicine, Shiraz University of Medical Sciences, Shiraz, 71348457 Iran, Islamic Republic of; 147 Centre of Acupuncture and Traditional Chinese Medicine, Tuscan network for Integrative Medicine, Florence, 50133 Italy; 148 Tuscan network for Integrative Medicine, Florence, 50139 Italy; 149 Health Directorate, Region of Tuscany, Florence, 50139 Italy; 150 Centre of Acupuncture and Traditional Chinese Medicine, Tuscan network for Integrative Medicine, Florence, 50133 Italy; 151 Tuscan Network of Integrative Medicine, Florence, 50139 Italy; 152 Health Directorate, Region of Tuscany, Florence, 50139 Italy; 153 Eurythm Therapy, Raphael Medical Centre, Hildenborough, United Kingdom; 154 Warwick Medical School, University of Warwick, Coventry, CV4 7AL United Kingdom; 155 Office of Public Health Studies, University of Hawaii, Honolulu, HI USA; 156 Van Praag Instituut, Utrecht, 3511 VH Netherlands; 157 STZ Association of Teaching Hospitals, Utrecht, Netherlands; 158 Internal Medicine, Radboud University Medical School, Nijmegen, Netherlands; 159 Surgery, Erasmus University Medical School, Rotterdam, Netherlands; 160 Ministery of Health, Den Haag, Netherlands; 161 Hospital de Pediatria Garrahan, Universidad Maimonides, Buenos Aires, 1245 Argentina; 162 Universidad Maimonides, Buenos Aires, Argentina; 163 The TCM Clinical Outcome Assessment Team, The Second Affiliated Hospital of Guangzhou University of Chinese Medicine, Guangzhou, 510120 China; 164 Science and Technology Division, State Administration of Traditional Chinese Medicine of the People of China, Beijing, 100027 China; 165 ONBODYCLINIC, Seoul, South Korea; 166 Department of Ayurveda, Ministry of Health, Kathmandu, Nepal; 167 Sozialstiftung Bamberg, Bamberg, 96049 Germany; 168 Public Policies, Ministry of Health, Santiago, 8320000 Chile; 169 Pediatric, University of Chile, Santiago, Chile; 170 Ministry of Health, Public Policies, Santiago, Chile; 171 University of Chile, Pediatric, Santiago, Chile; 172 Public Policies, Ministry of Health, Santiago, Chile; 173 Department of Quality of Life and Labor Relations, Chilean Health Ministry, Santiago, Chile; 174 Chilean Health Service, Santiago, Chile; 175 Department of health explicit guarantees and High Complexity Networks, Chilean Health Ministry, Santiago, Chile; 176 Natūraliosios terapijos centras, Vilnius, 08119 Lithuania; 177 Department of Nutritional Medicine, Endeavour College of Natural Health, Sydney, 2000 Australia; 178 Pharmacy, UPIS, Brasília, 70390-12 Brazil; 179 Natural Pet, Brasília, Brazil; 180 Museum, Archives and Cultural Studies of Norani Vesal, Shiraz University of Medical Sciences, Shiraz, Iran, Islamic Republic of; 181 Pharmacognosy, Shiraz University of Medical Sciences, Shiraz, Iran, Islamic Republic of; 182 BDORT Center, Belgrade, 11000 Serbia; 183 BDORT Center, Belgrade, 11000 Serbia; 184 Office of Hawaiian Affairs, Honolulu, HI USA; 185 Charles University, Hradec Kralove, 50005 Czech Republic; 186 Korea Institute of Oriental Medicine, Daejeon, South Korea; 187 Obstetrics, University Hospital Zurich, Zurich, Switzerland; 188 Research, Clinic Arlesheim, Arlesheim, Switzerland; 189 Epidemiology,CAPHRI School for Public Health and Primary Care, Maastricht University, Maastricht, Netherlands; 190 CARIM School for Cardiovascular Disease, Maastricht University, Maastricht, Netherlands; 191 Faculty of Health Sciences, University of Pecs, Pecs, Hungary; 192 Confucius Institute of TCM, University of Pecs, Pecs, Hungary; 193 Center for Evidence Based Chinese Medicine, Beijing University of Chinese Medicine, Beijing, China; 194 Center for Evidence Based Chinese Medicine, Beijing University of Chinese Medicine, Beijing, China; 195 China Academy of Chinese Medical Sciences, Guang’anmen Hospital, Beijing, China

## P245 Complementary and Integrative Medicine in nursing homes – effects on caregivers in a prospective, exploratory, comparative, two-armed cohort study

### Miriam Ortiz, Katharina Schnabel, Michael Teut, Gabriele Rotter, Sylvia Binting, Margit Cree, Fabian Lotz, Ralf Suhr, Benno Brinkhaus

#### *Institute for Social Medicine, Epidemiology and Health Economics, Charité University Hospital, Berlin, Germany*

##### **Correspondence:** Miriam Ortiz (miriam.ortiz@charite.de)

**Question**

"Kneipp Therapy" (KT) is a form of Complementary and Integrative Medicine (CIM) that includes a combination of hydrotherapy, herbal medicine, mind-body medicine, physical activities and healthy nutrition. Several Nursing homes (NH) in Germany started to integrate KT in the routine care of residents. The aim of this study was to investigate if caring with KT has effects on caregivers.

**Methods**

We conducted a prospective, exploratory, two-armed cohort study to compare NH with (KT group) and without KT (but with routine health preventive interventions; control group) over 12 months. Each NH with KT was matched to a control. Outcomes for caregivers included the SF-12 Health Survey, the Work Ability Index (WAI) and the Copenhagen Psychosocial Questionnaire (COPSOQ).

**Results**

Altogether 111 caregivers were included from 7 NH (KT group, *n* = 48) respectively 6 NH (control group, *n* = 63). 95% of caregivers were female (43.4 ± 11.3 years, BMI 26.6 ± 5.3). At baseline, caregivers of the KT group showed better values for the COPSOQ scales "feedback" (*p* = 0.043), "job satisfaction" (*p* < 0.001) and "burnout" (*p* = 0.008). After 6 months the control group was better in the COPSOQ scales "predictability of work" (*p* = 0.045) and after 12 months for "sense of community" (*p* = 0.047) and the physical component scale of the SF-12 (*p* = 0.039) compared to the KT group.

**Conclusions**

The study showed only minor differences between the caregivers of both groups. Caregivers´ workability, quality of life and psychosocial burden at work seems to be less influenced by a CIM-oriented working-place approach.

Trial Registration DRKS-ID: DRKS00005049

## P246 Efficacy of adjuvant topical Juniperus excelsa versus cryotherapy alone in the treatment of cutaneous leishmaniasis: a double-blind randomized controlled clinical trial

### Mohammad M Parvizi^1,2^, Farhad Handjani^2^, Mohammad M Zarshenas^3^, Mahmood R Moein^3^, Majid Nimrouzi^1^, Gholamreza Hatam^4^, Jafar Hasanzadeh^5^, Nasrin Hamidizadeh^2^

#### ^*1*^*Research Centre for Traditional Medicine and History of Medicine, Shiraz University of Medical Sciences, Shiraz, Iran, Islamic Republic of*; ^2^*Molecular Dermatology Research Centre, Shiraz University of Medicine, Shiraz, Iran, Islamic Republic of*; ^*3*^*Medicinal Plants Processing Research Center, Shiraz University of Medical Sciences, Shiraz, Iran, Islamic Republic of*; ^*4*^*Basic Sciences in Infectious Diseases Research Center, Shiraz University of Medical Sciences, Shiraz, Iran, Islamic Republic of;*^*5*^*Department of Epidemiology, Shiraz University of Medical Sciences, Shiraz, Iran, Islamic Republic of*

##### **Correspondence:** Mohammad M Parvizi

**Background**

in vitro and in vivo evidences show anti-leishmaniasis effects of *Juniperus excels* (JE)*.* The aim of this study is determination of efficacy of topical application of *Juniperus excels* extract in as adjuvant of cryotherapy in human cutaneous leishmaniasis (CL).

**Methods**

The study was designed as a two-arm triple-blind randomized placebo-controlled clinical trial using a parallel design. Seventy two patients with clinical diagnosis of CL confirmed by cytology allocated to receive either a topical formulation of JE or placebo (1: 1 allocation ratio) for 3 months. Both groups received cryotherapy as baseline standard treatment. Patients were evaluated before and weekly after the intervention according to size of lesions.

**Results**

There was a significantly greater decrease in mean size of lesions 3 months in the JE than placebo group (100.89 ± 14.58 vs 217.62 ± 31.73, *P* < 0.001). In addition, the multinomial logistic regression reveal that the rate of complete cure in patient who received the drug were 13 times in comparing with who received placebo (OR = 13.50, 95% CI 3.210-56.770, *P*-value < 0.001). Five out of 33 patient of JE group, showed drug hypersensitivity including redness and itching after about 5 weeks consumption (delay regional reaction to drug).

**Conclusions**

Application of a topical formulation of JE extract can decrease size of lesions and increase complete cure rate in patients with CL.

## P247 Successful treatment of chronic seborrheic dermatitis with topical *Althaea officinalis L.* leaf: a case report

### Mohammad M Parvizi^1,2^, Mojtaba Heydari^1^, Mohammad R Namazi^2^, Zahra Parvizi^3^

#### ^*1*^*Research Centre for Traditional Medicine and History of Medicine, Shiraz University of Medical Sciences, Shiraz, Iran, Islamic Republic of*; ^*2*^*Molecular Dermatology Research Center, Shiraz University of Medical Sciences, Shiraz, Iran, Islamic Republic of;*^*3*^*Health Policy Research Center, Shiraz University of Medical Sciences, Shiraz, Iran, Islamic Republic of*

##### **Correspondence:** Mohammad M Parvizi (mmparvizi@gmail.com)

Seborrheic dermatitis (SD) is a common, chronic and relapsing disorder caused by changes in the cutaneous microflora. This disease is called *hozaz* in traditional Persian medicine. Topical consumption of *Althaea officinalis* L. in combination with vinegar is traditionally recommended for treatment of this disease. This report presents a case of successful treatment of a 32 female patient with seborrheic dermatitis with topical application of *Althaea officinalis* L. in combination with vinegar.The patient was instructed to take topical product of dry leaf of *Althaea officinalis L.* (Katmi) mixed with grape vinegar in lotion form on scaling parts of head and hairs for 20 minutes. This intervention was made based on the diagnosis of *hozaz*, according to TPM. No other drug was administrated for the patient.After 4 month fallowing up a complete response was achieved; she did not have any sign of scaliness in her scalp. No sign and/or symptom such as pruritus, erythema, scurf and crust, redness and inflammation were present in this visit.According to this positive solitary clinical experience, the effect of *Althaea officinalis* L. in treatment of seborrheic dermatitis should be evaluated by more rigorous methods such as randomized controlled trials.

**Consent**

Informed consent for publication was given by the patient.

## P248 Quality of life and anise oil; a promising effect based on Traditional Persian Medicine priciples

### Mehdi Pasalar^1^, Maryam Mosaffa-Jahromi^1^, Kamran Bagheri-Lankarani^2^, Suleiman Afsharypuor^1^, Ali M Tamaddon^3^, Mohadeseh Ostovar^1^

#### ^*1*^*Research Centre for Traditional Medicine and History of Medicine, Shiraz University of Medical Sciences, Shiraz, Iran, Islamic Republic of*; ^*2*^*Health Policy Research Center, Shiraz University of Medical Sciences, Shiraz, Iran, Islamic Republic of;*^*3*^*Center for Nanotechnology in Drug Delivery, School of Pharmacy, Shiraz University of Medical Sciences, Shiraz, Iran, Islamic Republic of*

##### **Correspondence:** Mehdi Pasalar

**Purpose**

Anise oil has been recommended for the treatment of bowel disorders in Persian medical resources. Based on traditional Persian medicine (TPM) sages, this ingredient could improve gastrointestinal diseases in patients. The aim of this study was to determine the effect of enteric coated capsules of anise oil (AnisEncap) on quality of life in patients with irritable bowel syndrome (IBS).

**Methods**

In a three-armed double-blind clinical trial, 120 patients were allocated into three groups by block randomization: AnisEncap, placebo and Colpermin®. They received 1 capsule per day for 4 weeks and filled valid IBS-quality of life questionnaires out before, after and succeeding 2 weeks follow-up visit.

**Results**

All groups showed similar insignificant demographic characteristics at the end. The effectiveness of AnisEncap in improving IBS patients’ quality of life (in the form of mean total score) was significantly superior to placebo or Colpermin® after the 4-week treatment and the 2-week follow-up time (*P* < 0.001) (Table 1).

**Table 1 (Abstract P247).** Mean scores with standard deviation on IBS-QOL questionnaire in IBS patients

IBS-QOL mean total score	AnisEncap	Placebo	Colpermin®	P value
Baseline	86.20 ± 27.13	83.30 ± 21.64	77.25 ± 25.34	0.261
After 4-week treatment	64.38 ± 23.10	72.82 ± 18.20	69.50 ± 22.93	0.000
After 2-week follow-up	60.45 ± 23.79	70.05 ± 19.51	66.45 ± 23.06	0.000

**Conclusions**

The effectiveness of AnisEncap was better than Colpermin® or placebo in patients with IBS regarding quality of life. Further studies are suggested to find other natural compounds suitable for upgrading the quality of life in different types of diseases.

Trial Registration: Clinical.Trials.gov (NCT02364830)

Key words: Anise, Quality of Life, Traditional Persian Medicine

## P249 Integrative rehabilitation program for elderly at high risk of falling

### Giuseppe Peloni, Ingo Bolliger, Rui M Da Cunha Faria, Pierluigi Quadri, Wilma Sanzeni, Damiano Zemp

#### Ospedale Beata Vergine, Mendrisio, 6850, Switzerland

##### **Correspondence:** Giuseppe Peloni (giuseppe.peloni@eoc.ch)

**Question**

Gait and balance disorders belong to the most common causes of falls in older adults and often lead to injuries and disability, affecting independence and quality of life. Physical activity in particular balance and strength training of lower limbs is known to be effective in fall prevention programs. The aim of this study is to evaluate the integration of acupuncture in outpatient rehabilitation program for elderly with increased risk of falling.

**Methods**

From 2013 to 2016, 60 frail older patients underwent physiotherapy and occupational therapy for two months 2 times a week. 45 participants concluded the program. 18 among them followed additionally acupuncture sessions once a week. The fall risk assessment performed before and after treatment included Timed Up and Go test, Short Physical Performance Battery (SPPB), computerized posturography and gait analysis under single- and dual-task condition.

**Results**

Mean age was 81 (62-94) years. Mini Mental State Examination was 26.6. Neither at baseline nor in follow-up assessments, acupuncture differ from the no-acupuncture group. In general functional ability in particular SPPB (p < 0.05) improved, some posturography parameters improved but not significantly and no parameters of gait analysis changed significantly.

**Conclusions**

Improvement in functional tests despite low significance suggests that therapeutic intervention could decrease risk of falling. For better understanding the relevance of the integration of acupuncture in falls prevention programs, further studies with a larger number of patients and a better standardization in recruitment, assessment and intervention are needed.

## P250 Are dietary supplements for patients with dementia risk free? Results of a survey conducted in a Norwegian memory clinic

### Hilde Risvoll^1^, Trude Giverhaug^2^, Kjell H Halvorsen^3^, Marit Waaseth^3^, Frauke Musial^4^

#### ^*1*^*NKS Kløveråsen as, Bodø, 8076, Norway*; ^*2*^*RELIS North Norway, University Hospital North Norway, Tromsø, 9037, Norway;*^*3*^*Department of Pharmacy, UiT The Arctic University of Norway, Tromsø, Norway; National Research Center in Complementary and Alternative Medicine (NAFKAM), Department of Community Medicine, The Arctic University of Norway UiT, Tromsø, 9037, Norway*

##### **Correspondence:** Hilde Risvoll (hilde.risvoll@uit.no)

**Background**

The use of dietary supplements (DS) is common among patients with dementia. Direct risks associated with DS use are for instance adverse events and DS-drug interactions. However, the impaired cognitive functioning of persons with dementia can be associated with indirect risk. The aim of this study was to describe the extent and risk structure of DS use of persons with dementia in ambulatory care.

**Methods**

We conducted a survey among 151 patients with dementia attending an outpatient memory clinic in Northern Norway. Study measurements included patient characteristics, cognitive- and ADL-functioning (activities of daily living), and use of DS and prescription drugs (PD). We assessed direct risks by evaluating potential DS- drug interactions and indirect risks by evaluating conditions of use.

**Results**

Forty-six percent (*n* = 70) of the patients used DS. Ninety-seven percent (*n* = 147) used PD. Eight potentially clinically-relevant DS-drug interactions were identified. While only 36% (*n* = 26) of the patients received assistance with their DS, 73% (*n* = 106) received assistance with their PD. Patients living alone were at risk of not receiving assistance, while patients who scored worse on cognitive and ADL-functioning tests generally received more assistance with both DS and PD. Only one-third of the patients and half of the caregivers were aware of the risks of side effects and interactions concerning DS use.

**Conclusions**

Patients with dementia use DS frequently. DS use may be associated with direct and indirect risks to patient safety as potentially clinically relevant interactions were discovered and DS intake was often unsupervised.

## P251 Complementary medicine in oncology: a multicenter retrospective study on 1928 patients with cancer in the Region of Tuscan (Italy)

### Elio Rossi^1^, Sonia Baccetti^2^, Marco Picchi^2^, Tommaso Conti^2^, Fabio Firenzuoli^2^, Carmelo Guido^2^, Filippo Bosco^2^, Carmelo Guido^2^

#### ^*1*^*Homeopatic Clinic ASL Tuscany North West Lucca, Tuscan network for Integrative Medicine, Lucca, 55100, Italy*; ^*2*^*Tuscan Network of Integrative Medicine, Florence, 50139, Italy*

##### **Correspondence:** Elio Rossi

**Purpose**

This multicenter retrospective observational study describes the results of complementary medicine (CM) treatment to reduce adverse effects of anti-cancer treatment and cancer symptoms, to improve the quality of life of cancer patients.

**Methods**

Data are concerning 1928 patients consecutively visited from 2013 till September 2016 at the CM Clinics of the Public Hospital of Lucca; Camerata-Florence; Careggi-Florence and Pisa. Preliminary data show that 78% of the patients were female and 22% male; mean age 56 years (35 –88) years.

**Results**

Main type or localization are: breast cancer (58.3%), colon (6.1%), lung (6.1%), ovary (4%), stomach (2.9%), prostate (2.5%), uterus (2.5%), brain (1.4%), kidney (1,1%), liver (1,1%) and pancreas (1,1%). 34% of patients had metastasis. Symptoms most frequently treated were adverse effects of anti-cancer therapies (55.1%), that is effects of chemotherapy (27.6%), hormonal therapy (12.1%), surgical therapy (10.3%) and radiotherapy (5.1%). The symptoms caused by the disease are 25.9%, and the concomitant symptoms 8.6%. The most frequent symptoms are: asthenia/fatigue, hot flashes, depression, neuropathy, nausea/vomiting after chemotherapy, irritable intestine, diarrhea, leucopenia, dermatitis, anxiety, radiodermitis. Comparing the clinical conditions before and after the treatment, we observed significant amelioration of nausea (*p* = 0.004); insomnia (*p* = 0.003); depression (*p* = 0.000); anxiety (*p* = 0.000); asthenia (*p* = 0.000); hot flushes (*p* = 0.000). Exclusive homeopathy seems to be less effective than an integrated therapy, but not statistically significant.

**Conclusions**

A clinic of integrative oncology seems to give the possibility to reduce adverse effects of anticancer therapy and ameliorate the quality of life of cancer patients

## P252 Women and complementary medicine: the experience of the homeopathic clinic for women in a public hospital

### Elio Rossi, Marialessandra Panozzo, Marco Picchi, Chiara Cervino, Linda Nurra

#### *Homeopatic Clinic ASL Tuscany North West Lucca, Tuscan network for Integrative Medicine, Lucca, 55100, Italy*

##### **Correspondence:** Elio Rossi

**Introduction**

In 2003, the Homeopathic Clinic for Women at Campo di Marte Hospital (now Cittadella della salute) was opened in Lucca, Italy. Over a 13-year period women mostly with gynaecological diseases were followed up.

**Purpose**

This paper explores the socio-demographic characteristics, main complaints, most commonly used integrative therapies, and the clinical results of women presenting over this period.

**Methods**

An observational, longitudinal study was conducted on 1516 women consecutively examined from 2003 to 2015. The ORIDL (Outcome in Relation to Impact on Daily Living) was used to assess outcome. All patients were treated with individualized homeopathic treatment (single remedy), without excluding other integrative treatments when necessary.

**Results**

Patients mean age was 42 years, most were office workers (23.9%); 33.4% had already used conventional therapies and 38% homeopathic remedies. The most frequently observed gynaecological diseases for 54%of the cases were: menopausal disorders (21.6%) and menstrual irregularities (11.9%), and among non gynaecological diseases, psychological disorders (12.9%).

A homeopathic prescription was followed by herbal therapy for 42.2% of the patients with menopausal disturbances; 53.6% women with gynaecological problems, received follow-up and 38.1% were women with menopausal disorders. An improvement was obtained in 74.1% of the patients; major improvement or resolution (ORIDL = 2, 3, 4) was seen in 61.2% of the women, 66.9% of these with menopausal disorders.

**Conclusion**

Homeopathic treatment was sometimes integrated with diet, botanicals, and psychological counselling, and support in psychopathological conditions, and demonstrated positive therapeutic effects, particularly for women with menopausal disorders.

## P253 Safety of the patient and clinical risk management in Complementary Medicine: Clinical audit and Failure Modes and Effects Analysis (FMEA)

### Elio Rossi^1^, Marco Picchi^2^, Fabio Firenzuoli^2^, Antonella Traversi^2^, Katia Vuono^2^, Federica Sabatini^2^, Tommaso Bellandi^3^

#### ^*1*^*Homeopatic Clinic ASL Tuscany North West Lucca, Tuscan network for Integrative Medicine, Florence, Italy*; ^*2*^*Tuscany Network of Integrative Medicine, Florence, 50139, Italy;*^*3*^*Regional Centre for Clinical Risk Management, Tuscany Region, Florence, 50139, Italy*

##### **Correspondence:** Elio Rossi

**Purpose**

To develop a plan for the management of clinical risk starting from the analysis of activities carried out in the homeopathic and acupuncture regional centres of reference using clinical audit and Failure Modes and Effects Analysis (FMEA), in order to develop a systematic approach aiming at identifying and preventing clinical risks.

**Methods**

FMEA is a proactive risk management tool organized into seven steps: selection of the clinical process to be analysed: organization of a multidisciplinary group of experts; description of the process; identification of Failure Modes (FMs) for each step; estimate of the frequency, severity and detectability of the FMs; calculation of the Risk Priority Number (RPN); prioritize the improvement actions to prevent the FMs. Moreover, a clinical audit must be performed at least for the most complex cases.

**Results**

In homeopathic clinics, the highest RPN focused on the decision to switch from an allopathic to a homeopathic therapy, possible solutions required coordination with other specialists involved in the care process for complex cases. In agopuncture major problems could arise from the modalities of needle insertion.

**Conclusions**

Our experience demonstrates that an in-deep analysis can reveal potential risks for the patients as well as the priorities for improvement actions to be taken in order to guarantee a safe and reliable service in homeopathic medicine. Besides, this technique proved to be sustainable in terms of time and use of resources. According to our experience, the application of FMEA is recommended in CM after a basic patient safety training program.

## P254 Establishing a concept of integrative care in a pediatric oncology unit of University hospital – challenges and changes

### Britta Rutert^1,2^, Angelika Eggert^1^, Georg Seifert^1^, Wiebke Stritter^1^, Christine Holmberg^2^, Alfred Längler^3^

#### ^*1*^*Pediatric Oncology, Charité University Hospital, Berlin, 10117, Germany*; ^*2*^*Institute of Public Health, Charité University Hospital, Berlin, 13353, Germany;*^*3*^*Institute for Integrative Medicine, Community Hospital Herdecke, Herdecke, Germany*

##### **Correspondence:** Britta Rutert

**Purpose**

The treatment of cancer in children has shown to have high success rates, but is often accompanied by physical and emotional episodes of pain and suffering. Biomedical medication may be able to release the pain but does have little positive effect on the suffering. Suffering may rather be relieved by more integrative methods of treatment. Anthroposophic treatment has proven to be helpful in the reduction of pain, sleeplessness or nausea. By applying these methods touch and affection will be re-integrated into care and may thus reduce feelings of fear in children and provide contentment in nurses. The aim of a project at a German university hospital is to implement anthroposophic care into standard care in pediatric oncology by developing and implementing an integrative care concept. The leading question is here: what are the challenges, and chances, of implementing an integrative concept of care in a pediatric oncology hospital?

**Method**

By using ethnographic research and qualitative as well as quantitative data, the study first analyzed the status quo of care at a pediatric oncology unit. Based on this mixed-method approach, a concept was developed to implement anthroposophic care into the care unit at a pediatric care hospital.

**Results**

First results have shown that nurses, patients and parents feel constraint by the tightly structure care, which hardly allows for the engagement with the patients beyond the necessary. Anthroposophic treatment may re-instigate touch and affection without making demands on additional time in care.

**Conclusion**

Integrative care may be regarded as beneficial by nurses, patients and parents. The implementation of integrative care, however, is not easy in a setting, which is dominated by time pressure, economic budgeting and lack of nurses.

## P255 Are differing risk perceptions related to patients’ use of CAM a barrier to doctor-patient communication in comprehensive patient care

### Anita Salamonsen, Solveig Wiesener

#### Department of Community Medicine, National Research Center in Complementary and Alternative Medicine, UiT The Arctic University of Norway, Tromsø, 9037, Norway

##### **Correspondence:** Anita Salamonsen

**Question**

The widespread and continuing use of CAM among Western patients will affect public healthcare delivery and doctor-patient communication for the foreseeable future. Studies have shown that doctor-CAM user communication often is challenging for both doctors and patients. We hypothesized that differing risk understandings may represent an important factor in this picture, and conducted a study on lay and medical risk perceptions and their possible implications. "Perceived risk" was here understood from a social science perspective, as based on a variety of factors involving individual experience, cultural beliefs, social practices, attitudes, and values.

**Methods**

Twenty-five Norwegian CAM-users diagnosed with cancer or multiple sclerosis and 12 of their doctors (five general practitioners, four oncologists and three neurologists) participated in qualitative, individual in-depth interviews.

**Results**

Most of the CAM users perceived CAM as "natural", "green"and "not involving risk". The doctors found such risk understandings difficult to discuss,because they differed fundamentallyfrom medical risk understandings based on science and clinical experience. From the CAM users’ perspective, the doctors often acted arrogantly and not patient-centered. In the research interviews, however, the doctors expressed many "patient-centered" concerns for patients who choose to use CAM, such as delay of importantconventional treatment, quality of life for cancer patients hunting for a cure, and possible economic burdens for patients and their families. Their concerns were most often not discussed with the patients, mainly because the doctors were unsure whether they as doctors in the public healthcare services should engage in these issues.

**Conclusions**

Currently, gaps in lay and medical risk perceptions seem to represent barriers to well-functioning doctor-CAM user communication. Recent white papers describe "the patients' healthcare services", which for many patients includes CAM use. This study suggests that doctors have many concerns for patients who use CAM, and that the health authorities should clarify whether or not doctors shall actively relate to patients’ CAM use in clinical encounters. If doctor-CAM-user communication includes both lay and medical views on risk and CAM, the goal of comprehensive patient care is within reach also in a patient safety perspective.

## P256 Application pattern of non-pharmacological interventions in breast cancer patients in a multimodal integrative oncology setting

### Friedemann Schad^1^, Megan Steele^1^, Matthias Kröz^1^, Harald Matthes^1^, Cornelia Herbstreit^2^, Anja Thronicke^1^

#### ^*1*^*Research Institute Havelhöhe, Berlin, 14089, Germany*; ^*2*^*Hospital Havelhöhe, Berlin, Germany*

**Question**

Non-pharmacological interventions (NPI) have known benefit for physical and psychological health and quality of life (HRQL) in breast cancer patients. The aim of this pivotal study was to explore the association of breast cancer and the application of NPI in a multimodal integrative oncology setting.

**Methods**

Clinical and demographic data from the Network Oncology registry were analysed for 5.046 patients with breast cancer versus 11.469 patients with other cancer entities. The free statistical software R was utilized for logistic regression investigations.

**Results**

The median age of breast cancer patients was 54 years. Adjusted multivariate regression analysis revealed that breast cancer patients twice more likely choose educational programs (OR 2.09, 95%CI: 1.72-2.54, *p* < 0.00001) and less likely choose breathing (OR 0.33, 95% CI 0.26-0.42, *p* < 0.00001), nursing (OR 0.50, 95% CI: 0.42-0.60, *p* < 0.00001) and art therapies (OR 0.81, 95% CI: 0.68 – 0.97, *p* = 0.022) compared to other cancer patients. The likeliness of choosing *Viscum album* L. (VA) was higher in breast cancer patients compared to patients with other cancer entities (OR 1.23, 95% CI: 1.11 – 1.36, *p* < 0.00001). Enhanced application of massage and psychotherapy and less utilization of movement therapies was not dependent on whether patients had breast or other cancer.

**Conclusions**

Our results show that breast cancer patients are substantially interested in educational integrative programs. Further, the utilization of psycho-oncological supportive programs and massage is equal in patients with breast cancer or other cancer entities. Physicians knowledge on entity-specific application patterns of NPI might be a key to improve HRQL in cancer patients.

## P257 Identifying the homeopathic simillimum: rubrics and the inner knowledge of the patient

### Irene Schlingensiepen (irene.schlingensiepen@quellenhomoeopathie.de)

#### *Institut für wissenschaftliche Homoeopathie, Berlin, 13505, Germany*

**Background**

With the concept of the simillimum, homeopathy has been advocating a highly individualized medicine from its beginnings.

Allopathic medicine has built much of its reputation on its success in the treatment of acute, life threatening diseases with antibiotics and surgery as prominent examples. Similarly homeopathy had many of its early great successes with the treatment of acute infections and epidemics.

Today, with higher life expectancy medicine faces the challenge of an increasing number of chronic diseases, which call for more and better individualization of therapeutic approaches.

**Objective**

In the case of homeopathy this translates into the challenge to be ever more precise and patient-specific in finding the optimal simillimum which mirrors the totality of the patient’s physical, mental and emotional uniqueness. Such an all-encompassing similliumum should ideally fit in all rubrics of a remedy – if it is well proven.

**Method**

In our practice we systematically tracked and compared different methods of identifying a patient’s simillimum with patient outcomes over 20 years.

**Conclusions**

Our findings show that lasting therapeutic results using a single remedy can be regularly achieved with a true simillimum remedy.

Patients for whom a truly perfect simillimum according to rubrics could be identified and who did not know, which remedy they were taking, could consistently identify the source of their remedy using associative interviewing techniques. This led to the unexpected discovery that the potential knowledge about the source of each patient’s simillimum lies within the patient and can be brought to light using adapted interviewing techniques.

## P258 Acceptance, satisfaction and cost during the pilot phase of an integrative pediatric program in a Swiss teaching hospital

### Tido von Schoen-Angerer^1,2^, Romy Schneider^1^, Livia Waeber^1^, Jan Vagedes^2,3^, Gregor Kaczala^1^, Cosette Pharisa^1^, Johannes Wildhaber^1,4^, Benedikt Huber^1^

#### ^*1*^*Department of Pediatrics, Fribourg Hospital HFR, Fribourg, 1708, Switzerland*; ^*2*^*Filderklinik, ARCIM Institute, Filderstadt, Germany;*^*3*^*University Hospital Tübingen, Dept of Pediatrics, Tübingen, Germany;*^*4*^*Department of Medicine, University of Fribourg, Fribourg, Switzerland*

##### **Correspondence:** Tido von Schoen-Angerer

**Question**

For the pilot phase of an integrative pediatric program, we defined inpatient treatment algorithms for bronchiolitis, asthma and pneumonia, using medications and nursing techniques from anthroposophic medicine (AM). Parents could choose AM treatment as add-on to conventional care. We aimed to evaluate the 18-month pilot phase.

**Methods**

Parents of AM users were asked at discharge to complete the Client Satisfaction Questionnaire (CSQ-8) and a questionnaire on experience with the AM treatment; patient charts were analyzed; and economic data for staff training, medications and insurance reimbursements were collected.

**Results**

A total of 351 children with bronchiolitis, asthma and pneumonia were hospitalized. Of these, 137 children (39%) received AM treatment, with use increasing over time. 52 parents completed the questionnaire, 27 (54%) had never used complementary medicine for their child. Mean CSQ-8 score was 29.77 (95% CI 29.04 to 30.5) which is high in literature comparison. 96% of parents were mostly or very satisfied with AM; 96% considered AM as somewhat or very helpful for their child; 94% considered they learnt AM care skills to better care for their child in the future; 87% thought they received sufficient information about AM. Cost for staff training and medications was nearly covered by AM related insurance reimbursements; no additional staff positions were created.

**Conclusions**

Introduction of complementary treatments as part of an integrative concept in a Swiss pediatric hospital department was well accepted and led to high parent satisfaction. Cost was compensated by additional insurance reimbursements.

## P259 Psychosomatic disorders – integrated approach to pathogenesis, treatment and care. New opportunities for management of chronic diseases based on synergetic methodology

### Pavel Sidorov, Evgeniya Sovershaeva

#### *Mental Health, Northern State Medical University, Arkhangelsk, Russian Federation*

##### **Correspondence:** Pavel Sidorov (pavelsidorov13@gmail.com)

**Question**

A mental pandemy of psychosomatic disorders (PD) is consistently developing and requires new effective approaches to prophylaxis, early diagnosis, treatment and care.

**Method/theory**

We have developed a synergetic bio-psycho-socio-spiritual concept of PD that is represented by a four-dimensional model consisting of vectors of somato- and psycho-, socio- and animogenesis. Animogenesis is a term that integrates understanding of soul and spirit and represents the central fourth part of the proposed ontogenetic model. The vectors are interrelated and determine transit zones with a central part containing conscience.

**Results**

We propose a synergetic concept for PD development with identified there prenosological stages: predisposition - a psychosomatogenic family, latent - psychovegetative diathesis, initial - a functional disorder and three nosological stages: full-scale clinical picture, chronic phase and outcomes. Based on the concept we have developed a multidisciplinary program of care for patients with PD, which includes four sections: medical, psychological, spiritual-moral and social. Preventive measures are implemented in the first three prenosological stages, treatment and rehabilitation - in the three nosological stages. The protocol is realized by a team composed of a general practitioner, psychotherapist, clinical psychologist, social worker and bioethics specialist. The model presupposes a multidisciplinary and integral approach to complex psychosomatic cause-effect relationships in the course of the disease. The proposed methodology makes it possible to formulate clinical, psychological, social and moral diagnoses resulting in a synergetic functional diagnosis.

**Conclusions**

Synergetic concept of the disease and a multidisciplinary program of care enable to establish personalized protocols for patients with PD, improve management and control the progression of the disease.

## P260 *Bryophyllum pinnatum* use in tocolysis in a conventional setting: a retrospective analysis from obstetric clinical practice

### Ana P Simões-Wüst^1^, Anna Nietlispach^1^, Mónica Mennet^2^, Martin Schnelle^2^, Ursula von Mandach^1^

#### ^*1*^*Obstetrics, University Hospital Zurich, Zurich, 8091, Switzerland*; ^*2*^*Weleda AG, Arlesheim, 4144, Switzerland*

##### **Correspondence:** Ana P Simões-Wüst (anapaula.simoes-wuest@usz.ch)

Since pre-term contractions are highly correlated with preterm delivery, the most common cause of neonatal mortality and morbidity, their inhibition by tocolysis is crucial. Several experimental data and a matched-pairs retrospective study revealed the potential of *Bryophyllum pinnatum* (BP), a herbal medication used as a tocolytic agent in anthroposophic medicine. In the last decade, treatment with BP has become more often at the University Hospital Zurich. Here we have analysed retrospectively the data from the clinical practice.

Well-documented female patients aged above 18 years, with a single pregnancy, undergoing tocolysis between 01.01.2009 and 31.12.2014 were included in the present analysis (*n* = 216). Data was extracted from the hospital and department databases, completed when necessary by consulting the clinical history, encrypted and analysed using IBM® SPSS® 22.

Patients were 33 ± 5.2 years old, had a gestational age of 29 weeks at admission and received state-of-the-art tocolytic treatment. BP was prescribed to 134 of the 216 patients (62%) as an add-on therapy. Time to delivery was longer among patients treated with BP than among the remaining ones (16 ± 19.7 vs. 9 ± 13.0 days, *p* = 0.011), in spite of comparable age of the mother, gestational age and cervical length at admission.

Our data suggests that BP is useful as an add-on tocolytic treatment. Further investigations on the clinical efficacy of BP as a treatment for pre-term contractions are warranted.

## P261 Reinforcing healthy*-qi* therapy (spleen-invigorating and kidney-tonifying therapy) in the treatment of postoperative relapse of colorectal cancer: a cohort study

### Xia Wang (350386855@qq.com)

#### *Xiyuan Hospital of China Academy of Chinese Medical Sciences, Oncology Department, Beijing, China*

Colorectal cancer has been recognized as a tumor of high incidence worldwide, radical resection in combination with radiotherapy or chemotherapy is commonly used in clinical treatment, while postoperative metastasis and relapse are the leading causes of death after radical resection, especially hepatic metastases. The key and difficult points of prevention and treatment are still the postoperative metastasis and relapse. Traditional Chinese medicine (TCM) plays a very important role in prevention and treatment of colorectal cancer. Preliminary studies found that TCM and western medicine combination was of high clinical value in reducing the postoperative metastasis and relapse of stage II and III colorectal cancer. The protocol established in this paper aims to further evaluate the effect of Chinese medicine in prevention and treatment of colorectal cancer after radical resection, and analyze the demographics of the patients who can benefit from the treatment and those who can’t, as well as discuss the possible mechanism of action. This study adopts the method of multi center prospective cohort study,We enrolled patients with stage II or III colorectal cancer, and screened 200 patients within 6 months after resection, and they were, according to their own will, given or not given TCM decoction (once daily) based on routine western medicine treatment. Then we followed up these patients up to 3 years, we are going to have a qualitative study of the subjects with relapse and metastasis after surgery for stage II and III colorectal cancer.

## P262 Efficacy and safety of Sa-am acupuncture on primary dysmenorrhea: study protocol for a randomized controlled trial

### Hye L Woo, Jin M Lee

#### *Department of Korean Gynecology, Kyung Hee University Hospital at Gangdong, Seoul, 05278, South Korea*

##### **Correspondence:** Hye L Woo (woohr74@naver.com)

**Background**

Primary dysmenorrhea is a common gynecological problem interrupting daily life. Some evidence supports the efficacy of acupuncture on primary dysmenorrhea, but one recent systematic review showed that high-quality clinical trials are insufficient. Especially, well-designed clinical studies of Koran traditional Sa-am acupuncture on primary dysmenorrhea are almost absent despite of its frequent application in clinical fields. Purpose: This study aims to assess the efficacy and safety of Sa-am acupuncture on primary dysmenorrhea.

**Methods/Design**

This trial is designed as a multi-center, randomized, double-blind, placebo-controlled trial. 100 Korean nulliparous women aged from 16 to 40 years with primary dysmenorrhea and regular menstruation will be recruited, and randomly allocated into true or sham acupuncture group. Acupoints used in both groups are Zulinqi (GB41), Houxi (SI3), Zutonggu (BL66), and Qiangu (SI2) on right side using "small intestine excess method". Both groups will receive treatment twice a week for 3 menstrual cycles and have additional 3 cycles without treatment for follow-up. The primary outcome is subjective pain severity measured by Visual Analogue Scale. The secondary outcomes are pain characteristics, quality of life measured by Short Form McGill Pain Questionnaire, Retrospective Symptom Scales, Short-form 36 health survey, and serum prostaglandin level. The safety of acupuncture will be assessed at every visit.

**Discussion**

We expect that Sa-am acupuncture could help patients who are unsatisfied and have side effects with conventional medications. This trial will assess the efficacy and safety of Sa-am acupuncture on primary dysmenorrhea and aims to provide reliable clinical evidence in treating primary dysmenorrhea.

## P263 The effects of pre-period acupuncture with heat-tonifying manipulation in dysmenorrhea patient

### Yuhao Wu^1^, Yumin Cho^2^

#### ^*1*^*Chengdu University of Traditional Chinese Medicine, School of Acupuncture, Chengdu, China;*^*2*^*ULCA, David Geffen School of Medicine, Center for East West Medicine, Los Angeles, CA, United States*

##### **Correspondence:** Yumin Cho (cho.yumin@gmail.com)

Endometriosis and adenomyosis are mainly characterized by progressively aggravated dysmeorrhea. In addition, adenomyosis is often accompanied by delayed period and menorrhagia. From the perspective of traditional Chinese medicine, secondary dysmenorrhea is attributed to stasis in thoroughfare (Chong Mai) and conception (Ren Mai) vessels. Heat-tonifying manipulation is a method to remove the stasis by producing the warmth in local area and promoting the needling sensation travelling along channels or spreading to nearby area. The purpose of the present study is to observe the effects of pre-period acupuncture with heat-tonifying manipulation on CV4, SP6, BL17, BL 32, and Ashi acupoints in dysmenorrhea patient. Three patients aged 29, 34 and 35, have been diagnosed as dysmenorrhea for more than 5 years with endometriosis and/or adenomyosis. All the patients had been treated by acupuncture with heat-tonifying manipulation and needles should be retained for 20 minutes for 4-7 times, 3 times a week, before each period for three menstrual cycles. As the results, after the pre-period acupuncture with heat-tonifying manipulation, all the patients have remarkably relived pain in pelvis and lower back during the menstrual period. One patient shows less menstrual blood volume and another one shows the menstruation that is more regular. In conclusion, puncturing CV4, SP6, BL17, BL32 and Ashi acupoints with heat-tonifying manipulation can remarkably alleviate pain and simultaneously relieve menorrhagia. The frequent treatment just before each period is more effective and the treatment requires retaining the needles for longer time.

## P264 Development of a mobile application in conjunction with a web service for personal health records regarding atopic dermatitis

### Younghee Yun^1^, Hyunho Kim^2^, Wonmo Jung^3^, Bo-Hyung Jang^4^

#### ^*1*^*Department of Korean Medicine Dermatology, Kyung Hee University Hospital at Gangdong, Seoul, 134-806, South Korea*; ^*2*^*Department of Biofunctional Medicine & Diagnostics, Kyung Hee University Korean Medicine Hospital, Seoul, South Korea;*^*3*^*Department of Science in Korean Medicine, Graduate School, Kyung Hee University, Seoul, South Korea;*^*4*^*Department of Preventive Medicine, Graduate School, Kyung Hee University, Seoul, South Korea*

##### **Correspondence:** Bo-Hyung Jang (jbh8284@gmail.com)

**Purpose**

Although a myriad of mobile applications that provide health or medical information have been developed for various diseases including atopic dermatitis (AD), most of them are not used by physicians in clinics. Usually they are used by patients themselves only to record their symptoms or get information about diseases. The purpose of this study was to develop a smart system which can record and retrieve medical symptoms and clinical information generated in daily life in order to connect physicians and patients and to enhance the quality and the efficiency of medical care.

**Methods**

Task force team consisting of three physicians and two developers has developed the system. Patient centered informatics tool for AD was developed by implementing a mobile application to collect daily information related to AD from patients and a web service to assist physicians for retrieving and integrating symptom information. User interface of mobile application was designed under consideration of convenient daily input. On web service for physicians, visualization and tabulation of the time-series recorded symptoms were designed based on the practical need of physicians. The developed system has been evaluated from July 2015 to August in clinical situation.

**Results**

The smart phone application, Atopy Mobile Note, has been registered in Google Play Store from March 2016. Patients can record AD score, episodic events of their illness, food diary, treatment and management diary using their mobile devices. Recorded information is saved in database of central server immediately. Physicians can retrieve a sequence of records in the clinic using web service program.

**Conclusion**

We have developed an mPHR application for patients and corresponding web service for physicians. To take advantage of this system in health care will require more research to find out how much it actually more beneficial to patients and/or physicians.

Keywords: Personal Health Records; Electronic Health Records; Atopic Dermatitis, Application.

## P265 Development of an integrated Chinese-Western medicine (ICWM) model - practical expertience of ICWM pilot program in Hong Kong public hospitals for inpatients

### Eric Ziea, Henny Hui, Mia Li, Dora Tsui, Christine Lam, Joyce Hsieh, Edith Chan

#### Chinese Medicine Department, Hospital Authority, Hong Kong, Hong Kong

##### **Correspondence:** Christine Lam (lsm572@ha.org.hk)

**Purpose**

Coventional medicine has been the mainstream in the public healthcare services in Hong Kong, and Chinese Medicine (CM) was privately practiced as a traditional alternative. In recent years the Hong Kong Government has set out the policy direction to promote integrated Chinese and Western (ICWM) medicine care model, and to explore the optimal model for the development of the first CM hospital in Hong Kong.

**Method**

In order to test out the logistical and care model for providing ICWM care to inpatients, HA set up a task force involving multidisciplinery workforces to formulate operational and clinical frameworks, and collaborated with CMCTRs to implement a small-scale pilot in selected public hospitals to provide ICWM treatment in three disease areas, including cancer palliative care, stroke and acute low back pain.

**Result**

Numerous issues were identified when introducing the alternative medicine component into a conventional medicine mainstreamed patient care setting. E.g. professional knowledge gap, integration of medical record, herb-drug interaction and risk management, clinical accountability, legal liability, role of nursing etc.To overcome these conflicts, various teams and working groups were formed under the task force to formulate training, mechanisms and workflows. With the efforts, a set of clinical protocols and guidance was formulated, which contributed to a fesiable model for the Phase I program inplementation in September 2014.

**Conclusion**

The practical experience accumulated in this pilot programme would guide the development of an optimal ICWM in-patient model and the future of ICWM services in the public healthcare system of Hong Kong.

## EDUCATION

### P266 Taking the first step: assessing patients interest and education needs related to complementary and integrative healthcare

#### Lynda Balneaves^1^, Sandra Burnside^2^, Ethel Doyle^2^, Shelley Dorazio^2^, Pak K Chan^3^

##### ^1^ College of Nursing, University of Manitoba, Winnipeg, R3T 2 N2, Canada; ^*2*^*The Scarborough Hospital, Toronto, Canada;*^*3*^*Leslie Dan Faculty of Pharmacy, Toronto, Canada*

###### **Correspondence:** Lynda Balneaves (lynda.balneaves@umanitoba.ca)

**Introduction**

Prior to developing an integrative healthcare program, a needs assessment was conducted with patients at a community hospital. The purpose was to determine patients use of CIH, their interest in receiving CIH services at the hospital, and their education and support needs.

**Methods**

The survey, available in English, Chinese and Tamil, was distributed to patients over a one-week period. The survey captured patients CIH use, interactions regarding CIH with healthcare providers (HCPs), and preferences regarding CIH education and service provision at the hospital.

**Findings**

554 surveys were completed, with the majority of patients being female (57.9%) and English speaking (61.6%). Currently, 25% of patients were seeing a CIH professional and 53.6% were using a CIH therapy. 67.1% expressed interest in receiving CIH services at the hospital, with massage, chiropractic care, and acupuncture being most popular. However, only 1 in 5 patients expressed a willingness to pay for CIH services. Patients reported limited dialogue with HCPs about CIH, with less than 20% discussing CIH with an HCP or receiving information or decision support. However, 57.0% were interested in attending a CIH education program, if it existed at the hospital.

**Conclusion**

Despite the high prevalence of CIH use, patients received limited education and support about CIH from HCPs. Innovative education and decision support services are required that will provide patients with the information required to make informed choices about CIH. The development of an integrative health centre will offer patients a safe, evidence-based, and comprehensive environment in which to receive CIH.

### P267 Incorporation of Integrative Medicine education into undergraduate medical education – a longitudinal study

#### Anjali Bhagra

##### *Mayo Clinic, Rochester, MN, United States*

###### **Correspondence:** Anjali Bhagra (bhagra.anjali@mayo.edu)

**Purpose**

Integrative medicine (IM) combines complementary medical approaches into conventional medicine taking into account the whole person. We implemented a longitudinal IM short course curriculum within our medical school education. The purpose of this study was to evaluate the feasibility and effectiveness of the curriculum via knowledge and attitude surveys regarding IM in students.

**Methods**

A mandatory short IM curriculum across all years of medical school was created and taught by physician faculty members with expertise in integrative therapies and IM professionals. Students in two consecutive Mayo Medical school classes (class of 2015 and 2016) completed an optional survey testing their knowledge and attitudes regarding IM therapies and their personal health practices. Students completed the same survey during their first and third years of medical school. Paired data analysis was done and only students who filled out surveys at both time points were included in final analyses.

**Results**

17 out of 52 students of class of 2015 and 22 out of 52 students of class of 2016 completed both the surveys. Following the IM curriculum, students’ knowledge and comfort with following IM therapies improved significantly: biofeedback, mindfulness, and the use of St. Johns Wort. Students personal health practices improved by almost 27% including better sleep, exercise and stress management. There was reported decrease in stress compared to their entering year, and less alcohol use.

**Conclusions**

It is feasible to incorporate IM education into undergraduate medical education and this is associated with improvement in students knowledge in IM as well as personal health practices.

### P268 The development of an acupuncture simulation module on LR14 for acupuncture treatment of hepatic disorder

#### Po-Hsu Chen

##### *Chinese medicine, Changhua Christian Hospital, Changhua, 500, Taiwan*

LR14 is an important acupuncture point for acupuncture treatment of hepatic disorder in traditional Chinese medicine. However, pneumothorax could happen when LR14 was needled improperly. For patient safety, an acupuncture simulation model is needed. This study aims to develop an LR14 simulation model (SM14) for acupuncture training. SM14 includes two parts: a simulating rib cage and acupuncture detecting system. Silicon, gelatin and aluminum were used to make skin, muscle and rib of the simulating rib cage. A light sensor was imbedded at the bottom of the muscle layer to simulate LR14. An optical fiber was attached to an acupuncture needle and ready for insertion. The sensor was activated by laser beam emitted from the optical fiber which made up an acupuncture detecting system. After SM14 was set up, five users were asked to perform acupuncture on SM14 and report their commands. All users approved SM14 a good simulation training tools. SM14 as an acupuncture training tool is helpful for improving quality of acupuncture practice.

### P269 Promoting Integrative Chinese-Western Medicine care models through the development of an evidence-based electronic resource and training program: protocol of a mixed method study

#### Vincent CH Chung^1,2^, Justin CY Wu^2^, Zhi X Lin^2^, Wendy Wong^2^, Xin Y Wu^2^, Robin ST Ho^1^, Charlene HL Wong^3^, Lily Chan^2^, Eric TC Ziea^2^

##### ^1^ Jockey Club School of Public Health and Primary Care, The Chinese University of Hong Kong, Shatin, Hong Kong, Hong Kong; ^*2*^*The Chinese University of Hong Kong, Hong Kong Institute of Integrative Medicine, Hong Kong, Hong Kong;*^*3*^*Department of Medicine and Therapeutics, The Chinese University of Hong Kong, Shatin, Hong Kong, Hong Kong*

**Purpose**

Evidence from systematic reviews and randomized controlled trials facilitate development of integrative Chinese – western healthcare models in an evidence based manner. An open access website of critically appraised synopses plus educational outreach to healthcare professionals will be established to promote the uptake of evidence on effectiveness and safety. This study proposes to refine the writing format of synopses through usability testing; investigate the effectiveness of providing Evidence-based medicine (EBM) education for Chinese medicine practitioners (CMPs); and compare the effectiveness of disseminating clinical evidence synopses through Facebook versus WhatsApp on top of synopses website among CMPs.

**Methods**

There are three phases in this mixed method study. Firstly, a usability study of the synopsis will be conducted using iterative cycles of testing with CMPs. The System Usability Scale (SUS) will be used for usability testing of synopses. This helps refine the writing format of synopses which will be uploaded on the open access website. Secondly, a randomized controlled trial of education on EBM and related methodology to CMPs will be conducted. Lastly, targeted messaging of clinical evidence synopses with quality of evidence grading to CMPs will be performed over 10 weeks. A mixed model analysis of outcomes will be performed based on the intention-to-treat principle in this study.

**Expected results**

This will be the first study establishing bilingual website dedicated clinical evidence on Chinese and Integrative medicine; investigating the effectiveness of EBM education and target messaging of clinical evidence synopses among CMPs.

### P270 Enhancing chronic pain treatment through an interdisciplinary strategy: the Central Appalachia Inter-Professional Pain Education Collaborative (CAIPEC)

#### William Elder, Roberto Cardarelli

##### *University of Kentucky, Lexington, KY, United States*

###### **Correspondence:** William Elder (welder@uky.edu)

**Question**

Chronic pain is increasingly recognized as a public health problem. This is especially so in the rural Appalachian region of the United States. An interdisciplinary approach to provider education is needed to empower medical professionals to make deliberative changes, especially in opioid prescribing practices.

**Methods**

Enduring webcasts, regional inter-professional roundtable events, and state-level conference presentations addressed behavioral factors in pain, use of alternative treatments and new treatment guidelines. Effectiveness of these activities was based on pre- and post- measures, as well as comparison to a control population, on: provider’s intentions to change their care of chronic pain patients, confidence in meeting chronic pain patient’s needs, and knowledge of pain management guidelines.

**Results**

Over 1,000 participants accessed the educational activities. For live events, the largest groups reached included nurses (38.1%), nurse practitioners (31.2%) and physicians (22.1%). A majority of conference (58%) and roundtable (69%) participants stated that they intend to make a change in practice in one or more areas related to chronic pain patients and opioid use. Participants were more confident post-activity intheir ability to change practice and.there were significant changes in knowledge from live event as well as webcasts.

**Conclusions**

The approach and methodology to interdisciplinary professional chronic pain education was shown to impact learner’s knowledge and confidence, and holds potential for creating change in how opioid prescribing is managed.

### P271 The Simonton-Training – how cancer patients and their loved ones can support the effects of treatment by increasing their quality of life

#### Cornelia Kaspar (info@simonton.eu)

##### *Simonton Cancer Center, Ulm, 89077, Germany*

Dr. O. Carl Simonton, an internationally acclaimed oncologist pioneered the field of psychosocial oncology and is well known for the first systematic emotional intervention used in the treatment of cancer – a model of emotional support for the treatment of cancer patients which is considered to complement the medical treatment.

As a psycho-oncologist, Simonton Counsellor, Supervisor and Trainer I was one of Carl Simonton`s closest team members and got his authorization to continue his training concept in Germany, Switzerland, Italy, Belgium and the Netherlands. I would like to provide deeper insight into this unique therapeutic approach, which may be described as a cognitive-behavioral self-help therapy for cancer patients and their support persons.

The therapy directly addresses all spheres of human existence – emotional, cognitive, behavioral, social and spiritual – integrated in an internally consistent program, based on modern learning theory as well as ancient teachings. Our strategies include individual, group, family and social counseling as well as education in self-help skills. We focus on individual resources to support patients and their support team in gaining joy, increasing hope, trust and peace of mind with uncertainties in life. We work with guided imagination, meditation and mental tools for changing unhealthy beliefs. The clients are invited to reflect about the things that are helpful for them and to give priorities to their health. They learn how to increase awareness for their feelings and needs and get support to follow their own inner wisdom and realize changes in their life to live according to their own nature.

### P272 Training in anthroposophical medicine at the University of Strasbourg

#### Robert Kempenich^1^, Jacques Kopferschmitt^2^

##### ^*1*^*AREMA, Strasbourg, 67000, France*; ^*2*^*Unistra, Strasbourg, 67000, France*

The training in Anthroposophical Medicine at the University of Strasbourg (UNISTRA FRANCE), according to the concept of Integrative Medicine, proposes an expansion of conventional and academic medicine.

Anthroposophical medicine takes into account the global nature of the patient and provides additional means (genuine professional added-value treatments) that health professionals can adapt to each specific case.

In a large number of pathologies, Anthroposophical medicine helps greatly reduces adverse effects of treatments and minimize cost.

The course is intended for hospital doctors, general practitioners and specialists, pharmacists, dentists and midwives.

Annual teaching and training courses consist of 4 days of basic trainings and 3 or 4 trainings on 2 consecutive days.

**Examples of training courses**

4-day training course:

- Basic training in Anthroposophical medicine

2-day training courses:

- Autoimmune diseases
- Supportive care in oncology
- Anxio-depressive states
- Dermatology
- Rheumatology

**The aims of these courses are**

- To enable students to understand the diagnostic and the therapeutic approach of Anthroposophical medicine
- To discern technical and therapeutic indications of Anthroposophical medicine, which is perfectly complementary to conventional treatments
- To learn to sharpen prevention and act upstream of heavy treatments (what often allows to avoid the recourse to antibiotics or psychotropics).
- To learn how to accompany heavy treatments whenever necessary (supportive treatments).

These courses aim to respond to a need for a deeper qualitative professional approach for the benefit of patients.

This approach is complementary to all conventional therapies.

### P273 Attitudes towards Integrative Medicine among Medical Faculty of Osijek students

#### Zulj Marinko, Sebo Damir, Aleksandar Vcev

##### *Department of Integrative Medicine, Faculty of medicine Osijek, Osijek, 31000, Croatia*

###### **Correspondence:** Zulj Marinko (marinko.zulj@mefos.hr)

The aim of this study was to investigate attitudes towards integrative medicine among medical students in the foruth, fifth and sixth year at the Medical Faculty University Josip Juraj Strossmayer of Osijek.117 students have filled an anonymous questionnaire consisting of two parts. The first part included 6 general questions and the second part was composed of 14 claims relating to attitudes toward integrative medicine. The participants were asked to note their agreement or disagreement with the claims along the Likert scale. The highest number of students were undecided about most claims regarding integrative medicine. There was significant difference regarding the answer to question whether integrative medicine should be a part of regular procedures within basic health insurance, where the highest number of students who wish to work in clinical medical practice agreed partially with that claim (Chi-Square Test, *p* = 0,013). Results of our study suggest that there is a lack of knowledge about integrative medicine among our students. This fact emphasises the need for introduction of the new course where students will have an opportunity to learn about integrative medicine and accept it as a new approach in medicine that puts the patient at the centre, being both preventive and personalized. Thus our initiation of a new course Integrative medicine as a part of curriculum for sixth year in 2016/2017 academic year has been proven rightful and justified.

### P274 "Dialogue about Spirituality and Health": an educational transdisciplinary action of the practice of spirituality in order to promote quality of life and integral health

#### Ricardo Monezi^1^, Eny Márcia Ruggerini^2^, Ivna M. Fuchigami^3^

##### ^*1*^*Campus Avançado de Extensão Santo Amaro, UNIFESP, São Paulo, 04753060, Brazil*; ^*2*^*Setor Espiritualidade e Saúde, Fundação Mokiti Okada, São Paulo, 04015-050, Brazil;*^*3*^*Religious Studies, Methodist University of São Paulo, São Bernardo do Campo, 09641-000, Brazil*

###### **Correspondence:** Ricardo Monezi (ricardomonezi@gmail.com)

**Question**

Report the implementation and development of an experiential transdisciplinary action of spirituality practice learning in order to promote quality of life and integral health named "Dialogue about spirituality and health."

**Method**

Case report

**Results**

Since February 2004, the Mokichi Okada Foundation (MOF), a non-profit legal entity of private law, considered as Federal Public Interest, located in the city of São Paulo, Brazil, has a health department that has been developing an education program concerning the practice of spirituality for health professionals. So far, it has provided knowledge to more than 500 people. Despite the relative success of this action, it was concentrated and limited to a specific audience: professionals with higher education, graduated in several fields of health. In order to meet the interests of a wider audience with no specific training, the health department has developed and implemented in early 2014, a space of spiritualty practice learning in order to promote quality of life and integral health entitled "Dialogue about spirituality and health". This transdisciplinary educational activity takes place on a monthly basis at the headquarters of MOF. With a two-hour duration, the "Dialogue" comprises two stages: the first, lasting thirty minutes, consists of a theoretical explanation based on scientific literature searched in database such as PubMed, SCIELO or WEB OF SCIENCE. During the second step, a dialogue among participants is conducted and encouraged, in order to facilitate knowledge and experience exchange. Until October 2016, 24 events have been held with an attendance of about over 1,000 people. Since the october of year 2015, events have also been broadcast through the Internet, in real time, reaching viewers in several Brazilian states and even those from other countries like USA, Canada, Japan and Germany.

**Conclusions**

With an audience of different age ranges and backgrounds, from different cultures and religious rites, the "Dialogue about Spirituality and Health" has strengthened itself as a democratic experience of teaching and discussion on the practice of spirituality in order to promote quality of life and integral health.

### P275 The Cerrado as a curing cradle: medicinal plants from the perspective of healer´s knowledge and tradition in the campus of Federal University of São Carlos (UFSCar), Brazil

#### Ana C Moreno Mazini^1^, Ricardo Monezi^2^, Maria Waldenez Oliveira^1^

##### ^1^ Departamento de Ciências Biológicas, Universidade Federal de São Carlos, São Carlos, 13565-905, Brazil; ^*2*^*UNIFESP, São Paulo, 04753060, Brazil*

###### **Correspondence:** Ana C Moreno Mazini (anacarolinamazini@gmail.com)

**Question**

Brazilian Cerrado is one of the world's hotspots, considered the tropical savanna with the greatest diversity of the planet. Your plant heterogeneity houses approximately 7000 plant species, largely unknown. Despite this, 55% of your area has already been deforested. In São Paulo State, there´s only 0.8 of vegetation, responsible for the Guarani aquifer recharge. In the municipality of San Carlos, the domain is also fairly fragmented, with an important area is located on the campus of the Federal University of São Carlos (UFSCar). Because of the heterogeneity and the specific morphological features, the Cerrado flora presents great medicinal potential, widely disseminated in popular culture.

**Methods**

In order to get to know medicinal plants of the Cerrado of UFSCar on the optics of the popular healers knowledge in the region, a tour guided in the area and a semi-structured interview (discussing, among other issues, aspects of culture and conservation) in order to contribute to the enhancement of knowledge and local vegetation at a time where the National Integrative Practices Policy and the movement of Popular Education (intensely connected to the traditions and herbs) gain space in the city.

**Results**

The healers easily identified a significant number of medicinal plants at the place of study, even without prior knowledge of the area. This reveals a precious wisdom about herbs, which extrapolates the herbal issues itself and expands cultural and environmental aspects.

**Conclusions**

The results obtained revealed the deep knowledge of the healers and the importance of conserving the Cerrado area of UFSCar as a source of studies on medicinal plants in the State and in the Country. The appreciation of the popular regional knowledge and local flora are aspects essential to the strengthening of Popular Education in Health and Integrative Medicine movements.

### P276 Extraordinary vessels, body fluids and body posture -subtle governors of autonomous nervous system signaling

#### Petar Papuga

##### Daofa, Komenda, 1218, Slovenia

Explaining the priorities of the field of Integrative medicine could be thoroughly enriched through the updated understanding of classical theories in textbooks of Traditional Chinese Medicine (TCM). There are strengths and weaknesses in the methods that could provide a vehicle for the transfer of ancient expert knowledge to the modern research, which is essential for the benefit and quality improvement of the integration process.

Scientific studies of TCM are sustaining such evidence through the follow up of neurotransmitters secretion in central and autonomous nervous system (ANS). Maintenance of the body posture is very useful clinical approach in various kinds of patients, resulting in removal of blood and extracellular fluid congestion and improved feed-back loop of autonomic nerves signaling. Application of electrical stimuli and cupping therapy on viscerotomes shows ample clinical improvement of well being among cancer patients. Viscerotomes are important feed - back loop regions to observe, follow and restore functioning of their pertaining organs. ANS signaling, through the paravertebral ganglia and periaqueductal grey matter reflects congestions of extracellular fluids as well as various visceral disturbances. Those changes are common cause of increased tonus in paravertebral muscles, which impedes stagnation of extracellular fluid.Main purpose of poster presentation is to explain the correlation between Extraordinary, Small Intestine and Urinary Bladder vessels, body posture, body fluids and diet for the restoration of ANS disturbances. Degeneration of vertebral cartilage, fascia or accompanied adjacent connective tissue can slowly compress the vegetative nerves (which is, contrary to compression of sensory nerves, painless), and finally disturb the autonomous regulation of visceral signaling to the brain stem nuclei, especially solitary tract nuclei.

Lifestyle changes, exercises and individualized dietary recommendations are to be explained in addition to ongoing treatment strategies. Balancing ANS is feasible approach as a support for various health disturbances, many health and disease conditions.Ample clinical evidence shows that we should pay more attention and higher priority to this aspect of TCM in described clinical treatment. In order to recognize, treat and improve methodology of clinical follow up or in prospective studies in the field of Integrative Medicine.

### P277 Implementing patient reported outcome measures into a student clinic environment

#### Janet Schloss, Amie Steel

##### Office of Research, Endeavour College of Natural Health, Fortitude Valley, 4006, Australia

###### **Correspondence:** Janet Schloss (janet.schloss@uqconnect.edu.au)

**Purpose**

Patient reported outcome measures (PROMs) are validated questionnaires which collects data from a patients perspective about their health status or treatment. PROMs can be useful to both health professionals and researchers within complementary medicine (CM) to collect this information in a systematic and consistent manner. The use of PROMS can support evidence-based clinical decision making at a clinical level as well as facilitate clinic-based research. However, the integration of PROMs into clinical practice requires CM clinicians to be sufficiently exposed and appropriately trained in their use.

**Methods/Results**

The integration of the use of PROMs into a student clinic within a CM educational institution has been undertaken through a process designed to ensure students, supervisors, researchers and the institution gain maximum value from their inclusion. The outline of the process undertaken to implement PROMs within six different clinical sites and four clinical disciplines across Australia for the Endeavour College of Natural Health will be explained. The pros and cons of the implementation and lessons learnt will be examined.

**Conclusion**

PROMs are a valuable tool which can be implemented into student clinics in a CM education institution. By utilising these tools, students and supervisors can gather important information pertaining to the outcome of the health intervention used for patients. Moreover, as PROMs are validated instruments they afford the student the ability to publish case studies or case series in peer reviewed journals, thereby supporting research evidence and research capacity within CM. Certain PROMs, such as the Euroqol5D, can also assist in economic cost-benefit analysis. PROMs are important clinical tools for health practitioners and exposing student to PROMs during their clinical training assists them in understanding how to use these tools, why they are important, and how to incorporate them in their clinical setting post-graduation.

### P278 The importance of developing consciousness about health and integrative medicine in education process of professional massotherapists, in Porto Alegre City, Brazil

#### Márcia da Silva Jacobsen^1^, Ricardo Monezi^2^, Miranda Rodrigo Jacobsen^3^, Maria T. Mangini^1^

##### ^1^ Curso Técnico em Massoterapia, Escola de Educação Profissional Cecília Meireles – SEG, Porto Alegre, Brazil; ^*2*^*UNIFESP, São Paulo, 04753060, Brazil;*^*3*^*Fisioterapia, Centro Universitário Metodista-IPA, Porto Alegre, 90420-004, Brazil*

**Question**

To present the importance of developing consciousness about "health and integrative medicine" in education process of professional massotherapists in Porto Alegre City, Brazil.

**Method**

Case report.

**Results**

Rio Grande do Sul is one of the 27 states of Brazil. Located in the south of the country, it has an area of over 280,000 km2 and a population of over 11 million residents, and the city of Porto Alegre is its capital. In this context was raised the *"Sistema Educacional Gaucho"* (SEG), with the proposed construction of a solid education system, with a standard of quality focused on education, which calls for educational work structure facing a commitment to awaken the student the sense of collective, social, preparing man for the "think - act - be" inserting and engaging in turn, interact actively in society. Encourages students to live effectively in society, not being mere spectators, but talented actors and excellence in the performance of their actions, leadership, decision making in collective processes. Its courses are organized considering the competences required by the labor market, social, economic and political, so that the student at the end of their qualification and training has a systemic and integrative view. Among the courses offered by SEG is technical in massage therapy, which aims to train professionals for the development of massage techniques that can work with esthetic, therapeutic, manual lymphatic drainage, shiatsu and sports massage recovery, and other integrative techniques like argil therapy and auriculoacupuncture. In its formation there are disciplines that directly address the issue of health and integrative medicine as a discipline " Complementary Therapies " and " Personal relationships ", discussing the importance of the development of this way of thinking as a new care paradigm to the human being in his various dimensions and transdisciplinary way. Complementing the theory, students undertake consultations at public events with several other integrative therapies in addition to massage, such as reflexology, moxibustion, shiatsu therapy and aromatherapy. In the past two years (2015 and 2016) were met over a thousand people with these techniques.

**Conclusions**

From theory to practice, which is the service to the population, students have the chance to experience the importance of integrative medicine in the development of whole health; the experience of direct assistance to human underscores the consciousness that the therapeutic of integrative medicine go far beyond the technical skills, particularly understanding the essential humanity of the true care.

### P279 Guideline for osteopathic contribution in pediatrics

#### Gianfranco Trapani^1^, Tiziana Di Giampietro^2^, Luisella Zanino^1^, Luigi Ciullo^3^, Diego Lanaro^3^, Francesco Cerritelli^4^, Francesco Macrì^2^

##### ^1^ SMB ITALIA, Sanremo, 18038, Italy; ^*2*^*SIOMI, Integrative Medicine School, Firenze, Italy;*^*3*^*Istituto Europeo di Medicina Osteopatica, Osteopathy School, Genova, Italy;*^*4*^*COME Collaboration ONLUS, Fondazione per la Ricerca in Ambito Osteopatico, Vasto, Italy*

###### **Correspondence:** Tiziana Di Giampietro (tiziana.digiampietro@gmail.com)

This work is the result of an interdisciplinary cooperation between pediatricians and osteopaths. It aims is to promote knowledge about osteopathic techniques and their applicability in pediatrics, as a complementary and adjuvant tool. Osteopathy is a non-invasive manual therapy with no iatrogenic effects, ranked by WHO among the traditional medicines (WHO 2016) which can be associated with other therapies (pharmacological, conventional, herbal and/or complementary medicine).

The osteopathic professionals who have concluded a five-year training course and subsequent specialization courses in pediatrics can perform a bodily evaluation of patients, identifying somatic dysfunctions and normalizing them by applying an osteopathic manipulative treatment (OMT) – however, osteopaths never make a diagnosis, nor do they give medical therapeutic advice, prescribe drugs or recommend medical exams.

Not only is the Italian legislature discussing a regulation for osteopathy, but a growing number of studies demonstrate its clinical efficacy in areas as diverse as colics, gastroesophageal reflux, scoliosis, delays of neuromotor development, asthma, ear infections, neonatology, plagiocephaly and autism. Further research focusing on the phenomena of interoception and sensitization, as well as somatovisceral and viscerosomatic reflexes, suggests that OMT can activate anti-inflammatory and hyper-parasympathetic effects (D'Alessandro et al. 2016).

These guidelines are meant to lay the foundations for a closer collaboration between pediatricians and osteopaths, also providing a brief description of osteopathic medicine, some references of the most important literature and a glossary of terms.

Keywords: osteopathy, OMT, pediatrics, complementary medicines, therapeutic synergy

### P280 History and teaching acupuncture experience at FMUSP

#### Andre Tsai^1^, Chin Lin^2^, Tu-Hsing Wu^2^, Eduardo D'Alessandro^2^

##### ^1^ Acupuncture Centre of IOTHCFMSUP, São Paulo, 05410001, Brazil; ^*2*^*Acupuncture Centre of Orthopaedic and Traumatology Institute of Hospital das Clinicas, Medical School, São Paulo University, São Paulo, Brazil*

###### **Correspondence:** Andre Tsai (tsai.andre@gmail.com)

Acupuncture is part of Traditional Chinese Medicine (TCM) practice that has become very popular in Brazil in last 50 years. Due to many scientific researches that explain these healing technique mechanisms of action to treat several clinical conditions, Brazilian Federal Council of Medicine (CFM) recognized Acupuncture as a Medical Specialty since 1995.

In this year the authors started teaching Acupuncture at Orthopaedic and Traumatology Institute of Hospital das Clínicas of São Paulo University only for already graduated physicians. In 2000, we started teaching under graduated students from 3^rd^ and 4^th^ grade at The University of São Paulo Medical School.

In 2007,the Medical Residency Program in Acupuncture started with 2 years of duration and 2880 hours per year including exchange training program between Chang Gung Memorial Hospital and Taipei City Hospital, both from Taiwan.

In 2014 Brazilian Sanitary and Vigilance Agency (ANVISA) allowed the use of Chinese Herbs that contribute forward to more integrative Medicine. In the same way, we also created a Chinese Herbs Course for Medical Acupuncturists.

During 20 years, more than 1,000 students of Medical Course and more than 500 physicians learned Acupuncture and TCM theories that helped them to make a better approach combining Western Medicine and TCM treatments in their clinical decisions.

### P281 General practitioner views and understanding of prostate cancer screening and management with active surveillance

#### Sam Watts (s.watts@soton.ac.uk)

##### Primary Care, University of Southampton, Southhampton, SO16 5ST, United Kingdom

**Question**

Prostate cancer is the most common male cancer in the UK. There is presently no national screening programme for prostate cancer in the UK although asymptomatic patients are increasingly being diagnosed earlier following Prostate Specific Antigen (PSA) testing. Recent developments in the management of localised prostate cancer include the increasing use of active surveillance (AS). With both PSA testing and AS being increasingly managed by General Practitioners (GPs), prostate cancer is an important health problem in primary care. The aim of this study was to 1) better understand GPs views on PSA screening and to establish what additional support could be given to GPs in order to help manage asymptomatic men requesting PSA tests and 2) to investigate GPs understanding of the use of AS in the management of localised prostate cancer and to establish any additional resources that could be provided to better manage these patients.

**Methods**

Twenty telephone interviews were conducted with practising GPs recruited from the primary care research network in the UK. Interviews were semi-structured but an iterative approach was taken, allowing emerging areas to be explored. Interviews were digitally recorded and transcribed verbatim, after which transcripts were analysed using a thematic approach.

**Results**

There were two overarching themes – 1) PSA testing in asymptomatic men and 2) the management of localised prostate cancer with AS. PSA screening in asymptomatic men was not advised by GPs with many giving the potential need for further invasive investigations as a main disadvantage. Main areas of interest for AS were the need for clear guidelines on the management of AS in primary care, confusion over responsibility for different aspects of AS and the importance of communication between primary and secondary care.

**Conclusion**

With the increasing incidence of prostate cancer it is important to appreciate the role of primary care in managing these patients and to understand any areas that could be improved.

### P282 Social network and healthcare seeking behavior in urban communities: A pilot study on using Traditional Chinese Medicine

#### Ying Zhang^1^, Xufang Wu^2^, Xun Li^1^, Yutong Fei^1^, Jianping Liu^1^

##### ^1^ Center for Evidence Based Chinese Medicine, Beijing University of Chinese Medicine, Beijing, China; ^*2*^*Changying Community Health Service Center, Beijing, China*

###### **Correspondence:** Ying Zhang (novelzhang@sina.com)

**Question**

It is widely documented that health care usage is associated with the characteristics of social networks involvement. However, the theoretical hypothesis had seldom been empirically confirmed in epidemiology studies. This study was designed to identify individual social network types among residents of urban communities of Beijing, and examine the relationship between social network type and health seeking behaviors, mainly focused on the usage of Traditional Chinese Medicine (TCM).

**Methods**

Our study was approved by the ethics committee of Beijing University of Chinese Medicine before initiation. Therefore, A pilot study was carried out from June to August in 2016. Samples were selected from Changying neighborhood committee in Chaoyang district of Beijing, China. Data capture in questionnaire included demographic information, social and economic status, healthcare seeking behaviors, and characteristics of individual social networks. According to the theory of Blau and Duncan, we classified social networks into two types: inborn and afterwards networks. Influenced by the strong-weak tie theory of Granovetter, we also explored the effects of strong and weak ties on health seeking behaviors.

**Results**

Totally, 38 participants completed the questionnaire. 22 of them are male. The average age was 42 ± 5. The average size of social networks which had been considered to be related to healthcare behaviors was 6. Social network type was significantly associated with several health seeking behaviors, after controlling for background characteristics. We also found that elder residents would more likely to use inborn social networks when seeding healthcare than younger people. But, they were more likely to believe the advices from weak ties when making decisions on medical affairs.

**Conclusions**

This pilot study highlights the importance of social networks on healthcare seeking procedure. Family and peer education based on communities were still needed to promote individuals knowledge, attitudes and behaviors on TCM.

### P283 The analysis of communicate apprehension in students of TCM speciality and affecting factors

#### Nanqi Zhao^1^, Liyan Jia^1^, Xiaoyi Yan^1^, Fei Zhen^2^, Zhaolan Liu^1^, Jianping Liu^1^

##### ^1^ Evidence-based medicine center, Beijing University of Chinese Medicine, Beijing, China ^2^ Dongfang Hospital, Beijing University of Chinese Medicine, Beijing, China

###### **Correspondence:** Zhaolan Liu (lzl1019@163.com)

**Objective**

To investigate the present situation of traditional Chinese medicine college students in communication apprehension, we make a survey and then analyze the relative factors between different communication apprehension grades.

**Methods**

We designed a cross-sectional study, and inquiried the student from five classes in Beijing University of Chinese Medicine. We having collected the basic information, performance in college, and communication comprehension degrees, we designed questionnaire including gender, whether the only-child, growth environment, average grades, credits grades. Then we used Personal Report of Communication Apprehension (PRCA – 24) to evaluate the degrees of communication apprehension. We distributed PRCA - 24 scale and self-designed questionnaires to students. We applied EpiData3.1 software to establish database and data entry control procedures, and used SPSS 22.0 software for statistical analysis.

**Result**

We distributed 184 questionnaires, and recived 163 valid questionnaires. Effective response rate is 88.6%. The achievement and credit grade of communication apprehension student in different levels have no statistical difference. Communicate apprehension level has not different distribution in gender, whether the only-child,and length of schooling. But it is related to students' growing environment.

**Conclusion**

The survey shows that 80.37% of students belonging to medium and high degree in communication apprehension. Doctors should be good at communication, therefore it is necessary that make an anylysis on students majoring in Traditional Chinese Medicine for communication apprehension regularly. So that it's easy for educators to grasp the situation of students' communication apprehension and take corresponding measures to reduce the adverse effects led by communication apprehension.

## TRADITIONAL HEALING SYSTEMS

### P284 A literature review for developing the clinical phenotype evaluation system of Atopic Dermatitis

#### Jinhyang Ahn, Younghee Yun

##### Kyung Hee University Hospital at Gangdong, Seoul, 05278, South Korea

###### **Correspondence:** Jinhyang Ahn (hyaji87@naver.com)

**Purpose**

It is important to determine and analysis on the clinical symptoms and physical symptoms in Korean oriental medicine practice of atopic dermatitis.There is a surface on which the existing evaluation items and methods in atopic dermatitis have been somewhat simplified to apply to actual clinical practice.Thus it is necessary to evaluate whether the development of standardized symptoms and diagnostic tool to evaluate atopic dermatitis objectively.

**Methods**

We performed a literature review for developing the clinical phenotype evaluation system of atopic dermatitis. First step, two independent searcher searches for paper by entering the search term through the search engines : Oriental Medicine Advanced Searching Integrated System(OASIS) and Korean Studies Incategoryation Service System(KISS). We looked through all the papers and finally chose 47 papers that describe symptoms for atopic dermatitis and suitable for inclusion. Then, we extracted symptoms from these papers and arranged them in order of frequency and validity through experts' conference.

**Results**

We found 360 papers and chose 47 papers. After we reviewed the final papers, we decided to include general information of patients, systemic and dermatologic symptoms in evaluation category of atopic dermatitis. Through experts' conference, it was decided that general information has age, sex and body type; Systemic symptoms have 9 items; Dermatologic symptoms have 15 items.

**Conclusions**

Through a literature review and experts' conference, we developed a clinical phenotype evaluation system of atopic dermatitis complementing existing assessment. Therefore it was prepared the grounds for evaluation of atopic dermatitis.

### P285 Double cupping versus single cupping in chronic low back pain (CLBP): Randomized controlled trial

#### Sulaiman AlEidi^1^, Ashry Gad Mohamed^2^, Abdullah M Al-Beda^1^, Raid A Abutalib^3^, Mohemmed KM Khalil^1^

##### ^1^ National Center for Complementary and Alternative Medicine, Riyadh, Saudi Arabia ^2^ Family and Community Medicine Department, King Saud University, Riyadh, Saudi Arabia ^3^ Orthopedic Surgery, King Fahad Hospital, Madinah, Saudi Arabia

###### **Correspondence:** Sulaiman AlEidi (smaleidi@gmail.com)

**Background and objective**

Local traditional healers in Saudi Arabia claims that the wet cupping technique used in the Middles East is more effective than the Asian technique. To investigate whether there is a difference in the effectiveness between the traditional (cupping – scarification with surgical blade – cupping) commonly used in Saudi Arabia and the Asian (puncture with auto-lancet – cupping) wet cupping (AlHijamah) techniques in the management of CLBP.

**Methods**

A multicenter randomized comparative clinical trial. Seventy patients with chronic low back pain were randomized to receive one session of wet cupping (AlHijamah), whether with traditional local technique or with the Asian technique. Numeric Rating Scale (NRS), Persistent Pain Intensity (PPI), and Oswestry Disability Questionnaire (ODQ) scores on Day 7 and Day 14 after the intervention were used as outcome measures.

**Results**

The two wet cupping therapy techniques groups have shown a significantly decreased in NRS, PPI, and ODQ scores at Day 7 and Day 14 after the intervention within each group (*P* = <0.001) across all the outcomes measures within each group, but there were no significant differences across all the outcome measures between the two groups up to 14 days after the intervention across all the outcomes measures between the two groups. The study did not document any adverse events or serious adverse events from the interventions.

**Conclusion**

Wet cupping is effective in reducing pain and improving disability in patients with chronic low back pain. Both single (Asian) and double (local traditional) wet cupping techniques are equally effective.

### P286 Unani medicine and its emphasis on health preservation and prevention

#### Hakima Amri (amrih@georgetown.edu)

##### Biochemistry and Cellular and Molecular Biology, Georgetown University Medical Center, Washington, D.C., United States

Unani Medicine or *Unani Tibb* also called Greco-Arabic or Perso-Arabic Medicine is a complete medical system based on the teachings of Hippocrates (460-370 BC). The principles and concepts were modernized and compiled by Avicenna (980-1037 CE) in his five volumes named *The Canon of Medicine*. While he systematically recorded disease etiology, diagnosis and treatment, he also strongly emphasized the importance of healthy life style and preventive medicine that Western countries are stressing today, in the twenty first century.

In this work, we focus on one angle of Unani Medicine summarized in the six steps toward achieving healthy living, and their impact on the modern explanatory model of health preservation and disease prevention. Furthermore, modern scientific discoveries supporting Avicennas observations will be discussed. The six steps are presented as follows: (1) breathe fresh clean air, (2) eat nutritious food and drink, (3) alternate between movement and rest, (4) respect sleep and wakefulness cycles, (5) ensure regular eating and bowel movement, and (6) maintain healthy mental state. This stems from Unani Medicines practice of healthy bodies management or *Science of Health Preservation* that is called today Preventive Medicine and Western physicians are striving to implement, in order to face the growing metabolic epidemics.

Although Avicennas explanation of each step is based on the theories and principles of his time (humors, elements, and temperaments), but his logic holds still true today in light of our modern explanatory models of health and disease and scientific discoveries.

### P287 A scientific evaluation of Hiranyapraash TM an ayurvedic nanomedicine as a natural immune booster in children

#### Sathyanarayana Badekila (bhaishajya@yahoo.com)

##### Ayurveda, Muniyal Institute of Ayurveda Medical Sciences, Manipal, 576104, India

In the present day protecting child from endless germs and viruses is the need of hour. Some pediatricians consider in a year six to seven bouts of flu or ear infections in children are normal. But there are healthy habits by which the childs immunity can be boosted and reduction in episodes of illness can be achieved. Adequate sleep, nutrition, regular exercise, hygiene maintenance, being away from allergens and germs are the good practices which can reduce the morbidity and boost the immune system. One such practice is *Swarna Prashana* explained in ancient literatures of *Ayurveda* which enhances the healthy status of child. Hiranya PraashTM is a patented, research product designed and developed by Dr. Krishna Life Science Ltd., Manipal. It is safe, natural rejuvenative with the power of gold. Pure gold is processed with selected organic, bio-active herbs by using patented techniques.

**Material and Methods:**

1. An assessment of Cell mediated immune function by delayed type hypersensitivity (DTH) test.
2. Survey was carried out in various centres related the institution and hospital. 104 children randomly assessed for clinical study. 2 drops of Hiranyaprash™ administered to the children below the age group of 5 years and 4 drops administered above the age group of 5 years. The survey and clinical assessment was done and data obtained was analyzed statistically.

**Conclusion:**

1. Study has proved immune stimulant activity of Hiranyapraash.
2. It is found to be Hiranyaprash™is beneficial in preventing the respiratory manifestations and beneficial in increasing the appetite of children. Analysis of data on behavioural assessments shows high statistical significance in parameter school work performance and subject understanding ability.

### P288 Al-Zahrawi, the first physician who described dysmenorrhea

#### Elham Behmanesh, Seyyedali Mozaffarpour

##### School of Traditional Medicine and History of Medical Science Research Center, University of Medical Sciences, Babol, 47176477, Iran, Islamic Republic of

###### **Correspondence:** Elham Behmanesh (bahmanesh_e@yahoo.com)

Menstrual period is one of the girls puberty stages, that any change can spite a woman's sex life to natural fertility or infertility. Dysmenorrhea is a kind of pelvic pain that 50-90% of reproductive women experience during their life. It is the greatest cause of lost working and school days among young women and decrease quality of life. There is a great trend to use Traditional Medicine recommendations and prescribes in the world. Therefore searching the literatures and finding common points in Traditional Medicine and Gynecology is necessary. In this way, opening up new avenues in the treatment and control of dysmenorrhea, possibly avoid wasting time and communitys investment and enhance the quality of life of women. There are many management line for women in the TPM literatures and has been interest for physicians throughout the history, but among menstrual changes, dysmenorrhea is not the earliest concern. While searching the ancient PM texts during 9th -19th AD, there are many overlapping conditions, which may mimic to this disorder, there is not any particular terms and definitions for dysmenorrhea and it is mentioned in Persian Medicine literature under different names such as Oja-e Rahem (Uterus pain), Osr-o Tams (dysmenorrhea) and Oja-e Zahr (back pain). First explanation of dysmenorrhea was found in the Al-Zahrawis masterpieces Al-tasrif in detail. It can be helpful to test this suggestions as ideas for clinical researches. Key words: Dysmenorrhea- Al-Zahrawi- Menstruation- Traditional Persian Medicine.

### P289 Comparison between the theory of Arkan (four elements) in Traditional Persian Medicine and the theory of five elements in Traditional Chinese Medicine

#### Elham Behmanesh, Seyyedali Mozaffarpour

##### Traditional Medicine, University of Medical Science, Babol, 4717647745, Iran, Islamic Republic of

###### **Correspondence:** Elham Behmanesh (bahmanesh_e@yahoo.com)

Traditional medicines, according to the World Health Organization, are defined as the sum total of the knowledges, skills, and practices based on the believs, theories, and experiences indigenous to different cultures that are based and developed historically. Due to the increasing the application of traditional medicines worldwide, it seems that cognition of their principles and foundations are necessary. Traditional Persian Medicine (TPM) and Traditional Chinese Medicine (TCM) have many similar concepts. Theory of Arkan in TPM and five Elements in TCM are one of the most fundamental similarity. This is a review study, which extracted, classified and compared related concepts in TPM and TCM, conducted on original sources. The Web databases SID, Magiran, Iranmedex, Google scholar, Pubmed and Embase were searched to use the experiences of other researchers and find published articles in this regard. The results were categorized under 12 categories. Generally, although there are considerable similarities between theory and functions of Arkan in TPM and theory of five elements in TCM. There are differences between the basic principles and the outcomes of both theories. Comparison between these theories shows that the source of both in philosophy are the same. TPM arises from a monotheistic (not necessarily Islamic) viewpoint while TCM comes mostly from a metaphysical point of view. Furthermore, there are four principles in TPM but five in TCM. In addition, principles in TCM and TPM differ as follows: while in the former, the principles (having interactions in a dynamic process) are assumed to be directly related to phenomena, in the latter, they are explained with the mediated concept of Mizaj.

### P290 The study of menstrual pain etiologies in Traditional Persian Medicine and comparison with current medicine

#### Elham Behmanesh^1^, Pantea Shirooye^2^, Razie N Meybodi^2^, Roshanak Mokaberinejad^2^, Mojgan Tansaz^2^, Seyyedali Mozaffarpour^1^

##### ^1^ Traditional Medicine, University of Medical Science, Babol, 4717647745, Iran, Islamic Republic of; ^*2*^*Traditional Medicine, Shahid eheshti University of Medical Science, Tehran, 1516745811, Iran, Islamic Republic of*

###### **Correspondence:** Elham Behmanesh (bahmanesh_e@yahoo.com)

**Introduction**

Menstrual pain or dysmenorrhea is the most common cause of pelvic pain that followed by many consequences and it has remained as a health problem. This study compared menstrual pain in Traditional Persian Medicine (TPM) vs currentmedicine.

**Methods**

This study investigate definitions, etiologies, manifestations and prognosis of menstrual pain in TPM references, Gynecological textbooks and databases through multiple and associated keywords. Then the findings were compared.

**Result**

Menstrual pain is called "usr o tams" in TPM. Usr o tams is divided into primary and dependent based on the delay or no delay in puberty. On the other hand, Dysmenorrhea is divided into primary and secondary based on the presence or absence of pelvic pathology. Risk factors of both points are similar, but "mizaj" in TPM view of point is important, too. Etiology in both views is reduction of uterine blood flow due to uterine vessel stenosis, while TPM's references has explained blood viscosity due to phlegm ("Balgham") and black bile ("Sauda") as another cause of reduction in uterine blood flow. In TPM, manifestation is divided into first and second degree that they are in accordance with the main symptoms of dysmenorrhea and premenstrual syndrome, respectively.

**Conclusion**

Separation of primary and dependent usr o tams with history is the first step of managing the menstrual pain. The second step is separation of primary and secondary dysmenorrhea by history and ultrasonography. The cause of primary dysmenorrhea should be investigated that uterine vessel stenosis or blood viscosity. Blood viscosity explains unknown reasons and lack response to treatment of dysmenorrhea.

### P291 Is individualized Chinese herbal medicine treatment more effective than standardized treatment? A meta-epidemiological study

#### Vincent CH Chung, Xin Y Wu, Justin CY Wu

##### Jockey Club School of Public Health and Primary Care, The Chinese University of Hong Kong, Shatin, Hong Kong, Hong Kong

**Purpose**

Classically, Chinese herbal medicine (CHM) is prescribed in an individualized fashion according to syndrome differentiation results grounded in Chinese medicine diagnostic theory. But in modern practice CHM is also used in a standardized manner for a specific biomedical diagnosis. It is argued that the individualized approach would yield better outcome but this is yet to be evaluated. This meta-epidemiological study aims to investigate difference in effect estimation between randomized controlled trials (RCT) of individualized and standardized CHM treatment.

**Methods**

We searched Cochrane Database of Systematic Reviews from its inception till March 2016. Meta-analysis which contained RCTs evaluating both standardized and individualized CHM treatment were included. Ratios of odds ratios (RORs) and difference in standard mean difference (SMD) were used to measure the difference of effect estimation between individualized and standardized treatment for dichotomous and continuous outcomes, respectively.

**Results**

Twelve meta-analyses (119 RCTs) were included. No significant difference in treatment effects was found between individualized and standardized treatment for dichotomous outcomes (pooled ROR = 0.85, 95% confidence interval [CI]: 0.28 to 1.41, 9 meta-analyses, I2 = 70.7%). Pooled ROR were 0.79 (95% CI: 0.32 to 1.26, 5 meta-analyses, I2 = 0.0%) and 1.26 (95% CI: 0.64 to 1.89, 3 meta-analyses, I2 = 0.0%) for subjective and objective dichotomous outcome, respectively. For continuous outcomes, pooled difference in SMD is 0.11 (95% CI: -0.12 to 0.34, 3 meta-analyses, I2 = 70.1%), without statistical significance. Sensitivity analysis was conducted by excluding the most heterogeneous meta-analysis, which allowed us to focus only on objective continuous outcomes. This generated a small pooled difference in SMD of 0.24 (95% CI: 0.06 to 0.41, 2 meta-analyses, I2 = 0.0%).

**Conclusions**

From existing data, we found no significant difference between individualized and standardized CHM treatment on both dichotomous and continuous clinical outcomes. Further research with a larger sample of meta-analyses is needed to confirm these findings.

### P292 Introducing the concept of *Musleh* (ameliorating agent) as a key point for drug discovery in traditional medicines

#### Babak Daneshfard^1^, Ayda Hosseinkhani^2^, Vahid Tafazoli^2^, Amir M Jaladat^2^

##### ^1^ Student Research Committee, Shiraz University of Medical Sciences, Shiraz, Iran, Islamic Republic of; ^*2*^*Research Center for Traditional Medicine and History of Medicine, Shiraz University of Medical Sciences, Shiraz, Iran, Islamic Republic of*

###### **Correspondence:** Babak Daneshfard (babakdaneshfard@gmail.com)

Human body is an extremely complex system and it is so simplistic to think that an active agent working on a single receptor is enough for an optimal clinical response. There is a growing body of evidence shows the necessity for shifting to a multitarget approach in the field of drug discovery. Traditional medicines which have holistic viewpoints in maintaining the overall balance of the body usually put forward such approach in their drug formulations as suggested in systems biology. It may be the reason of recent more interest of pharmaceutical companies for research in traditional medicines. Nonetheless, it has its own troubles; multicomponent formulations, possible toxicities, and lack of our knowledge regarding their mechanism of action are some of the obstacles. Understanding the concept of ameliorations –which means modulation of the effects and/or counteracting the side effects of ingredients of a compound formulation by one of its constituents– mentioned as *Musleh* in Traditional Persian Medicine and *jun-chen-zuo-shi* in Traditional Chinese Medicine, could pave the way for further researches in this field.

### P293 Dietary recommendations in fracture healing in Traditional Persian Medicine: A historical review of literature

#### Amir M Jaladat, Hasan Sadeghi

##### Shiraz University of Medical Sciences, Shiraz, Iran, Islamic Republic of

###### **Correspondence:** Amir M Jaladat (drjaladat@gmail.com)

**Question**

Fracture repair is a complex process. An inappropriate diet is a contributing risk factor in the non-union of a fracture. The aim of this study was to extract dietary recommendations for fracture healing from literature on traditional Persian medicine (TPM).

**Methods**

The content relevant to diet in fracture healing was selected from the main textbooks in TPM, like *Al Qanon fi Al-teb* (The Canon). Other reference textbooks in traditional medicine were used to achieve a comprehensive study in this respect. Finally, content analysis was used to summarize and describe the results.

**Results**

Foodstuffs are classified in TPM according to their nutritive value, their assimilability, and the quality of achieved chyme. Some light meals like chicken soup are recommended for the early days of fracture, while high-nutrient and dense foods such as goats or sheeps head and nuts are advised on the following days for acceleration of fracture healing and callus formation. Several recommendations are also provided for pacing the healing process.

**Conclusions**

A comparison of the regimens recommended by Avicenna and other Persian sages with recent evidence revealed the potential positive effects of their regimen for acceleration of bone healing. This study can shed light on a part of the history of orthopaedics, and add to current knowledge about bone fracture and its management.

### P294 Shuxuetong injection for ischemic stroke: An overview of systematic reviews

#### Liyan Jia^1^, Nanqi Zhao^1^, Xiaoyi Yan^1^, Li Zhou^2^, Meng Zhao^2^, Weiwei Li^3^, Jianping Liu^1^, Zhaolan Liu^1^

##### ^1^ Center for Evidence-based Chinese Medicine, Beijing University of Chinese Medicine, Beijing, China; ^*2*^*Dongzhimen hospital, Beijing University of Chinese Medicine, Beijing, China;*^*3*^*Xiyuan hospital, Beijing University of Chinese Medicine, Beijing, China*

###### **Correspondence:** Zhaolan Liu (lzl1019@163.com)

**Objective**

This overview is to summarize the systematic reviews (SRs) of Shuxuetong injection for ischemic stroke (IS), and to evaluate the methodological quality and the current evidence of these SRs.

**Methods**

We searched six databases (including CNKI, Wan Fang, VIP, SinoMed, Cochrane Library and PubMed) until October 2016, and included the SRs of RCTs concerning Shuxuetong injection for IS. AMSTAR scale was used to evaluate the methodological quality.

**Results**

Ten reviews (involve 136 RCTs with more than 11508 participants) were included in this overview, the AMSTAR scales of these reviews were among 3 to 7 with an average of 5.6. Most of reviews assessed clinical efficiency (9/10, 90.0%), neurological deficits score (8/10, 80.0%) and adverse events (7/10, 70.0%). Some reviews assessed mortality (1/10, 10.0%), cure rate (1/10, 10.0%), efficiency (1/10, 10.0%) and activities of daily living (1/10, 10.0%). We find the overall effect of Shuxuetong injection was better than the control group. Seven reviews (involve 54 RCTs)reported the outcome of adverse events, 43 RCTs showed there were no adverse reaction and 11 RCTs showed there have a slight adverse reaction (including poor appetite, nausea, vomiting, subcutaneous ecchymosis, rash, low thermal or facial redness head bilges).

**Conclusion**

Maybe there have marked advantages about Shuxuetong injection for IS, but we should more high quality studies to make a judge. We can report reviews according to AMSTAR scale for a standardization report. And the experts of evidence-based medicine should carry out more training courses about the methods of clinical research and SRs.

### P295 Xingnaojing injection for ischemic stroke: An overview of systematic reviews

#### Liyan Jia^1^, Nanqi Zhao^1^, Xiaoyi Yan^1^, Li Zhou^2^, Meng Zhao^2^, Weiwei Li^3^, Jianping Liu^1^, Zhaolan Liu^1^

##### ^1^ Center for Evidence-based Chinese Medicine, Beijing University of Chinese Medicine, Beijing, China; ^*2*^*Dongzhimen hospital, Beijing University of Chinese Medicine, Beijing, China;*^*3*^*Xiyuan hospital, Beijing University of Chinese Medicine, Beijing, China*

###### **Correspondence:** Zhaolan Liu (lzl1019@163.com)

**Objective**

This overview is to summarize the current evidence from systematic reviews (SRs) of Xingnaojing Injection for ischemic stroke (IS)and evaluate their methodological quality. **Methods**

We included SRs of Xingnaojing Injection for IS until October 2016 by searching CNKI, Wan Fang, VIP, SinoMed, Cochrane Library and PubMed. AMSTAR scale was used to evaluate the methodological quality and we summarize the evidence by classify the outcomes.

**Result**

Nine SRs involved 145 RCTs and more than 12594 participants were included, their AMSTAR scales were among 2 to 7 with an average of 4.7.For outcomes, most of the reviews assessed clinical efficiency (9/9,100%), neurological deficits score (7/9,77.8%) and adverse events (6/9,66.7%). Some reviews assessed mortality (3/9,33.3%), cure rate (2/9,22.2%), efficiency (1/9,11.1%) and Glasgow coma scale (2/9,22.2%).Most reviews showed the Xingnaojing group have marked advantages over the control groups in the outcomes of clinical efficiency and neurological deficits score. One review (involved 5 RCTs) reported there was no adverse reaction and five reviews showed there only have a slight adverse reaction (e.g. skin rash, nausea, dizziness, flustered and mild liver and kidney damage).

**Conclusion**

Xingnaojing Injections for IS may have marked advantages; however the methodological quality of the reviews was awful and there need more high quality studies to make a judge. Whats more, researchers should report more outcomes about the endpoint indicators (e.g. the cure rate, response rate and disabled rate, recurrence rate) and the outcomes closed to the patients (e.g. the quality of life scores and the economic burden).

### P296 "There are more things in heaven and earth." How knowledge about traditional healing affects clinical practice: A qualitative study

#### Anette L Larsen^1^, Anita Salamonsen^1^, Agnete E Kristoffersen^1^, Torunn Hamran^2^, Bjørg Evjen^3^, Trine Stub^1^

##### ^1^ Department of Community Medicine, NAFKAM, The Arctic University of Norway UiT, Tromsø, 9037, Norway; ^*2*^*Department of Health and Care Sciences, The Artic University of Norway, Tromsø, 9037, Norway;*^*3*^*Centre for Sami studies, The Actic University of Norway, Tromsø, 9037, Norway*

###### **Correspondence:** Anette L Larsen (anette.l.larsen@uit.no)

**Background**

The Sami today are closely connected to the traditional folk medicine. Religious prayers of healing (*reading*) and the laying on of hands are examples of the methods that they employ. In this study we will examine the knowledge and attitudes among health personnel regarding traditional healing in areas where this is used, and how this knowledge affects their clinical practice.

**Method**

Semi-structured individual interviews (*n* = 32) and focus group interviews (*n* = 2) were conducted among health personnel in two Sami communities in Norway. The text data was transcribed verbatim and analyzed based on the criteria for content analysis. The codes were defined prior to and during the data analysis (mixed type). Six themes were identified.

**Result**

The participants had acquired their knowledge of traditional healing through their childhood, adolescence and experience as health personnel in the communities. They were all positive to the patients’ use of traditional healing. They justified their attitudes stating there are more things in heaven and earth, and they had faith in placebo effects of the treatment. The health personnel respected their patients’ faith and facilitated the use of traditional healing. In some cases they also conducted rituals on patients who wanted this. In this way they changed their clinical practice.

**Conclusion**

The health personnel were positive and open-minded towards traditional healing. According to the informants *reading* was a tool that helped patients to handle illness in a good way. The health personnel changed their clinical practice to meet the needs of their patients. In this manner they could offer their patients integrated health services which were tailored to the patients’ culture.

### P297 Pedietric massage for childhood diarrhea: A Chinese ancient books' review

#### Meiling Li^1^, Jianxiong Cai^2^, Taoying Lu^2^, Lingjia Yin^2^, Darong Wu^2^, Lixin Wang^3^

##### ^1^ Guangzhou University of Chinese Medicine, Guangzhou, 510000, China; ^*2*^*The Second Affiliated Hospital of Guangzhou University of Chinese Medicine, Guangzhou, 510120, China;*^*3*^*The Affiliated Hospital of Changchun University of Chinese Medicine, Changchun, China*

###### **Correspondence:** Meiling Li (865955758@qq.com)

**Objective**

It has been a long history in the Chinese Medicine of applying pediatric massage therapy in the treatment of children with diarrhea. In order to provide reference in choosing massage acupoints in the treatment of childhood diarrhea, we reviewed relevant ancient literature.

**Method**

Based on a national questionnaire survey through a group of professional pediatric massage practitioners via Wechat, we finalized the ancient books that would be included. We applied the following keywords (in Chinese language,which all mean diarrhea), for searching,i.e. Xie (泻), Xie (泄), Xiexie (泄泻), Li(痢), Tang(溏), Li(利), Zhuxia(注下), in the fifth electronic version of Chinese Medical Classics (v2.0-2006). We manually searched the books that could not be found in the electronic system. All the items about childhood diarrhea were put into an excel for data extraction. We included the items that were associated with pediatric massage acupoints, manipulation and treatment prescription, and excluded those belonged to the etiology and pathogenesis, syndromedifferentiation or other treatments of pediatric diarrhea.

**Result**

Among all the 150 professionals who received the questionnaires, forty eight (32%) of them responded. Twelve ancient books were recommended by more than half of the respondents. With the electronic and manual search of theses twelve books, we detected 3,001 items, among which only 30 items were eligible for inclusion. All together, 29 acupoints or manipulations were mentioned, among which Dachang and Sanguan were addressed for ten times respectively. Several other acupoints, e.g. Guiwei (DU1), Qi (RN8), Shouyinyang, Pi, were mentioned from five to nine times.

**Conclusion**

The information we found from ancient Chinese books in this study may provide valuable references in helping the researchers or practitioners to decide which acupoints they shall put into consideration in regards of the pediatric massage therapy for children with diarrhea.

Keywords: pediatric massage, diarrhea, children, Chinese ancient book, review

### P298 Comparison of anxiolytic effects of the homeopathic complex Vita-C 15 in compared with Aconitum napellus in the acutely stressed C57BL6 mice

#### Siaw M Liew (charisliew88@gmail.com)

##### Cyberjaya University College of Medical Sciences, Cyberjaya, Malaysia

Anxiety, phobias and stress are the main mental health problems among the Malaysian population, with global prevalence varying from 8% to 18%. Even so, less than 30% who suffer these disturbances seek treatment (Harvey and Champe, 1998). The objective of this study is to evaluate and compare the anxiolytic effects of *Aconitum napellus* and Homeopathic complex Vita-C 15 in the acutely stressed C57BL6 mice by using the corticoid test, open field test (OFT) and c-fos, NMDAR 2B, NPY 1R and NPY 2R activity through the hippocampus. Methods: A double blinded randomized controlled study is conducted. All the animals are acclimatized to constant laboratory conditions for 14 days before starting the experiments. Prior to the experiment, a pilot study is performed to identify the most ideal potency for the homeopathic remedy of *Aconitum napellus*. The animals are tested (*n* = 3) per group on the potency of 6 C, 30 C and 200 C. The treatments are carried out over 9 days. 48 male C57BL6 mice (*n* = 6), 4-5 weeks of age are used. They are randomly selected and divided into two groups. Group I is the healthy control group of mice which are not exposed to acute stress. Group II (stress group); comprise of mice expose to acute restraint stress. Prior to restraint stress, the treatments given are *Aconitum napellus* 30 cH, Homeopathic complex Vita-C 15, Diazepam and placebo. The results are evaluated and compared by CORT test, open field test and immunochemistry test. *Aconitum napellus* and Homeopathic complex Vita-C 15 are expected to have anxiolytic effects in the acutely stressed C57BL6 mice. Together, these findings suggest a potential role of stress hormones, such as corticosterone (CORT) in mice, in the pathology and treatment of anxiety and stress. Thus research into prevention and supportive therapies is necessary and beneficial for this disorder.

### P299 Research on anti-inflammatory mechanisms of Chinese medicine treating preschool children with pneumonia based on methods of data mining and network pharmacology

#### Tiegang Liu, Chen Bai, Zian Zheng, Yuxiang Wan, Jingnan Xu, Xuan Wang, He Yu, Xiaohong Gu

##### Beijing University of Chinese Medicine, Beijing, 100029, China

###### **Correspondence:** Xiaohong Gu (GUXH1003@126.com)

**Purpose**

Based on the methods of data mining and network pharmacology, it is to explore the targets and mechanisms of anti-inflammatory actions with traditional Chinese medicine (TCM) treating the preschool children with pneumonia and provide the targeted guidance for subsequent experimental study.

**Methods**

Entropy clustering analyzing the literatures of case reports about the preschool children with pneumonia treated by TCM, it was to extract the new prescriptions. A database of inflammation-related action targets for new core prescriptions was established by the Traditional Chinese Medicine Systems Pharmacology Database (TCMSP). And the interactions between targets were analyzed by Search Tool for the Retrieval of Interacting Genes/Proteins (STRING). Moreover, the inflammation-related signaling pathways of new prescriptions were analyzed by Kyoto Encyclopedia of Genes and Genomes (KEGG).

**Results**

We obtained 6 new prescriptions for the treatment of pneumonia in preschool children and 14 inflammation-related action targets. In the network comprised with those action targets, there were 14 key actions and 22 signaling pathways (FDR < 0.01). Among those signaling pathways, MAPK signaling pathway, Fc epsilon RI signaling pathway and Inflammatory mediator regulation of TRP channels had the most closely relationship with inflammation.

**Conclusion**

The anti-inflammatory action of TCM treating the preschool children with pneumonia was achieved by the intervention of multiple targets with complex signaling pathways together. Yet the specific regulatory mechanism is to be further experimental studies to explore and verify.

### P300 Chinese patent medicine for diabetic retinopathy: A systematic review of randomized controlled trial

#### Zhaolan Liu, Xiaoyi Yan, Liyan Jia, Nanqi Zhao, Guoyan Yang, Jianping Liu

##### Beijing University of Chinese Medicine, Center for Evidence-based Chinese Medicine, Beijing, China

###### **Correspondence:** Jianping Liu (jianping_l@hotmail.com)

**Objective**

To evaluate the effectiveness and safety of Chinese patent medicine for treatment of diabetic retinopathy (DR).

**Method**

A comprehensive searching has been conducted in six databases from their inception until December 2015. We included randomized controlled trials (RCTs) that tested Chinese patent medicine for DR. Two authors independently screened for inclusion, extracted data and assessed the risk of bias (Cochrane risk of bias tool). We applied RevMan 5.3 to analyze data.

**Result**

A total of 20 articles and 1941 participants were included. One trial was assessed as low risk of bias but the other trials were high.16 kinds of Chinese patent medicine were identified which contained 7 kinds of formulation and 6 kinds of comparison types. Due to the substantial heterogeneity among studies, only 9 studies were pooled in nine Meta-analyses. Two meta-analyses showed that Chinese patent medicine plus conventional therapy was superior to the placebo plus conventional therapy in decreasing the regions of retinal capillary non-perfusion and capillary leakage (3 trials, *n* = 159, MD:-0.08PD,95% CI: [-0.14,-0.02]; 3 trials, *n* = 159, MD:-0.11PD,95% CI: [-0.18,-0.03], respectively). One trial reported that one participant had a stroke in the study due to other reasons. No severe adverse events reported.

**Conclusion**

Chinese patent medicine is potentially effective and well tolerated in patients with DR in reducing the regions of retinal non-perfusion and capillary leakage. Considering the small sample size and substantial heterogeneity among studies, the findings should be interpreted with caution and verified in future research.

### P301 Contribution of Al-Zahrawi (Abulcasis) to dysmenorrhea

#### Seyyedali Mozaffarpour, Elham Behmanesh

##### Traditional Medicine and Medical history Research Centre, University of Medical Science, Babol, Iran, Islamic Republic of

###### **Correspondence:** Seyyedali Mozaffarpour (Seyyedali1357@gmail.com)

Dysmenorrhea is a frequent medical condition with painful menstrual cramps that interferes with daily activities. The menstrual pain begins a few hours before the onset of menstrual flow and may be lasted for 2 to 3 days. Frequently associated symptoms may be present and usually subsides as menstruation tapers off.One of the best recorded observations for dysmenorrhea was in Al-Tasrif of Al-Zahrawi (936-1013). He made a great contribution to gynecology including dysmenorrhea and ectopic pregnancy for the first time in the history of medicine. The aim of this study is to consider Al-Zahrawis description about dysmenorrhea including definition, etiology and intervention. Al-Zahrawi believed in humoral theory and he categorized the mechanisms of dysmenorrhea into three types. Most of his opinions can be compared with current medical concepts. He suggested therapeutic plans including life style, oral and topical drugs. Current findings show most of medicinal plants mentioned by Al-Zahrawi can reduce pain in women with dysmenorrhea.

### P302 Body temperature and health in Traditional Persian Medicine

#### Majid Nimrouzi, Vahid Tafazoli, Babak Daneshfard

##### *Shiraz University of Medical Sciences, Shiraz, Iran, Islamic Republic of*

###### **Correspondence:** Majid Nimrouzi (meshkat_114@yahoo.com)

**Background**

The thermoregulatory control of skin blood flow is vital for maintenance of core body temperatures. Complete and proper functioning of the body is dependent on maintaining a body core temperature between 36.5 to 38.5 °C. Thyroid hormones contribute an important role in control of the body temperature. The action of thyroid hormones, secretion of norepinephrine and the presence of free fatty acids activate uncoupling proteins in the skeletal muscles, that play role in the skeletal muscle heat production. The diagnostic approach and medical interventions in Traditional Persian medicine is very different with conventional medicine because of different basics.

**Objective**

We aimed to compare the concept of heat and its effects on health between conventional and traditional Persian medicine.

**Results**

According to literature, maintenance of the internal heat increases longevity in animals but human studies are scarce in this issue. Traditional Persian medicine sources, however, confirms the role of innate heat preservation on longevity and health. Despite the differences between traditional and conventional approach to the subject of heat and health, there are common views about the importance of maintaining the internal body temperature on homeostasis and health in human being.

**Conclusion**

We are about to open a new window to issue of body homeostasis according to traditional medicine resources hoping to provide a context for interested researchers in this field to put enormous potential of traditional medicine into practice by well-designed clinical trials.

### P303 *'Āina Hoʻoulu Lāhui*: Traditional Hawaiian agricultural technology as integral to health and Mauli Ola

#### Deja Ostrowski, Kealoha Fox

##### Office of Hawaiian Affairs, Honolulu, 96817, HI, United States

###### **Correspondence:** Kealoha Fox (kealohaf@oha.org)

**Purpose**

Traditional Hawaiian agricultural technologies are integrated approaches such as loko I’a (fishponds), māla (cultivated gardens), and lo’i (irrigated patches) that historically sustained significant populations with complete self-sufficiency within the Hawaiian archipelago. These form the basis for population health strategies which rely on the abundance of traditional Hawaiian foods that balanced the Hawaiian mind, body, and spirit. Expanding ancient agroecology associated with indigenous farming today may help meet Hawaiʻi’s growing demand for food in a manner that is socially equitable, economically stable, nutritious, and ecologically sustainable over the long term. Hawaiian crops act as critically important resources for traditional knowledge and help to perpetuate Native Hawaiian culture as an essential component of the state of Hawaiʻi’s strategic, economic and social wellbeing.

**Methods**

Utilizing social determinates of health modeling; advocacy was focused to bring research theory to policy implementation as a long-term prevention strategy. Using evidence-informed policy methods, we conducted interviews with community stakeholders (*N* = 14) and Hawaiian practitioners (*N* = 18) to eliminate barriers to their traditional practices and customary rights. Cross referencing state objectives that prioritized out-dated plantation based agricultural methods; this legislative update reflects community-driven efforts to holistically feed Hawai'i's communities through traditional Hawaiian farming practices and small farms.

**Results**

The Hawai'i Governor signed the bill SB434 SD2 HD1 into law as Act 031 on May 5, 2015 to include "Traditional Hawaiian Farming Systems and Small Scale Farming" to Hawaiʻi State Planning Objectives. This bill updates the Hawai’i State Planning Act’s (HSPA) agricultural objectives to support traditional Hawaiian farming techniques and crops, and small farms within an integrated values-based health ecosystem. This bill creates a statutory priority to diversify an improved agricultural portfolio where both small and large farming operations provide Hawai'i's local food production with the greatest protection against pest introductions, disease or climate-change related impacts.

**Conclusion**

During the 27th Legislature we authored, introduced and passed legislation to amend Hawaiʻi Revised Statutes 226-7 to include traditional Hawaiian farming systems, traditional Hawaiian crops, and small-scale farming to the HSPA. This is the first state bill of its kind to expand the State's efforts to further food security and self-sufficiency within the traditional Hawaiian model of Mauli Ola. Whereas, *"*traditional Hawaiian crops…that were cultivated using these traditional Hawaiian farming techniques continue to be important agricultural products for food, medicine, and cultural practices today."

(University of Hawaii Human Subjects Protection review approved CHS#23530).

### P304 The therapeutic effects of fennel in Traditional Persian Medicine: A review

#### Mehdi Pasalar, Fatemeh Tabatabei, Fatemeh Amini

##### Research Centre for Traditional Medicine and History of Medicine, Shiraz University of Medical Sciences, Shiraz, Iran, Islamic Republic of

###### **Correspondence:** Mehdi Pasalar (pasalar@sums.ac.ir)

**Purpose**

Fennel (*Foeniculum volgarea)* is a popular plant in Traditional Persian Medicine (TPM) as well as a customary spice in Iranian foods. The aim of current study is to compare the traditional uses of fennel, based on TPM resources, with the recent evidence based findings for treatment of various diseases.

**Methods**

We considered TPM texts like "Makhzan-al-Advyeh", "Tohfeh", "Exir-e-aazam" and, "teb-e-Akbari" searching for therapeutic effects of fennel and its derivative formulas. Then, we look for English and Persian databases including pubmed, web of science, scopous, google scholar, chocrain library, SID, Iran medex and, Magiran in order to find new published surveys in this field.

**Results**

The use of medicinal plants in the treatment of many diseases is in rise nowadays. Although therapeutic effects of plants have not been proven completely, patients’ tendency toward the use of medicinal plants is remarkable. There is a long list of therapeutic actions in TPM books including pain killer, dieresis, anti-diarrhea, menstruation initiator and mothers’ milk stimulating agent (Table 2). Some of aforementioned activities have been certified in up-to-date literature based on clear-cut sophisticated research.

**Table 2 (Abstract P303).** Some recognized therapeutic effects of fennel in TPM and modern medicine

Fennel therapeutic effects	TPM	Modern medicine
Respiratory diseases	+	+/-
Stomach pain	+	+
Anti flatulence	+	+
Constipation	+	+
Menstruation initiator	+	+
Pain killer	+	+
Mothers’ milk secretion	+	+

**Conclusions**

TPM references introduce several health benefits for fennel as a healing agent which have been ascertained lately. On the other hand, there are some unproved claims in TPM which needs rigorous high-quality investigations to be ruled in or not. This may help for introducing more effective therapeutic medicaments for recent health dilemmas.

Keywords: Foeniculum volgarea, Therapeutic, Traditional Persian Medicine

### P305 Plants currently used to treat diabetes in Sri Lankan Siddha Medicine – an ethnobotanical survey in the Eastern Province

#### Saravanan Sathasivampillai^1^, Pholtan Rajamanoharan^2^, Michael Munday^1^, Michael Heinrich^1^

##### ^1^ School of Phramacy, University College London, London, WC1N 1AX, United Kingdom; ^*2*^*Provincial Department of Indigenous Medicine, Trincomalee, Sri Lanka*

###### **Correspondence:** Saravanan Sathasivampillai (svsathasivampillai@yahoo.com)

**Background**

Diabetes is one of the health problems affecting the economic and social developments in countries like Sri Lanka. Siddha (Tamil) Medicine is practised typically in the Eastern and Northern Provinces of Sri Lanka. Plants are the most frequently used ingredient in Siddha Medicine. A recent review of historical and modern textbooks revealed 171 plant species recorded for treating diabetes in Sri Lankan Siddha Medicine [1]. However, there is no record of the current use of antidiabetic plants. Thus the aim of this study is to understand the importance of Siddha healers (Eastern Province) for patients suffering from diabetes and to document the plants presently used to treat diabetes in the region.

**Material and methods**

Between 1st July and 1st September, 2016 an ethnobotanical survey was conducted including interviews with 27 Siddha healers living in Eastern Province in Sri Lanka to identify and document the plant species currently used to treat diabetes.

**Results**

The majority of the Siddha healers interviewed were male with an average age of 60 years and 40 years of experience in practising Siddha Medicine. Overall, 90 plant species from 47 families were recorded with *Syzygium cumini* (L.) Skeels being the most frequently reported species. One third of the species are not listed in our previous review including the globally used plants such as *Catharanthus roseus* (L.) G.Don. Fabaceae is the family yielding the largest number of plants species used. The majority of the plants recorded are food plants including grains, green leaves (usually consumed as vegetable dishes in Sri Lanka), spices, fruits, and weeds. Interestingly, animal parts and inorganic substances mentioned in the antidiabetic preparations in textbooks [1] currently are not used to prepare antidiabetic preparations. Moreover, only oral preparations are presently prescribed. Consultation of practitioners seems to be widespread and taken together among the 27 healers on average 325 diabetic were seen per week. Furthermore, the majority of the diabetic cases are diagnosed by combining pulse reading (one of the eight Siddha diagnosis methods) and verifying Siddha diabetic symptoms.

**Conclusions**

Consultation of Siddha healers and the subsequent use of herbal medicines is an important element of health care practice in the Eastern Province. This study provides a foundation for further understanding this usage, and for developing more integrative approaches in this rural and poor region, which, however, can only be a long term objective.

**Reference**

1. Sathasivampillai VS, Rajamanoharan, P, Munday, M, Heinrich, M. Plants used to treat diabetes in Sri Lankan Siddha Medicine – An ethnopharmacological review of historical and modern sources. J Ethnopharmacol. 2016; In press.

### P306 Traditional healing systems: A framework approach against the backdrop of sustainable development

#### Yvonne M Scherrer^1^, Michael Heinrich^2^

##### ^1^ Sustainability Research Group, University of Basel, Basel, 4051, Switzerland; ^*2*^*Research Group 'Pharmacognosy and Phytotherapy', Univ. London, School of Pharmacy, London, WC1N 1AX, United Kingdom*

###### **Correspondence:** Yvonne M Scherrer (yvonne.scherrer@unibas.ch)

**Question**

Starting from the premise that science has for too long been bound by disciplinary boundaries, this contribution outlines the conceptual similarities between integrative medicine and sustainable development (SD). It argues for taking advantage of this concurrence by suggesting a structured approach to understanding traditional healing systems (THS) through the lens of SD in the sense of a more holistic practice of medicine. Examining THS against the backdrop of SD shows that most bioscientific studies on THS restrict themselves to largely functional aims by scrutinizing isolated knowledge items to determine their applicability for allopathic medicine whereas comparatively few studies research THS in their own right.

**Methods**

Methodologically, an extended literature analysis determined the central contributions by anthropology, philosophy and sociology regarding understanding traditional knowledge. Subsequently, suitable generic dimensions were distilled and aggregated in a conceptual-analytical framework following Jabareen[1], Dowdig [2] and Stanley[3] and considering the normative principles of SD.

**Results**

The result consists in a generic framework on THS based on theories from philosophy, sociology and anthropology, consistent with SD and intended to facilitate access to principally any given traditional knowledge form by providing a set of leading dimensions and questions. Next to covering knowledge content and processes/skills, the framework also includes central complementing dimensions such as the knowledges social organization, its socially legitimized sources or the general life-world context.

**Conclusions**

A structured and social sciences-based approach to THS supports researchers and professionals from all disciplinary backgrounds in gaining a comprehensive understanding of a given THS with the aim of making the insights viable for advancing holistic approaches to medicine in the West.

**References**

1. Jabareen, Yosef R. Building a Conceptual Framework: Philosophy, Definitions, and Procedure. International Journal of Qualitative Methods. 2009; 8:49–62.

2. Dowding, Keith. There Must Be End to Confusion: Policy Networks, Intellectual Fatigue, and the Need for Political Science Methods Courses in British Universities. Political Studies. 2001; 49:89–105.

3. Stanley, Liam. The Difference Between an Analytical Framework and a Theoretical Claim: A Reply to Martin Carstensen. Political Studies. 2012; 60:474–82.

### P307 My customers put trust in me: Gender roles, traditional medicine, and healing processes in Indonesia

#### Carolyn Szuter

##### Sociology, University of New Brunswick, Fredericton, E3B4H7, Canada

**Purpose**

Jamu, a Javanese traditional medicinal drink consisting of local plant ingredients, is produced and consumed in Indonesia, both in the rural and urban areas, to treat a variety of diseases, including diabetes, cold, stomach ache, and a number of womens reproductive health issues. Jamu is mainly prepared and sold by local women. The study analyzes how *jamu* sellers perceive their role in the healing process of customers and their perceptions about jamu efficacy.

**Methods**

This study is based on an ethnographic study conducted in the city of Yogyakarta, Indonesia, where individual in-depth interviews were conducted with a group of 91 *jamu* sellers.

**Results**

According to *jamu* sellers, the healing process depends on the active ingredients of the drink itself and the capacity of the *jamu* seller to select and mix them. Interviewees emphasized how the reputation of the *jamu seller* plays a central role in determining the perceived efficacy of *jamu* by the customers. J*amu* sellers recognize that they must appear physically healthy, which is attributed to an effective *jamu* recipe. The reputation of jamu is also linked to specific behavioural traits of the jamu seller, including smiling, appearing friendly, and being generous.

**Conclusion**

The concept of efficacy of traditional medicine is socially constructed, depending on the reputation of the jamu sellers, and inscribed into the existing gender roles of Javanese society, which places particular importance on specific physical and behavioural traits of women.

### P308 Therapeutic effect of marshmallow in Traditional Persian Medicine, a review article

#### Fatemeh Amini, Fatemeh Tabatabaei

##### School of Medicine, Traditional Medicine, Shiraz, Iran, Islamic Republic of

###### **Correspondence:** Fatemeh Amini (amini.bahar@gmail.com)

**Purpose**

Marshmallow or althaea officinalis (grown in Iran) or alcea officinalis (grown abroad of Iran) is one of the important simple drug in traditional Persian medicine (TPM). Many therapeutic effects were listed in TPM manuscript for marshmallow. Not only many of these effects were proved in classic medicine but also no significant side effect already were reaported. Some of proved effects of marshmallow were mentioned in TPM books and a lot of them are yet unproved

**Methods**

At the beginning of the study, TPM pharmacopeia references like “Makhza-al-advyeh” and “tohfeh” and books of treatment like “Teb e akbari” and “ExireAazam” were searched for determination of marshmallow benefits. Then English and Persian databases including PubMed, web of science, Scopus, google scholar, cochrain library, SID, Iran medex, Magiran for effects of marshmallow and the results were compared.

**Conclusion**

Using of marshmallow as an effective and useful drug can be considered also further research for other therapeutic effects of it can be useful.

### P309 Tinnitus, treatment from the viewpoint of Traditional Persian Medicine

#### Ali Tavakoli, Fatemeh Tavakoli, Mehdi Pasalar, Mahsa rostami

##### Research Center for Traditional Medicine and History of Medicine, Shiraz University of Medical Sciences, Shiraz, Iran, Islamic Republic of

###### **Correspondence:** Ali Tavakoli (tavakkolia@sums.ac.ir)

**Introduction**

Chronic tinnitus is a world-wide problem of high prevalence (5% and 15%) and socio-economic relevance that considerably impaired quality of life. While many different treatments are used in clinical practice, the evidence for their efficacy is low and the variance of treatment response between individuals is high. So there are other treatments required. This disease has been mentioned in ear diseases section of Traditional Persian Medicine (TPM) manuscripts and for it is considered causes, clinical symptoms and treatments according to principles of this doctrine of medicine. The aim of this study is to introduce the viewpoint of TPM scholars for the treatments of tinnitus.

**Methods**

TPM manuscripts like Canon were investigated for tinnitus and its treatments.

**Results**

*Tanin* is defined as the hearing of sound in the absence of a external acoustic stimulus. It is classified into two subgroup. 1- Voices that generated in the body and not heard in healthy people be heard to form of *Tanin*. 2- There is a stimulant factor inside the head and brain or ear that cause *Tanin*. Subgroup 2 is sever and louder than subgroup 1. *Tanin* with this definition and classification is similar to tinnitus in modern medicine.

Causes, symptoms and treatments of *Tanin* (chronic tinnitus): 1-If cause of tinnitus is severe weight loss or lack of food intake for long time or severe illness that weakens the powers of the body, including hearing, treatments included Increase the volume and number of servings of food, eating of foods with quick digestion and cold nature and soft like water of meat and reinforcement brain and ear with rose oil drop or violet oil or violet almond oil. 2- If cause of tinnitus is accumulation of waste in the head or ears, with symptoms such as feeling of heaviness and tension in the head and ears, treatments included consumption of Jallab (mixture) from Anise and Fennel and Licorice with Golghand, nebulized with decoction steam of Wild Mint and Marjoram and Thyme (*Thymus vulgaris*) then reinforcement the ear with Lily (*Lilium ledebouri*i) oil drop with continuous bath. 3- If cause of tinnitus is overload of blood humor in blood vessels of head and body (*Emtella*), treatment is venesection (remove part of extra blood from certain blood vessel of the body).

In all type of tinnitus the patient should be avoided from sitting in the sun, near the fire, very hot bath, strenuous physical activity, loud voice, too much talking, frequent Sexual intercourse, overeating, consumption of Garlic (Allium Sativum) or Onion (Allium cepa L) or Chives (Allium schoenoprasu) or wine, long time Starvation, sleeping with full stomach and constipation.

**Conclusions**

To prove these practices of TPM sages for chronic tinnitus more researches are necessary to be performed.

Keywords: Tinnitus, Traditional Persian Medicine, Treatment

### P310 Use of traditional medicine for maternal and child health care among women in urban Indonesia: The Jamu system and its practices in the city of Jagyakarta

#### Maria C Torri, Carolyn Szuter

##### Sociology, University of New Brunswick, Fredericton, E3B4H7, Canada

###### **Correspondence:** Maria C Torri (mctorri@yahoo.it)

**Purpose**

Jamu is an Indonesian traditional herbal medicine, based on plants and roots, which has been used for many centuries in the Indonesian community to maintain good health and to treat diseases. Although biomedicine is becoming increasingly important in Indonesia, jamu is still very popular in rural as well as in urban areas. This paper presents findings on knowledge, attitudes, and practices regarding jamu for maternal and child health care among both consumers and jamu small producers in Indonesia.

**Methods**

In-depth interviews were carried out with 35 women and 91 jamu producers in Yogyakarta, Java. The interviews consisted of semi-structured questions (duration 45 minutes to 1 hour) in which general information was gathered about the different uses and perceptions of *jamu* and traditional medicine, as well as their reputed therapeutic effects and socio-cultural values.

**Results**

Results show that different types of jamu are used for different purposes in various stages of a woman's life including for conditions associated with menstruation, puberty, pregnancy, abortion, birth, postnatal health and breastfeeding. Our study reveals that due to their educational and social background, the women have divergent ideas about the use of jamu. Attitudes towards the use of jamu are also influenced by generational factors.

**Conclusion**

The patterns of taking *jamu* deserve attention, since many are consumed regularly as preventive medicine. Study of womens’ practices related to maternal and child health care is crucial to the design and implementation of effective health programmes in urban areas.

### P311 How does RegentK work? A qualitative-phenomenological investigation into a novel complementary-chiropractic technique

#### Harald Walach (walach@europa-uni.de)

##### Pediatric Gastroenterology, Poznan Medical University, Poznan, 60-572, Poland

**Question**

Mohammed Khalifa is a well known Austrian chiropracter or manipulative therapist who has treated world level athletes with his self-developed technique called RegentK (regenerative technique according to Khalifa) such that they are able, even after severe injuries such as cruciate ligament ruptures or luxations, to continue performing after just one therapeutic sessions. A clinical trial with imaging has shown that one treatment improves knee function to near full functioning after cruciate ligament rupture and stimulates complete end to end healing of ligaments in 8 out of 15 cases. This self-experiential phenomenological study was conducted in order to better understand how the process works, as the explanatory framework of Khalifa himself is not adequate.

**Methods**

The author underwent a treatment himself as a self-experience and immediately afterwards produced a rich qualitative experiential narrative. In addition he queried Khalifa about his own theories regarding his technique and read through materials that journalists had produced after interviewing Khalifa.

**Results**

RegentK is a technique where the therapist uses very low frequency pressures of an intense kind on fascial spots and trigger points distal to an injury and works his way upwards. In his own terminology these are "zero-points", where the tissue answers to his probing pushes and grips, responding, as it were, to a stimulation for growth. In first-person experiential terms these points are extremely painful and it feels as if the therapist were actually becoming one with the tissue. The time experience is extremely distorted during this 45 minute treatment. After feeding back the experience to the therapist, he actually agreed. He said that indeed he was "changing time" in his own experience.

**Conclusions**

The interpretative framework and the phenomenology of first-person-experience of RegentK therapy seems to suggest that during this treatment the therapist somehow, in addition to all traditional efforts at stimulation, such as introducing potentially piezo-electric effects, changes the joint experienced time frame and enters the boundaries of another body in order to stimulate self healing. This can be conceptualised within the framework of a non-local theory.

### P312 An investigation in the correlation between Ayurvedic body-constitution and food-taste preference

#### Faith Warner^1^, Anne Majumdar^2^, Palitha Serasingh^1^

##### ^1^ Middlesex University, London, NW4 4BT, United Kingdom; ^*2*^*SHAS, St Mary's University Twickenham, Twickenham, TW1 4SX, United Kingdom*

###### **Correspondence:** Faith Warner (FW148@live.mdx.ac.uk)

**Background**

Poor diet is a key modifiable risk-factor for chronic disease. Ayurvedic nutrition offers a personalised perspective of disease prevention and management. Environmental factors are associated with poor dietary choices but there is little extant literature on internal cues.

**Aims**

The aim of this research was to identify whether a relationship exists between Ayurvedic constitution (prakriti) and food taste preference and food choice.

**Method**

This study involved thedevelopment and implementation of a comprehensive Ayurvedic Constitutional Analysis Tool (A.C.A.T) and Food-taste Preference Analysis Tool (F.P.A.T). Each tool was piloted. Participants, recruited via social media, were screened via inclusion and exclusion criteria. Suitable participants (*n* = 25) were asked to complete both tools. Appropriate demographic and inferential statistics were used to analyse the data. Ethical approval was granted by Middlesex University.

**Results**

Findings from the study and statistical non-parametric analysis suggest a perceptible relationship between traditional Ayurvedic body-constitution and food-taste preference. It was found that those of kapha, pitta-kapha and tridoshic constitution had a tendency to prefer sweet and pungent while pitta types were found to have a strong distaste for salt, pungent and bitter. Vata types had preferences for sour and bitter, compared to the other constitutions.

**Discussion & Conclusions**

Findings from the study suggest that the constitution may play a vital role in taste preference and food selection with potential positive and negative implications on health. Further investigation in this area is warranted and would contribute to an evidence-basis for Ayurvedic dietetic principles and a framework with which to practice personalised nutrition based on constitution.

### P313 Buzhongyiqi decoction for myasthenia gravis: A systematic review of randomized controlled trial

#### Xiaoyi Yan, Liyan Jia, Nanqi Zhao, Zhaolan Liu, Jianping Liu

##### Center for Evidence-based Chinese Medicine, Beijing University of Chinese Medicine, Beijing, China

###### **Correspondence:** Zhaolan Liu (lzl1019@163.com)

**Purpose**

To assess evidence of Buzhongyiqi decoction for myasthenia gravis (MG) from randomized clinical trials (RCT).

**Method**

Six databases were searched through November 2016 for RCTs on Buzhongyiqi decoction for MG. Two authors independently selected literature, extracted data and assessed the risk of bias using the Cochranes tool. We performed data analysis using RevMan 5.3.

**Results**

Eleven RCTs involving 663 participants were selected. Overall risk of bias was unclear. Two trials compared Buzhongyiqi decoction with pyridostigmine bromide. One trial (*n* = 57) observed AchRab, IgG, IgM, IgA and C3 which all showed no significant difference; significant effect showed in the effective rate I (defined by symptoms and signs remission) (OR 0.72, 95% CI 0.11 to 4.67). Other one (*n* = 40) assessed effective rate II (defined as 25% increasing of relative clinical score) showed no significant difference. Two trials (*n* = 80) compared Buzhongyiqi decoction with prednisone showed significant difference in the effective rate I (OR 1.57, 95% CI 0.53 to 4.66; I2 = 0%). Two trials (*n* = 212) compared Buzhongyiqi decoction plus prednisone with prednisone which showed significant difference in the effective rate I (OR 2.98, 95% CI1.58 to 5.63; I2 = 0%). Five trials compared Buzhongyiqi decoction plus prednisone plus pyridostigmine bromide with prednisone plus pyridostigmine bromide. Two trials (*n* = 108) reported effective rate I which showed significant difference (OR 6.78, 95% CI 2.21 to 21.67; I2 = 0%).

**Conclusion**

Buzhongyiqi decoction may have an effect on symptoms remission in patients with MG, but the function of immunological regulation is not obvious. The methodological quality of studies is poor. Further rigorously designed studies are needed.

### P314 Characteristic of RCT of fire needle as a treatment for herpes zoster

#### Nanqi Zhao^1^, Fei Zhen^2^, Liyan Jia^1^, Xiaoyi Yan^1^, Zhaolan Liu^1^, Jianping Liu^1^

##### ^1^ Evidence-based medicine center, Beijing University of Chinese Medicine, Beijing, China; ^2^ Dongfang Hospital, Beijing University of Chinese Medicine, Beijing, China

###### **Correspondence:** Nanqi Zhao (silent1280@163.com)

**Objective**

To analyze characteristic of fire needle treating herpes zoster RCT.

**Methods**

We searched the Chinese Biomedical Literature Database (SinoMed), China National Knowledge Infrastructure (CNKI), Chinese VIP Information (VIP), Chinese Academic Conference Papers Database and Chinese Dissertation Database (Wanfang), PubMed, Cochrane Central Register of Controlled Trials (CENTRAL). Retrieval date was of September 1st, 2016. We chose patients with herpes zoster in acute or sequelae stage as research object and the intervention is fire needle. The outcome measures include herpes healing time, degree of pain relief. Two researchers worked independently to screen the literature, extract information and evaluate methodology quality.

**Results**

We included 24 studies, with 1698 participants. They are all Chinese literatures. It also includes 10 kinds of comparision: Western medicine + fire needle Vs. Western medicine + ordinary acupuncture, Chinese herbal medicine + fire needle Vs. Chinese herbal medicine, fire needle Vs. ordinary acupuncture, fire needle Vs. Western medicine, Western medicine + fire needle Vs. Western medicine, acupuncture + moxibustion Vs. moxibustion,Conventional treament + fire needle Vs.Conventional treament, fire needle Vs. electro-acupuncture, fire needle Vs. fire needle + electro-acupuncture, fire needle Vs. external use drugs. Risk bias assessment: 13 trials reported random sequence generation, including random number table method, central randomized. 5 trails used allocation conceal: including sealed envelope method, concealed by the center of the national drug clinical research (Chengdu University of Traditional Chinese Medicine GCP center). No trail used blinding of participants and personnel. 1 trail used blinding of outcome assessment. 4 trails have bias with incomplete outcome data. There was no bias with selective reporting. 7 trails were funded. 1 trail is low risk of bias, the other 23 is high risk of bias.

**Conclusion**

Fire needle treatment of herpes zoster has been recognized by Chinese patients, with clinical effect, cheap price, and strong operability. Due to lack of high quality clinical trials as evidence-based, it carries more difficult for standardized clinical treatment of fire needle.

## MEDICINE AND ARTS

### P315 The adjustment of the CARE guideline and the development of a documentation method, to be used in anthroposophic art therapy

#### Annemarie Abbing, Anne Ponstein, Erik Baars

##### Anthroposphic Health Care, University of applied sciences, Leiden, 2300 AJ, Netherlands

###### **Correspondence:** Annemarie Abbing (abbing.a@hsleiden.nl)

**Purpose**

Anthroposophic art therapy (AAT) is facing problems due to a lack of evidence and the lack of generally accepted concepts of the working mechanisms. Case reports can provide insight in working mechanisms and allow for describing the unique interaction between therapist and patient. Multiple case reports of academic quality will also allow for the identification of best practices, which may serve as a starting point for efficacy studies.

The CARE (CAse REport) guideline was developed in 2013 to improve the quality of medical case reports. The guideline was subsequently adjusted into a specific guideline for AAT. The academic, non-chronical order of this CARE-AAT guideline (2016) appeared to limit use of the guideline in daily practice by art therapists. It was envisioned that a documentation method could support therapists in the systematic gathering of all information necessary for a complete, transparent and comprehensible case report.

Methods

A documentation method was developed by means of expert knowledge and literature. The face validity and usability of the concept method was determined through a survey. In a field test, dossiers were collected and subsequently analyzed for completeness and quality with the CARE-AAT guideline as a reference.

**Results**

The survey provedthat the face validity of the documentation method was good. The completeness and quality of the provided dossiers differed considerably. Some dossiers lacked crucial information, obstructing academic case reporting.

**Conclusions**

These findings have led to the further improvement of the documentation method. The guideline and the improved documentation method will be presented.

### P316 Proposing "Theatricality" as a novel, holistic methodology for the study of complex interventions in CAM

#### Sarah Croke (dr.sarah.croke@hotmail.com)

##### School of Heathcare, University of Leeds Alumnus, Rochdale, OL12 7RX, United Kingdom

**Purpose**

Theatricality methodology is proposed as a novel approach to explore gaps and enhance the research design and reporting in Complementary and Alternative Medicine (CAM). Use of a theatre concept provides a unique potential, as both CAM and Theatre represent global worlds where everything inside is a vital part of the whole event and, as a framework for researchers, this approach offers the potential to engage creatively and holistically with the whole complexity of CAM practice, it's characters and setting.

**Methods**

The approach was trialled in a study of 5 clinics across 4 European countries, with 31 practitioners and 47 patients actively taking part. Theatricality Framework Method (TFM) captured the scenography of each setting, while participant observations reported the actions and in-depth interviews created the dialogue. The researcher's responses completed the data set, which was viewed and analysed via 'performative criticism' with embodied reflexivity.

**Results**

This approach created a holistic 'space' where CAM practices could be perceived, engaged and reported as complex, therapeutic, healing events. Findings from this suggested that primary targets of therapy were often people, not problems, and that quality of outcomescould be determined by the whole contextual blend of practitioners, interventions and place. The research processwas similarly affected, finding it easier to engage holistically where participants acted holistically, and more difficult where they did not.

**Conclusion**

Theatricality proved effective and innovative for engaging 'the whole and the parts' of therapy together and extracting complex meanings. This suggests a potential new quality for research in CAM.

### P317 Evidence-based music therapy protocols in integrative medicine and health

#### Suzanne Hanser (shanser@berklee.edu)

##### Music Therapy, Berklee College of Music, Boston, 02215, United States

The evidence base for music therapy interventions is growing as this service evolves into a more accepted form of integrative medicine and health. This investigator has developed such music therapy protocols for medical patients, and tested their effectiveness in randomized controlled trials, 3 of which are presented here.

The music therapy protocol for a family medicine unit involved an assessment of musical preferences and background, as patients sampled receptive and active musical experiences. The music therapist prepared individual music playlists, and met individually at bedside to demonstrate music-facilitated guided imagery, meditation, relaxation and breathing techniques. More active interventions included songwriting, improvisation, singing/chanting, playing instruments, lyric substitution, and lyric analysis. Results no significant differences between treatment and usual care groups, but post-treatment interviews revealed positive outcomes in relaxation and the patient experience overall.

Research with women who had metastatic breast cancer engaged these women during their chemotherapy treatment in the infusion unit of a major cancer center. They participated in relaxation exercises facilitated by live music, active improvisation on percussion instruments, and songwriting. Outcomes revealed post-session decreases in heart rate and positive changes in reported levels of anxiety, pain, and contentment. A set of eight music-facilitated stress management strategies were tested with depressed older adults. These included music listening techniques designed to relax the body and mind. Results included clinically significant changes in depression, anxiety, mood and distress, and statistically significant differences on multiple measures between music therapy participants and a no-contact wait list control group.

### P318 Changes in respiration, heartrate and state of mind while hearing live music: An empirical experimental pilot study in Anthroposophic Music Therapy

#### Viola Heckel^1^, Daniel Krüerke^1^, Ana P Simões-Wüst^1^, Sebastian Weiss^2^, Susanne Metzner^3^

##### ^1^ Klinik Arlesheim, Arlesheim, 4144, Switzerland; ^*2*^*Klinik Öschelbronn, Niefern-Öschelbronn, Germany;*^*3*^*University of Augsburg, Augsburg, Germany*

###### **Correspondence:** Viola Heckel (viola.heckel@gmx.de)

**Introduction**

Music therapeutic interventions are used to positively change patients’ emotions and mood, which influence related physiological parameters. During therapeutic receptive lyre interventions in conjunction with singing, alterations in respiration and mood perception have been observed in oncological patients [1]. We determined changes of respiration rate (RR), heart rate variability (HRV) and mood during the same therapeutic intervention as used for oncologic patients, in six healthy volunteers.

**Methods**

Quantitative and qualitative evaluation with mood questionnaire [2] and semi structured interviews based on method of Qualitative Contents Analysis and Grounded Theory before and after each session. HRV and RR measurement was performed during pre-rest period, intervention [3] and post-rest period, using an ECG recorder with respiration sensor (MK3, TOM Medical). SimpleView© (Version 2.2, Release 15, TOM Medical) and MatLab© programming was used for analysis of HRV.

**Results**

After intervention: reduced heart rate, increased HRV, more regularly respiration, higher cardiorespiratory coordination and a shift towards higher vagal activity were found. Those changes remain during post-rest. Physiological results are accompanied by improvements of inner balance and vitality.

**Discussion**

Methods used for data collection maybe used in a controlled clinical study. Physiological and psychological outcome give evidence for sample size estimations and for power calculation.

Keywords: lyre intervention, cardiorespiratory coordination, mood

**References**

1. Heckel V. (2015) Veränderungen der Atmung bei live-gespielter Musik, Masterthesis, HS Magdeburg – Stendal.

2. Hobi V. (1985) Basler Befindlichkeits-Skala. Weinheim: Beltz-Test.

3. Noten: SchüppelM. (1960) Merkurbadˮ Therapiemusik 808 Edition Mensch und Musik. © 1998 Musiktherapeutische Arbeitsstätte e. V. Berlin

### P319 The herbal formula Galgeun-tang (Gegen-decotion) versus western medicine for treating cervical spondylosis: A systematic review of randomized clinical trials

#### Jang W Lee^1^, Min K Hyun^2^

##### ^1^ College of Korean Medicine, Dongguk University, Goyang, South Korea^*2*^*Preventive Medicine, College of Korean Medicine, Dongguk University, Gyeongju, South Korea*

###### **Correspondence:** Jang W Lee (wkwk0123@gmail.com)

**Objective**

We evaluated efficacy and safety of Galgeun-tang (Gegen-decotion) versus western medicine for treating cervical spondylosis in the randomized controlled studies RCTs.

**Methods**

We systematically searched 10 databases including PubMed; Cochrane Library; EMBASE; two Chinese medical databases (China National Knowledge, Airiti Library); one Japanese medical database (CiNii); and four Korean medical databases (OASIS, KoreaMed, KMBASE, NDSL) until January 2017. There were not any language restrictions for randomized controlled trials (RCTs) comparing Galgeun-tang with western medicine (Celecoxib, Brufen, Ibuprofen, Mecobalamin). Data extraction and risk of bias assessments performed by two independent reviewers. Response rate was evaluated by 'Criteria of diagnosis and therapeutic effects of diseases and syndromes in traditional Chinese medicine'. The quality of the RCTs was assessed using the Cochrane risk of bias tool.

**Results**

Finally, four RCTs with a total of 552 participants were included in the analysis. The methodological qualities of the RCTs were low in the performance bias domain. The Galgeun-tang group had superiority in the response rate compared with Celecoxib group in two RCTs eligible for the meta-analysis (*n* = 338, RR:1.17, 95%CI : 1.09-1.25, *P* < 0.0001, I^2^ = 3%). The other two RCT also showed a favorable effect of Galgeun-tang on response rate compared with western medicine.

**Conclusion**

Galgeun-tang may have potential effects on cervical spondylosis compared with western medicine. However, these RCTs have moderate and low quality of evidence. Therefore, the results need to be confirm in rigorous and large RCTs.

### P320 Medicine and art: The effects of professional clowns performance at pediatric hospitals

#### Morgana Masetti (morgana.ops@terra.com.br)

##### Psychology, Pontificia Universidade Católica de Sao Paulo, Rio de Janeiro, 22430, Brazil

The current work aims to present the results of research made on the influence of the performance of clowns from the NGO Doutores da Alegria (professional artists working in pediatric hospitals in Brazil since 1991) on the hospitalized children, parents, caregivers and health professionals. The study had the following methodology:

1. Qualitative phase: 12 focus groups with health professionals carried out every 6 months for 3 years at 2 hospitals in order to map performance indicators of the clowns acting over this period.
2. Quantitative phase: Implementation of structured questionnaires answered by 567 health professionals in 13 hospitals where Doutores da Alegria act.

Some results about the clowns effects that stand out in this study: In the perception of most health professionals, children became more comfortable with the hospital environment, more active and collaborative with the team. Children accept better medical examinations and procedures after the clowns visit. Professionals develop new ways to approach the children and started to talk more with them. They say that the families started to play more with the children and become more collaborative with the heath professional team. Most professionals agree that they feel calmer with the work and perform the work routines with better quality.

The results show that the performance of clowns with the hospitalized children develops a better quality in relations between health professionals, patients and families.

### P321 *…To take medicine into the picture …* art therapy and well-being – a pilot study in prevention and health promotion

#### Renate Oepen^1^, Harald Gruber^1^, Peter Heusser^2^

##### ^1^ Research Institute for Creative Arts Therapies (RIArT), Alanus University of Arts and Social Sciences, Alfter/Bonn, 53347, Germany; ^*2*^*Theory of Medicine, Integrative and Anthroposophic Medicine, Witten/Herdecke University, Herdecke, 58313, Germany*

###### **Correspondence:** Renate Oepen (renate.oepen@alanus.edu)

**Purpose**

This pilot study examined the effectiveness of an art therapy intervention concept on current and habitual well-being of stressed Waldorf School teachers. Furthermore, we identified potential work factors of art therapy that could be connected with the positive outcome.

**Methods**

The specialised concept of four art therapy interventions was applied in a single day project with 18 teachers (15 f, 3 m; mean age: 48). The evaluation was conducted using quantitative and qualitative methods. In the quantitative analysis, the change of habitual well-being was assessed with the SF-36 Health Survey, the change of current well-being with the Complaint List (B-L) and the Scale of Current Mood (ASTS). The qualitative analysis aimed to generate art therapeutic work factors. It was carried out by the structured content analysis of Mayring. These data were obtained on the basis of two interviews, conducted about two and five weeks after the project day, with selected subjects.

**Results**

The quantitative analysis concerning current well-being indicated a significant increase of positive mood and a significant reduction of complaints. In examining the habitual well-being, a short-term improvement could be observed. In the qualitative analysis three common and seven specific art therapy factors could be identified and be related to the positive outcome, namely the common factor Advancement of Cognitive Processes/Support of Development of Coping Strategies and the specific art therapy work factor Stimulation of Symbolization and Imagination.

**Conclusions**

Resource-oriented, art therapy interventions may be effective in stress prevention and health-promotion in teachers with stress.

### P322 Palpation of inherent rhythms in Osteopathic Medicine: Physiology meeting practice

#### Holger Pelz, Volker Perlitz

##### DGOM e.V./HGMB i.G., Buxtehude, 21614, Germany

###### **Correspondence:** Volker Perlitz (perlitz@simplana.de)

**Objective**

Principles of Osteopathic Medicine (OM) rest on a hypothesis put forth 150 years ago by William Sutherland on the mechanical origin of inherent rhythms (IR). This paucity of theory is contrasted by a wealth of practical observations of scores of practitioners the world over on manual palpation of e. g. the Cranio-Rhythmic Impulse (CRI) as a prominent IR. Recently a novel hypothesis was introduced identifying striking parallels in dynamics between the CRI and a 0.15 Hz-rhythm band (RB) which was demonstrated to govern in multiple peripheral physiological time series in human volunteers and anesthetized dogs.

**Methods & Results**

Using nonlinear algorithms, dynamics were detailed in RB, and elaborated manual techniques (e.g. vault hold) utilizing anatomical mechanical receptors to palpate CRI by 0 M practitioners. Either technique exhibited period lengths of 7-11 oscillations/minute as one of the most essential features of either of these rhythms. Another feature is spread of the RB with arterial systemic blood pressure across the entire organism as a correlate of the CRI. Due to differences of dynamics of sympathetic and parasympathetic nervous traffic, disruptions of rhythmic manifestations may become palpable as somatic dysfunctions, while rhythmic transfer from therapist to patient may yield correction thereof.

**Conclusions**

Using objective and individual (= subjective) levels of observation suggests put forth a robust hypothesis on essentials of RB or CRI which can be utilized as therapy regimes.

### P323 Application of the CARE-AAT documentation method in anthroposophic art therapy case reporting

#### Anne Ponstein, Annemarie Abbing, Erik Baars

##### Anthroposphic Health Care, University of applied sciences, Leiden, 2300 AJ, Netherlands

###### **Correspondence:** Anne Ponstein (ponstein.a@hsleiden.nl)

**Purpose**

It is envisioned that case reporting is a good way of providing insight in the working mechanisms underlying art therapy, as it leaves the uniqueness of the interaction between therapist, medium and patient intact.

**Methods**

In a field test ten dossiers were prospectively collected employing the CARE-AAT documentation method. One dossier was selected based on completeness and used as the starting point for writing a case report in which the working mechanism of art therapy was explored. The problems met in the process of clarifying the working mechanism were discussed in a team of art therapists with a research background.

**Results**

The most complete dossier was used as the starting point of academic case report writing. Dossier information was successfully sorted into the categories of the CARE-AAT guideline. Then it was decided which message to bring across by case reporting. This led to a clear focus during additional data collection (from literature) and data exclusion (from the dossier). Several ways to enhance the academic quality of clarifying the working mechanism of art therapy like predictability, causality and validity, were addressed.

**Conclusion**

The CARE-AAT documentation method was successfully used in prospective dossier formation. Several ways of demonstrating the working mechanism of art therapy in case report writing based on the dossier were identified and led to some changes in the documentation method.

### P324 The potential of Tai Chi for long term health: Exercise preferences for people with cystic fibrosis

#### Nicola Robinson^1^, Patricia Ronan^1^, Awais Mian^1^, Su Madge^2^, Ava Lorenc^1^, Penny Agent^2^, Siobhan Carr^2^

##### ^1^ School of Health and Social Care, London South Bank University, London, SE1 0AA, United Kingdom; ^*2*^*Royal Brompton and Harefield NHS Trust, London, United Kingdom*

###### **Correspondence:** Nicola Robinson (nicky.robinson@lsbu.ac.uk)

**Introduction**

Tai Chi (TC) is normally practised standing, and can be practised seated, alone, or with friends/family. People with cystic fibrosis (CF) are often confined to home or hospital, and their daily exercise, which is an essential component of treatment, is often comprised due to ill health. TC, a gentle, more manageable exercise, may offer a solution to people with CF.

**Methods**

Children and adults attending CF clinics were invited to participate in a randomised trial offering eight TC lessons either face to face (group 1) or over the internet (group 2). Recruitment data was collected about why people did not wish to join the study. Participants were asked about their regular TC practice after lessons and 20 participants were asked in interviews 2 months after the end of lessons.

**Results**

Of 116 outpatients approached, 65 people declined, 51 accepted and 40 completed all lessons. 43% of those who refused did not like the idea of TC, 36% were too busy. Of those who participated, all practiced between lessons on average 3 times weekly for 13 minutes. At follow up 84% interviewed, still practised an average 2/3 times/week (group 1 = 62.5% vs group 2 = 37.5%). 79% of respondents said they intended to continue TC, (group 1 = 53% vs group 2 = 47%). Some participants asked about local classes. 32%, all children, included others in lessons. Two participants reported using TC whilst hospitalised.

**Conclusions**

TC could prove to be an alternative exercise for people with CF that could be integrated into a weekly exercise regime.

### P325 Benefits for people with cystic fibrosis learning Tai Chi: Comparison of self-reported to objective data in a non-acute/community population

#### Patricia Ronan^1^, Nicola Robinson^1^, Siobhan Carr^2^, Awais Mian^1^, Ava Lorenc^1^, Penny Agent^2^, Su Madge^2^

##### ^1^ School of Health and Social Care, London South Bank University, London, SE1 0AA, United Kingdom; ^*2*^*Royal Brompton and Harefield NHS Trust, London, United Kingdom*

###### **Correspondence:** Patricia Ronan (ronanp@lsbu.ac.uk)

**Introduction**

A strict regime of physiotherapy and regular exercise is an important part of daily regime to maintain the health of people with cystic fibrosis (CF). A mixed methods approach was utilised to collect data in a randomised feasibility study to explore the physical and psychological benefits of including Tai chi (TC) alongside normal exercise.

**Methods**

Validated questionnaires, objective clinical data, qualitative interviewing and self-reported measures were employed to gather data on a range of potential outcomes, including breathing, gastric pain, sleep, anxiety, posture and fitness. Learning TC face to face was compared with no TC and learning TC over an internet connection.

**Results**

Forty participants were recruited: 23 had face-to-face TC lessons (group1), whilst 17 had no TC lessons for 3 months, and then had TC lessons delivered over the internet (group2). Quantitative data showed trends in improvements, but no statistical significance. In qualitative interviews and questionnaires, 58%of participants reported improvements in breathing (group 1 = 70%, group 2 = 30%), including being able to take larger breaths, breathe better at night, and improved expectoration (45%). Improved posture (45%), and feeling calmer and less stressed (90%) were also reported.

**Conclusions**

People with CF may find TC helpful to reduce stress, improve breathing, posture and other issues associated with CF. However, findings are not supported by the recognised objective data collection tools. Such tools may not be sensitive to minor changes in a relatively well population. Further research is required to ascertain whether such improvements occur and how to improve outcomes using internet delivery.

### P326 Medical Painting Therapy "Methodology Liane Collot d'Herbois" – A therapeutic process of Anthroposophical Medicine

#### Monica E Winnubst, Ricardo Monezi

##### UNIFESP, Sao Paulo, 04753-060, Brazil

###### **Correspondence:** Monica E Winnubst (monica@atelierpedraemetal.com.br)

**Question**

To present the Medical Painting Therapy in Liane Collot d'Herbois methodology, an integrative and complementary practice that is part of anthroposophic medicine recognized by the National Policy on Integrative and Complementary Practices in Brazilian Health.

**Methods**

Literature review on Medical Painting Therapy in Liane Collot d'Herbois methodology as a practice of Anthroposophic Medicine.

**Results**

This methodology comprises a therapeutic process where the patient - through art - expresses a temporal condition of your consciousness, feelings and body. From the diagnosis and performing art, with an orientation based on the fundamental principles of the methodology that the principles work of "Light, Color and Darkness", the patient begins to dissolve their standards, composing an ideal paint, a picture of a healthy composition related to him. It is understood that the human being is a microcosm of the macrocosm, a reflection of the outside world. From this concept, the principle of light is related to the upper pole, the neurosensory system; the principle of darkness is the lower pole, the metabolic system; the meeting of the two forces takes place in the middle area, it happens on the rhythmic system, which hosts the circulation and breathing processes. The treatment happens in the meeting between light and darkness, when the colors are brought in their own movements, environments, reaching space, artistic and vital elements (moisture, warm, surfaces and substance, freshness) and creating an image of nature. In humans, treatment refers to the achievement of a rhythm; creating space in the rhythmic system so that breathing and circulation happen harmonically, and others vital processes are stimulated and enhanced. This process allows the patient to achieve greater self-awareness, re-establish better social relationships, rekindle their life processes. This methodology has been recognized worldwide, from doctors and patient reports whom recognized that reached a better quality of life in their thinking aspects, feelings and their work. Evidence suggests that this treatment can lead to decreased pain, improved vitality, restoring senses, in addition to improvement in mood and wellbeing. There are reports of improvement of physical symptoms and decreased use of medications.

**Conclusions**

The Medical Painting Therapy in Liane Collot d'Herbois methodology demonstrates how therapeutic intervention with low cost, enjoyable and non-invasive aspect, can help. It brings together great potential as a resource of integrative medicine for achieving better quality of life in its integrality, working in the biological, psychological and social dimensions.

## VARIOUS TOPICS

### P327 Medicinal plants with cardiac side effect from the perspective of Traditional Persian Medicine

#### Jafar Abolghasemi, Mojtaba Heydari

##### Research Centre for Traditional Medicine and History of Medicine, Shiraz University of Medical Sciences, Shiraz, 71348457, Iran, Islamic Republic of

###### **Correspondence:** Jafar Abolghasemi (jafar20200@yahoo.com)

**Introduction**

Medicinal plants are used popularly in traditional systems of medicine. In Traditional Persian Medicine (TPM) pharmacopeia, beside therapeutic effects of herbs, their adverse effects on different body organs and the way of minimizing these side effects, are discussed from a traditional perspective. The aim of this study was to review herbal drugs with cardiac side effect from the viewpoint of TPM.

**Methods**

The most comprehensive TPM pharmacopeia "Makhzan-al-Advyeh" were searched for herbal drugs with cardiac adverse effects. The characteristics of these herbs were reviewed and compared to find a common mechanism to rationalize their adverse effect on the cardiac system. The way for minimizing these side effects was also classified.

**Results**

From 700 medicinal plants mentioned in Makhzan-al-Aladvyeh, 6 herbs are considered to have an adverse effect on the heart. Most of these herbs (85%) were classified as hot and dry temperament. Adding Gum Arabic and lemon juice to the formulations with these herbs was the most popular way of decreasing the side effects. Six popular herbs with cardiac side effect from TPM perspective and their characteristics are summarized in the Table 3 below.

**Conclusion**

The survey introduced medicinal plants with potential cardiac side effects in TPM which can be more investigated in modern studies.

Keywords: Adverse effect, cardiac side effect, heart, Traditional Persian Medicine

**Table 3 (Abstract P326).** See text for description

Traditional name	Scientific name	English name	Temperament	Detoxify
Ban	Coffea arabica	Coffee shrub of Arabia	Cold and dry	Crocus sativus
Tanbakoo	Nicotiana rustica	Mapacho	Hot and dry	
Orogh alsafsr	Curcuma longa	Turmeric	Hot and dry	Lemon juice
Osghologhndrion	Asplenium scolopendrium	Hart's-tongue	Hot and dry	Gum Arabic
Asaabe alsafar	Vitexagnus-castus	Vitex	Hot and dry	Gum Arabic
Kholenjan	Alpinia officinarum	Lesser galangal	Hot and dry	Trachyspermum ammi

### P328 The application of the State-Regions National Agreement for CM education in Italy: The state of the art

#### Sonia Baccetti^1^, Elio Rossi^2^, Paolo Fedi^2^, Mariella Di Stefano^2^, Katia Belvedere^3^

##### ^1^ Centre of Acupuncture and Traditional Chinese Medicine, Tuscan network for Integrative Medicine, Florence, 50133, Italy; ^*2*^*Tuscan network for Integrative Medicine, Florence, 50139, Italy;*^*3*^*Health Directorate, Region of Tuscany, Florence, 50139, Italy*

###### **Correspondence:** Sonia Baccetti (sonia.baccetti@uslcentro.toscana.it)

**Background**

In 2007, the Tuscan Regional Council approved the Regional Law of Tuscany n. 9/2007, which regulates education and practice of Complementary Medicine (CM): acupuncture, herbal medicine, homeopathy, homotoxicology and anthroposophy, by medical doctors, dentists, pharmacists and veterinarians. On this basis a State-Regions Agreement on national rules of CM education was finally approved in February 2013. It defines criteria for education and accreditation of professionals and schools, and provides lists of professionals who practise CM.

**Purpose**

Verify the situation of the Italian Regions as regards the application of State-Regions Agreement on national rules of education in Complementary Medicine in Italy approved on February 2013.

**Methods**

All the Departments of Health of the Italian regions and autonomous provinces have been consulted, as well as their respective websites in order to verify the phase of regional application of the agreement, and their deliberative acts have been examined and downloaded.

**Results**

Till now 11 regions out of 20 have deliberated in application of the Agreement in different ways. 3 regions have approved specific regional laws: Marche in 2013, Umbria in 2014, Piedmont in 2015, and Tuscany Region approved the necessary changes to its Regional law (n. 9/2007) in 2016. Specific deliberations were instead approved by Puglia in 2013, Sicily 2014, Emilia Romagna 2014-15, Sardinia 2015, the autonomous Province of Trento 2015 and Bolzano 2015 Lombardia, Valle d'Aosta 2014-2016.

**Conclusions**

The phase of application of the National Agreement is still ongoing and the commitment of CM professionals and patients will facilitate its complete application.

### P329 Recent advances in the integration of CM in public healthcare system of the region of Tuscany

#### Sonia Baccetti^1^, Elio Rossi^2^, Fabio Firenzuoli^2^, Mariella Di Stefano^2^, Katia Belvedere^3^

##### ^1^ Centre of Acupuncture and Traditional Chinese Medicine, Tuscan network for Integrative Medicine, Florence, 50133, Italy; ^*2*^*Tuscan Network of Integrative Medicine, Florence, 50139, Italy;*^*3*^*Health Directorate, Region of Tuscany, Florence, 50139, Italy*

###### **Correspondence:** Sonia Baccetti (sonia.baccetti@uslcentro.toscana.it)

**Purpose**

To describe new advances of the process of integration of complementary medicine (CM) in the Public Healthcare System of the Region of Tuscany in tune with the reorganization of the Regional Health System in place from 2015.

**Methods**

The integrated complementary medicine activities will be developed according to the following criteria: 1) optimization of the CM offer ensuring equitable geographical distribution and coverage of the priority areas of intervention among the various MC activities operating within the same Local Health Unit (USL); 2) development of the interaction between hospitals and universities within the same USL and at the regional level; 3) improvement and reorganization, within the healthcare pathways of the network nodes of CM, to ensure the realization of the essential regional priority goals;

**Results**

The priority and common objectives of integration of all the CM services of the region are: fight against pain; integrative oncology; promotion of physiological birth in low-risk pregnancies; gender medicine (menopause and gender oriented metabolic disorders); prevention and treatment of respiratory diseases and childhood and adulthood atopic diseases. Moreover specific projects will be developed in relation to an integrated management of chronic diseases and patients with multiple chemical sensitivity or drug hypersensitivity. The Local Health Units will also have to find their own Coordination Centre of CM, with the function to guarantee the best offer of MC services on the territory.

**Conclusions**

The Tuscan example show that evidence-based complementary treatments CM can be effectively integrated within the Public Healthcare System.

### P330 "It brings her back to herself"- the efficacy of eurythmy therapy in stroke rehabilitation

#### Katherine Beaven, Anita Rose, Gerhard Florschutz

##### Eurythm Therapy, Raphael Medical Centre, Hildenborough, United Kingdom

###### **Correspondence:** Katherine Beaven (katherine@bevbach.co.uk)

**Purpose**

Stroke is the leading cause of serious, long-term disability among adults. In the United Kingdom, each year between 174 and 216 people per 100,000 people are affected by stroke. The main effects of a stroke include cognitive deficits, compromised sensory function and motor impairments and psychological sequalae. The current approach to stroke rehabilitation in the UK involves a multidisciplinary team approach focusing on minimizing disability and handicap, encouraging a return to independence and activities of daily living and increasing life satisfaction. Rarely are complementary therapies involved. This study considers the ways in which Eurythmy therapy can contribute to a stroke patient’s rehabilitation in the context of an inpatient neurorehabilitation hospital. Such a study is the first of its kind.

**Methods**

A case study research approach was adopted and the findings are based on three stroke patients. The research included both qualitative and quantitative data.

**Results**

The findings from this research provide evidence that Eurythmy therapy can address and bring improvement to a broad range of problems including increased strength, flexibility, co-ordination, control, body awareness, uprightness and balance. In addition, it can support improvements in the patient’s psychological wellbeing.

**Conclusions**

The conclusion of the study noted that Eurythmy therapy has a unique potential to meet a patient’s physical, emotional and spiritual needs as a whole and can therefore, make an important contribution to a patient’s rehabilitation.

### P331 Developing and evaluating a health related quality of life (HRQOL) questionnaire for craniosacral therapy (CST): Using qualitative methods to evaluate a conceptual framework

#### Nicola Brough Phil, Helen Parsons, Sarah Stewart-Brown

##### Warwick Medical School, University of Warwick, Coventry, CV4 7AL, United Kingdom

###### **Correspondence:** Nicola Brough Phil (n.brough@warwick.ac.uk)

**Background**

Craniosacral Therapy (CST) is a body-based complementary or alternative medical practice which aims to support natural healing mechanisms. It is increasing in popularity and clients anecdotally report health improvements. The practice lacks an evidence base partly due to the absence of suitable Patient Reported Outcome (PRO) measures. PRO development is in its infancy in CST and in CAM more generally, despite being an integral part of patient-care, policy decision making and health care delivery.

**Purpose**

To evaluate a conceptual framework (CF) of CST outcomes as part of a study to develop and validate a new PRO questionnaire for CST.

**Methods**

The CF was evaluated in focus groups. 7 CST practitioners in 2 focus groups discussed the content, layout and design of the CF. Three CST clients evaluated the refined CF.

**Results**

*Practitioner Summary:* Participants suggested the CF had value in helping users understand the scope of CST, recommended that the social wellbeing domain be expanded. The inclusion of spirituality was debated.

*CST Clients Summary:* Discussions centred on self-care and taking responsibility for ones health. Spirituality was seen as important and debate focused on how to position its role in healthcare and the appropriate terminology to use. Participants liked the complexity of the CF.

**Conclusions**

It is important to have input from both patients and practitioners when designing a PRO, to ensure outcomes of relevance are not missed. Focus group methods lent themselves to this part of the questionnaire development.

Ethics granted - University Biomedical Research Ethics Sub-Committee REGO-2015-1499.

### P332 A policy-based, native Hawaiian medicine-driven curriculum adaptation for health equity and social justice in Hawai’i

#### Katherine Burke (kymburke@hawaii.edu)

##### Office of Public Health Studies, University of Hawaii, Honolulu, HI, United States

**Question**

Can integrative medicine help to eliminate health disparities in Hawai’i? Currently there is a void of education and training for future health professionals who seek to promote health equity in Hawai’i. New legislation Act 155 requires that all future health programming prioritize health equity by addressing the social determinants of health, in particular, social justice for Native Hawaiians. To enforce this legislation, Senate Resolution 60 created a Task Force focused specifically on Native Hawaiian Health. In response to these two pieces of legislation, Nā Limahana o Lonopūhā Native Hawaiian Health Consortium (NLOL), adapted Stanford University’s Public Health Advocacy Curriculum to educate future health professionals about the social and cultural determinants of health in Hawai’i at the pre-health, undergraduate level. Using Nā Pou Kihi, Determinants of Kānaka ‘Ōiwi Health, a logic model developed by NLOL member and Task Force leader, Department of Native Hawaiian Health at the John A. Burns School of Medicine, the curriculum provides students with tools to apply principles of Native Hawaiian medicine to modern medical treatment interventions. Guided by this model and an archive of Hawaiian medical curricula, the adaptation relied on input from community scholars and cultural practitioners to ensure content was adapted with integrity and rigor.

**Methods**

Curriculum drafts were presented to community members with a career-long commitment to addressing the social determinants of health in Hawai’i. Focus groups were held with environmental policy advocates at the Office of Hawaiian Affairs and NLOL-identified content experts were invited to participate in interviews. Participants were asked to provide candid feedback based on their professional experience on draft content. All focus groups and interviews were audio-recorded and scribed. Data from the focus groups and interviews was used to edit the first round of drafts which were then revised and returned to the experts for further review. (University of Hawai'i Human Subjects Protection review approved CHS# 30433)

**Results**

Two, 2-hour focus groups with 9 environmental policy advocates and 6 2-hour interviews with content experts were conducted. Participants shared feedback that was formative in adapting the curriculum. The process ensured that existing curricula were integrated with integrity and benefited from the practical knowledge of the participants in selecting the most effective elements for inclusion.

**Conclusions**

The iterative, interconnected methodology of this curriculum adaptation process is an effective way to maximize community scholarship to promote health equity and social justice in Hawai’i.

### P333 Development of a consortium on Integrative Medicine & Health in the Netherlands: Barriers and facilitators

#### Martine Busch^1^, Fenna Heyning^2^, Jan Smit^3^, Hans Jeekel^4^, Hans de Goeij^5^

##### ^1^ Van Praag Instituut, Utrecht, 3511 VH, Netherlands; ^*2*^*STZ Association of Teaching Hospitals, Utrecht, Netherlands;*^*3*^*Internal Medicine, Radboud University Medical School, Nijmegen, Netherlands;*^*4*^*Surgery, Erasmus University Medical School, Rotterdam, Netherlands;*^*5*^*Ministery of Health, Den Haag, Netherlands*

###### **Correspondence:** Martine Busch (mbusch@vanpraaginstituut.nl)

**Background**

The use of Complementary & Integrative Medicine (CIM) is widespread in the Netherlands. A national survey demonstrated that almost all (92%) Dutch hospitals offer some CIM, mostly without adequate policy and procedures. However, without any local or national research agenda, there is no collective scientific effort to bring CIM to a more evidence based level of patient care and safe usage.

**Objective**

To investigate barriers and facilitators for the development of a Dutch Consortium of Integrative Medicine & Health in order to guide policy and research on CIM.

**Method**

The Dutch Organisation for Health Research and Development (ZonMw) installed a steering committee to guide the process of developing a consortium on integrative medicine and health. Stakeholders from mainstream healthcare and CIM organisations were interviewed on perceptions, needs and concerns about CIM research. Relevant topics were discussed at an invitational conference. American IMH centres were visited and international experts interviewed on advocacy and setting a research agenda.

**Results**

There is an overall need for more research on CIM in the Netherlands, specifically in terms of safety, efficacy and patient outcome. Barriers found were: institutional commitment, funding, terminology and framing. Facilitators were connecting it with new health concepts like Positive Health, and openness and support from medical doctors and hospital administrations.

**Conclusion**

A pragmatic strategy will be chosen, taking into account barriers and facilitators, to establish a consortium with 5-7 pioneering health centres in the Netherlands to promote structured integration of CIM with focus on step-by-step knowledge and research building.

### P334 Integrative Medicine Group: A 10-year experience of an official team at a pediatric tertiary hospital

#### Paulo Caceres Guido^1,2^, Norma Barraza^1^, Ziomara Balbarrey^1^, Alejandra Ribas^1^, Beatriz Jimenez^1^, Claudia Iachino^1^, Fabiana Quattrone^1^, Marisa Gaioli^1^, Marta Dell´Orso^1^, Silvia Villanueva^1^, Carmen Rocha^1^, Adriana Macchi^1^

##### ^1^ Hospital de Pediatria Garrahan, Universidad Maimonides, Buenos Aires, 1245, Argentina; ^*2*^*Universidad Maimonides, Buenos Aires, Argentina*

###### **Correspondence:** Paulo Caceres Guido (caceresguido@gmail.com)

**Purpose**

To describe the experience of an Integrative Medicine Group (IMG), the first official multidisciplinary team at a pediatric tertiary referral hospital in Argentina, during its first 10 years.

**Methods**

We quantitatively and qualitatively evaluated, through IMG records, provision of clinical services, teaching, and research (2006 – 2016).

**Results**

More than 5000 persons received IMG care (patients/parents: 65% – hospital staff: 35%). More than 1400 sessions were offered; the following are worth mentioning: reiki, singing bowls, guided imagery, mandalas, infant massage, horticultural therapy, mindfulness, and reflexology. Health conditions frequently consulted were, for patients: pain, fear, anxiety, insomnia, dyspnea, nausea/vomiting, and fatigue; and for staff: fatigue, hypertension, anxiety, headache, muscle contracture, gastritis, and stress. Overall, 58 lectures/workshops were given for intra- and extra-hospital staff, on a variety of integrative topics. Twelve scientific studies, including lectures, book chapters, and publications in national and international scientific journals, some in partnership with universities and research institutes, were presented. The IMG was allowed to provide consultations at other departments and on the wards, and IMG facilities were created inside the Hospital. In 2016 the IMG received a national Award for Innovation (Applied Research project) from the Ministry of Science and Technology, Argentina.

**Conclusions**

The IMG of Garrahan Hospital is the first official reported group working in pediatric integrative medicine at a public hospital in Latin America. These first ten years have shown that it is possible to work with integrative medicine at a pediatric tertiary referral hospital with satisfactory results in the clinical setting, teaching and research.

### P335 The application of scientific & technology achievements in the area of CAM (Complementary and Alternative Medicine) in China: Some considerations and suggestions from the policy aspect

#### Jianxiong Cai^1^, Lina Chen^2^, Darong Wu^1^, Sicheng Wang^2^

##### ^1^ The TCM Clinical Outcome Assessment Team, The Second Affiliated Hospital of Guangzhou University of Chinese Medicine, Guangzhou, 510120, China; ^*2*^*Science and Technology Division, State Administration of Traditional Chinese Medicine of the People of China, Beijing, 100027, China*

###### **Correspondence:** Sicheng Wang (wangsicheng@263.net)

**Purpose**

The purpose of this study is to support the strategy of the transformation process in Complementary and Alternative Medicine (CAM) in regards of the scientific and technology achievements.

**Method**

Base on the characters and tendency of CAM, we reviewed the successful model and mechanism of technology transfer in the developed countries and districts, and provided some promoting strategies which might be applied in China.

**Result**

We suggested some advices to promote the technology transfer of CAM, including (1) formulate sound legal system for technology transfer of CAM, strengthen the guiding and service function of government, and clearly define the right and obligation of stakeholders; (2) Increase the science and technology investment and keep rising stably and continually; (3) Develop more incentive mechanism and talent cultivation policy of technology transfer, improve the operability of property rights of technology; (4) Establish professional and diversity science-technology agency service system, integrating more transfer channels to digest the technology stock; (5) Carry out the pertinence and targeted work basis on the precise classification of CAM researches and national circumstance; (6) Encourage the scientific researchers turn to follow the guide of practical technology and products, and face the demands of markets and people; (7) Improve the monitoring and evaluation methods of technology transfer, and enhance the right of public access to the research information.

**Conclusion**

Promoting the technology transfer of CAM will plays a positive role to the development of CAM.

Keywords: Complementary and Alternative Medicine, scientific & technology achievement transformation, strategy

### P336 Integrative therapy for improving breast size and elasticity: Three case reports

#### Eunji Choi, Namgyeong Go, Yongho Lee

##### ONBODYCLINIC, Seoul, South Korea

###### **Correspondence:** Namgyeong Go (simbi01@naver.com)

**Objective**

Breast augmentation surgery and autologous fat grafting are frequently performed to improve the quality of life and self-esteem of patients. However, there is a risk of complications with these procedures; therefore, alternative treatments are needed. The aim of this study was to introduce integrative therapy involving traditional Chinese medicine (TCM) and conventional medical material (polydioxanone threads) embedding for improving breast size and elasticity.

**Methods**

Three patients were treated with TCM, such as acupuncture and herbal medicine, and polydioxanone (PDO) thread embedding therapy. Treatment efficacy was assessed based on the breast cup size, which was defined as the difference between the circumferences of the bust and under bust, and a self-evaluated questionnaire score on enhancement of breast size and elasticity. The side effects of treatment were also monitored.

**Results**

The breast cup sizes of three patients increased after treatment and considerable improvements in patient satisfaction were observed with respect to breast size and elasticity. There were no severe adverse events.

**Conclusions**

The results suggest that integrative therapy using TCM and PDO thread embedding could be a complementary and alternative option for enhancing breast size, elasticity, andsatisfactory on self-evaluation. Long-term follow-up and further studies are needed.

**Consent**

Written informed consent for the publication of this case study was provided by each patient,and the principles of the 1964 Declaration of Helsinki were followed in this study.

### P337 Health promotion through traditional medicine: A community based approach

#### Gokarna Dahal (dahalgokarna7@gmail.com)

##### Department of Ayurveda, Ministry of Health, Kathmandu, Nepal

In recent year, the integration of traditional medicine in to main stream medical practices and public health program has gained momentum.However, there are major challenges and discrepancies in the pace of adoption and implementation of traditional medicine in the name of evidence based practices. The main reason does not only lie in the scientific explanation but also the in efficient community mobilization and poor understanding of potential of that traditional medicine offer to combat emerging non communicable diseases. Hence, we present and critically discussed the example of the success implementation of traditional medicine to combat non communicable disease in low resources setting country like Nepal.

We focus the fully community participation, mobilization of traditional healers along with Ayurveda female community health volunteers through establishment of weekly outreach clinic at village level. We tried to use traditional approach for health promotion activities and improvement of quality of life. The management and operation of that outreach clinic fully governed by the outreach clinic management committee that is formed in inclusively at local level. The Technical support provided by the concern authority in that district. This activities not only focus the utilization, enrichment of traditional knowledge but also the conservation of traditional knowledge and ethno medical prospects. People are not only treated but treated with happiness and full deserve of their belief.

We belief that as healthy community initiative mature,many of them are offer their work in building community consensus for improved health care,often quality of life issue can be transferred in to public policy.

### P338 Implementation of an academic center of Integrative Medicine and Health (CIMH) under German Federal Republic legislation – organizational and economic challenges

#### Xaver Frauenknecht, Heike Gerhardt

##### Sozialstiftung Bamberg, Bamberg, 96049, Germany

###### **Correspondence:** Xaver Frauenknecht (vorstand@sozialstiftung-bamberg.de)

**Background**

Whereas 40-60% of the German population wish to use complementary or alternative therapies, the German health system and health legislation are reluctant to accept such approaches. Integrative medicine (IM) with its patient-centered and evidence-based approach has been shown to have the potential to overcome such reservations.

**Objective**

To describe the organizational and economic aspects of the implementation of an academic CIMH at a German teaching hospital in Bamberg, Bavaria.

**Methods**

A national and regional needs analysis for inpatients and outpatients was performed. In- and outpatient spaces, academic leadership, specialized health professionals and reimbursement strategies were analyzed and implemented..

**Results**

Major efforts were necessary to explain the concept and practice of IM to all stakeholders. Oncology, pediatrics, gynecology, psychosocial disciplines and chronic pain programs were most responsive. A lack of specialized health professionals had to be overcome by appropriate training programs. The new building of a specialized ward, day and outpatient clinic was imperative for comprehensive acute and long term IM patient care and effective research and educational programs. The reimbursment policies indicate sustainable self-support of the center´s patient care within 3-5 years. The academic part, realized with the Faculty of Medicine of the University of Erlangen-Nueremberg, included an endowed professorship.

**Conclusion**

A newly established academic CIMH faced substantial organizational and economical challenges which call for urgent changes of health policies in Germany.

### P339 Advances in Complementary Medicine regulation in Chile

#### Mónica Galanti^1,2^, Carmen J Cerda^1^

##### ^1^ Public Policies, Ministry of Health, Santiago, 8320000, Chile; ^*2*^*Pediatric, University of Chile, Santiago, Chile*

###### **Correspondence:** Mónica Galanti (monicagalanti@gmail.com)

The Chilean Ministry of Health creates the Unit for Complementary Medicine in 2002. The first objective was to gather information about herbal medicine in Chile. Between 2005 and 2008 the practice of Acupuncture, Naturopathy and Homeopathy is regulated. Between 2010 and 2012 studies lifting information about complementary medicine in health institutions showed that 35% of Primary Care Centers had some type of complementary medicine, of which 30% were exercised by doctors, but the great majority were volunteers not health professionals. The practice of complementary medicine was highly appreciated by the users of the health system and health professionals. The most important difficulty was the lack of physical space and financial resources. Health problems more frequently attended were: chronic pain, headache, depression and back pain. Therapies offered most frequently were: floral therapy, biomagnetism, reiki, yoga and sintergética medicine and population of higher socioeconomic strata had greater access to such therapies. Considering the high demand of population for complementary medicines and recognizing the great inequity in access to them, the Chilean Ministry of Health accelerates the development of a policy on complementary medicine, recognizing the contribution they can offer in relation to self-care, healthy lifestyle and prevention of chronic diseases. Meetings with different therapies were held to agree definitions, historical and scientific background, performance areas and therapist profile. With this information, the Ministry draws up a Manual of Complementary Practices addressed to Primary Health Care users, along with a virtual information page where they can find more information about each therapy.

### P340 Chilean complementary medicine record

#### Mónica Galanti^1,2^ (monicagalanti@gmail.com)

##### ^1^ Ministry of Health, Public Policies, Santiago, Chile; ^*2*^*University of Chile, Pediatric, Santiago, Chile*

About a third of hospitals and primary health centers in our country offer complementary therapies without being regulated and without recording them as such. Considering this situation, Chilean Ministry of Health starts in 2016 a pilot project in order to validate a national registration system for complementary medicines and thus know the national reality in relation to them in our Health Service System. We contacted nine centers of primary, secondary and tertiary health establishments which already had Units of Integrative Medicine; they were instructed in the use of the proposed registry and the implementation of a questionnaire to assess user satisfaction before and after therapy. Actually we are about to finish this survey by December this year so we hope to work with this registry from 2017 onwards.

### P341 Chilean health workers welfare

#### Mónica Galanti^1^, Daniela Navarrete Heckersdorf^2^, Héctor Jorquera^3^, María L Alcázar Saldivia^4^

##### ^1^ Public Policies, Ministry of Health, Santiago, Chile; ^*2*^*Department of Quality of Life and Labor Relations, Chilean Health Ministry, Santiago, Chile;*^*3*^*Chilean Health Service, Santiago, Chile;*^*4*^*Department of health explicit guarantees and High Complexity Networks, Chilean Health Ministry, Santiago, Chile*

###### **Correspondence:** Mónica Galanti (monicagalanti@gmail.com)

After an assessment on quality of life of Chilean health workers during 2016, high rates of mental health problems were observed. Depression is the leading cause of work absenteeism, equivalent to 626,177 days (64%), alcoholism was associated with 1,639 days of absenteeism and mental disorders by the use of drugs (opioids, cannabinoids, sedatives or hypnotics, cocaine, stimulants, cigarettes, volatile solvents and other psychotropic substances) were responsible for 2,435 days of absenteeism from work, cocaine being the substance with greater impact. It was not possible to obtain specific information regarding suicide rate among health service workers, however, as the General Standard Administrative of our Ministry of Health states, mental illness is one of the most important factors predisposing suicidal behavior. People affected by mental illness are at risk of suicide ten times higher than those who do not suffer this health problem. Mental health problems more often associated with suicidal behavior are depression and bipolar disorders, drug and alcohol abuse and schizophrenia, so we might assume that suicide rate in our health care system is not negligible.With the aim to resolve this dramatic reality we contacted the Worker’s Health Unit (UST) from the southern area of Santiago, who for several years have been carrying out a health care unit that includes complementary medicine for their workers which has demonstrated a high degree of satisfaction among them. Given this successful results we seek to repeat and evaluate this experience in other health centers in the country.

### P342 Perception of Integrative Medicine by patients with cancer history: Qualitative research - content analysis

#### Daiva Jakubonienė (daivaj@doctor.com)

##### Natūraliosios terapijos centras, Vilnius, 08119, Lithuania

**Question**

Cancer patients are a special category of patients because of life risk, complexity of treatment and psychological burden of desease. The aim of the study is to evaluate their perception of Integrative medicine and to define their main needs in their relationship with health professionals.

**Method**

Interviews with 6 patients having different duration of cancer history were made in June-September 2016. Questions about advantages and disadvantages of academic and alternative medicine, their relation to both and modeling of future integrative medicine were given. Content analysis of answers was performed.

**Results**

Main noticed disadvantages of academic medicine were lack of interest in a patient, being profit oriented, non willing to know alternative approach. Alternative medicine was seen as not always equally effective, hostile to academic treatments. The need of the patient was to use both medicines for the best health result. The lack of knowledge in alternative medicine of academic health professionals was seen as the main source of confrontation between the two medical directions. Ideal integrative medicine was imagined as patient oriented and free of relation to profit, ecological and environment friendly.

**Conclusion**

Obligatory informing of medical students about basic concepts of alternative medicine would be favorable to development of integrative medicine.

### P343 Cardiometabolic disease, chocolate and red wine: Raising the bar and a glass to an old foe

#### Bradley McEwen (brad.mcewen@endeavour.edu.au)

##### Department of Nutritional Medicine, Endeavour College of Natural Health, Sydney, 2000, Australia

**Question**

Cardiometabolic disease is a multifactorial disease with numerous risk factors, including platelet hyper-aggregation, increased coagulation, diabetes, dyslipidaemia, inflammation, overweight/obesity, physical inactivity, and poor nutrition. Diet and nutrition are significant modifiable risk factors in the prevention and risk reduction of cardiometabolic disease. Diets higher in fruit, vegetables, and fish have been associated with reduced cardiometabolic risk. Whereas diets high in red and processed meats, potatoes, refined grains and sweets were associated with a higher risk of cardiometabolic disease. A review of the literature was conducted to investigate the health benefits of chocolate and red wine and whether they modify cardiometabolic risk.

**Methods**

PubMed, EBSCO, and Sciencedirect databases were searched using combinations and variations of the following search terms: cardiometabolic disease, cardiovascular disease, diabetes, cholesterol, platelets, chocolate, and wine.

**Results**

Chocolate consumption was associated with reduction in cardiometabolic risk factors such as blood pressure, cholesterol, platelet function, atherosclerosis, and insulin resistance. The mechanisms included antioxidant, anti-inflammatory, anti-atherogenic, reduced low-density lipoprotein (LDL) oxidation, antiplatelet, anti-thrombotic, peripheral vasodilation, and antihypertensive effects as well as influencing insulin sensitivity and vascular endothelial function. The consumption of wine was associated with the reduction of several cardiometabolic risk factors, such as blood pressure, LDL and high-density lipoprotein (HDL) levels, inflammation, serum glucose, as well as improvement in endothelial function. The mechanisms included antioxidant, anti-inflammatory, inhibition of inflammatory cytokines, reduction of reactive oxygen species (ROS), signal transduction, and inhibition of platelet aggregation.

**Conclusions**

Chocolate and red wine have numerous cardiometabolic health benefits, including antioxidant, anti-inflammatory, anti-atherogenic, antihypertensive, and cholesterol modifying properties.

### P344 The use of medicinal plants in Cerrado region (Federal District, Brazil)

#### Francislete Melo^1^, Fernanda Mulinari Fontana^1^, Ana C Viana Valle^2^, Maria T Borges Neres^1^

##### ^1^ Pharmacy, UPIS, Brasília, 70390-12, Brazil; ^*2*^*Natural Pet, Brasília, Brazil*

###### **Correspondence:** Francislete Melo (etemelo@gmail.com)

Brazil is a country of interest to ethnopharmacology. The six ecological domains, namely, Amazonian forest, Caatinga, Pampas, Cerrado, Atlantic Forest and the Pantanal are source of cultural and biological diversity. The use of medicinal plants in nature or in the form of herbal medicines is an important source to obtain phytotherapeutic agents. Thus, this work consists of an ethnopharmacological survey of medicinal plants used by people from Federal District, carried through research of field in thirty five pharmacies and nine free and permanent fairs. The pharmacy most widely used herbal medicine for nervous system is composed of *Passiflora incarnata*, for the digestive system is the *Boldus pneumus* and for respiratory system highlights the *Mikania glomerata*. Interestingly the survey of the medicinal plants requested from and/or indicated by herb sellers in fairs are barbatimão (*Stryphnodendron barbatiman)*, Chapéu-de-couro (*Echinodorus macrophyllus*), cavalinha (*Equisentum giganteum*), aroeira (*Schinus terebinthifolious*, jatobá (*Hymenaea coubaril*) and copaíba (*Copaifera reticulata dunke*). Medicinal plants more commercialized in DF fairs are not found in the best-selling herbal products, demonstrating that there is an important difference between the popular use and availability of commercial products. The plants requested from and/or indicated by herb sellers are commonly used in the region, with few studies by the ethnobotanical, safety of plants and herbal medicines production viability.

### P345 Traditional Persian Medicine: A source of ideas for sexual dysfunction in cardiovascular patients’ investigations

#### Abolali Mohagheghzadeh^1,2^, Mohammad E Zohalinezhad^1^

##### ^1^ Museum, Archives and Cultural Studies of Norani Vesal, Shiraz University of Medical Sciences, Shiraz, Iran, Islamic Republic of; ^2^ Pharmacognosy, Shiraz University of Medical Sciences, Shiraz, Iran, Islamic Republic of

###### **Correspondence:** Mohammad E Zohalinezhad (zohalinm@sums.ac.ir)

**Introduction**

Avicenna (980-1037 AD) as a pioneer of Persian physician has described numerous diseases in various fields in his famous book, "The Canon of Medicine" [1]. The book contains principles of different diseases and respective treatment approaches [1]. It also contains a full chapter to the description of libido, its disorders and treatments [2, 3]. In the subchapter of impotence, Avicenna has discussed on the etiology and treatment of sexual dysfunction [2, 3].

**Materials and Methods**

"Sexual dysfunction", "Traditional Persian Medicine (*TPM)," Tribulus terrestris*" and "cardiovascular disorders" was searched in "*Scopus*", "*web of science*" and "*pubmed*" and then, electronic copies of "the canon of medicine" and "Makhzan al-Advia" was reviewed for its advises to cardiovascular patients with sexual dysfunction.

**Results**

According to Avicenna’s concepts, impairment of vital organs such as heart and their association with Sexual dysfunction, should be considered [2]. However, there is a few Food and Drug Administration (FDA) approved drugs for treating Sexual dysfunction of cardiac patients, we can find many drugs for treatment of them in TPM texts books such as *Tribulus terrestris* [4, 5]. In the Canon of Medicine and Makhzan al-advia, safety and effectively influences of *Tribulus terrestris* on cardio vascular system and libido, were mentioned [3, 6].

**Conclusions**

*Tribulus terrestris* as an herbal remedy has shown beneficial aphrodisiac effects in a number of animal and human experiments and can improve desire and sexual dysfunction in cardiovascular patients with hypoactive sexual desire disorder [4, 6]. Furthermore investigations may be needed to approve *Tribulus terrestris* as a drug of choice of sexual dysfunction in cardiac patients.

Keywords: *Tribulus terrestris*, Sexual dysfunction, cardiovascular disorders, Traditional Persian medicine

**References**

1. Zargaran A, Mehdizadeh A, Zarshenas MM, Mohagheghzadeh A. Avicenna (980–1037 AD). Journal of neurology. 2012;259(2):389-90.

2. Zohalinezhad ME, Zarshenas MM. Cardiovascular aspects of erectile dysfunction as outlined by Avicenna. Int J Cardiol. 2014;175(2):e33-e4.

3. Sina I. The canon of medicine: Soroosh Press; 1998.

4. Akhtari E, Raisi F, Keshavarz M, Hosseini H, Sohrabvand F, Bioos S, et al. Tribulus terrestris for treatment of sexual dysfunction in women: randomized double-blind placebo-controlled study. DARU Journal of Pharmaceutical Sciences. 2014;22(1):1.

5. Sanders M. Sex, Drugs, and Advisory Committees: An Analysis of Pharmaceutical Industry Manipulation of FDA Vulnerability to Sociopolitical Influences on Matters of Women's Health. Columbia Human Rights Law Review. 2016;48(2016-2017).

6. Aghili M. Makhzan-al-Advia. Tehran: Tehran University of Medical Sciences. 2009;328.

### P346 The organization of BDORT treatment center

#### Olja Njaradi, Momir Dunjic

##### BDORT Center, Belgrade, 11000, Serbia

###### **Correspondence:** Momir Dunjic (dr.momirdunjic@gmail.com)

Serbia, as a country in transition, is facing numerous challenges in its National Health System. This also affects those national health institutions that specialize in diagnosing and treating autism spectrum disorder (ASD). Number of these institutions and their organizational infrastructure do not satisfy the needs of potential users especially ones interested in integrative medical approach in treating these kinds of disorders. The users are forced to search individually for these kinds of institutions and often specialists of different profiles that work with persons diagnosed within ASD have different methodologies and offer differing treatments. The purpose of this paper is to present the unique organization of BDORT Center – as the first center in Serbia and in the region which joins integrative medical approach together with behavioural therapy. This way of work allows a prompt diagnosis using noninvasive BDORT method, identification of adequate supplementation with the program of behavioural therapy unique to every user, and a systematic monitoring by the team of specialists (doctors, SLP and psychologist). This integrative approach supports early intervention and induction of necessary changes during the process of treatment which are of crucial importance for its success. Further, the BDORT Center is organising workshops for parents with ASD children to support them through education about the relevant themes related to the nature of ASD as well as through mutual exchange of experiences. The goal is to develop recognizable, integrated and coordinated system of early intervention which will be sustainable in spite of inconsistency in current practices.

### P347 The role of speech and language pathologist in the treatment of autism

#### Olja Njaradi, Momir Dunjic

##### BDORT Center, Belgrade, 11000, Serbia

###### **Correspondence:** Momir Dunjic (dr.momirdunjic@gmail.com)

The purpose of this paper is to present the role of speech – language pathologist in the treatment of children diagnosed within ASD implemented in BDORT Center. Since children within this spectrum are very different regarding their communicative, cognitive, motoric and adaptive skills, the focus is on making individual plan of treatment. The program of treatment and the development of the child is evaluated periodically, by psychological testing, so the correction and needed adjustments to the program could be conducted. The method of work is heavily related to the core principles of behavioural therapy, which we adapt to the local conditions of users. After the initial evaluation in BDORT Center, we suggest intensive treatment, between 15 and 18 hours per week. During the process of treatment great attention is dedicated to the education of parents about the way their children learn and about their role in helping their children learn. This is because we consider parents as co-therapists whose role in helping their children to generalize and apply learned kills in everyday life is of crucial importance. This is also due to the fact that many children upon entering primary school are not able to continue with an intensive treatment which further underline the role of a parent for continuing the work with a child. The goal of this practice is to develop a maximum of childs biological potential in the critical period of its development and to enable the child to be as an independent as it can.

### P348 *Ka Ulu Lāʻau.* Hawaiian plants in public landscaping: Amending statute to increase traditional health resources for community benefit

#### Deja Ostrowski, Kealoha Fox

##### Office of Hawaiian Affairs, Honolulu, Hawaii, HI, United States

###### **Correspondence:** Kealoha Fox (kealohaf@oha.org)

Tragically, Hawai‘i is the extinction capital of the world, as the last place where many endangered species exist, over 270 of which are plants. Hawaiian plants are integral to traditional medicinal treatments. Research suggests improved use of Hawaiian plants in landscaping will prove essential to increase access for beneficial uses and normalization. By amending the Hawai‘i Public Procurement Code (HPPC) the State Legislature was provided the opportunity to support and celebrate Hawai‘i’s cultural and ecological heritage. Systems advocacy was focused to bring research theory to policy implementation utilizing the social determinates of health framework. Through multilateral legislative methods we authored, introduced and passed process amendments to the HPPC to phase in the mandatory use of Hawaiian plants in publicly-funded landscaping projects. To help with compliance, we targeted the use of phasing requirements for policy enforcement whereby 2019 (10%)/2025 (25%)/2030 (35%) Hawaiian plants shall constitute a combined minimum of the total plant footprint. Working with industry professionals, we designed a compliance form and design mapping tool. Post-hoc qualitative analysis of support testimony indicates highly favorable approval by public beneficiaries (N = 333) for this initiative. The Hawai’i Governor signed the bill HB206 CD1 into law as Act 233 in 2015, amending HPPC HRS § 103D-408, mandating that public landscaping projects incorporate “Hawaiian” plants “wherever and whenever feasible,” and providing minimum guidelines for the use of Hawaiian plants in new and renovated landscaping projects. Effective as of June 30, 2016. This statute will play an important role in advancing health and well-being of the Native Hawaiian community by: enhancing the market for locally grown products; reducing the risk of imported pests and diseases; and promoting the sense of place and quality of life we desire and expect in our homeland. Most importantly, if further ensures public tax monies used for procurement support and fund creating healthy Hawaiian ecological environments. Hawaiian plants are integral to Hawai‘i’s cultural and ecological heritage, and therefore the health of Native Hawaiian people. The use of Hawaiian plants in landscaping promotes a Hawaiian sense of place, cultural preservation, biodiversity, biosecurity, and ecosystem management. Act 233 requires the use of Hawaiian plants in public landscaping projects. They are critical to implement traditional Hawaiian medicinal practices that use native plants in their remedies such as lāʻau lapaʻau (Hawaiian herbal medicine). Increasing the use of Hawaiian plants as cultural and natural resources foster Hawaiian treatment methods within the traditional Hawaiian health system.

### P349 Trends in the use of Complementary and Alternative Medicine in the Czech Republic

#### Jitka Pokladnikova, Iva Selke-Krulichova

##### Charles University, Hradec Kralove, 50005, Czech Republic

###### **Correspondence:** Jitka Pokladnikova (jitka.pokladnikova@faf.cuni.cz)

**Question**

The prevalence rates of complementary and alternative medicine (CAM) use are on the rise in all investigated European countries. Our aim was to compare trends in CAM use among a representative sample of the general population in the Czech Republic for 2011 and 2014.

**Methods**

A cross-sectional survey was conducted. A sex-, age- and region-stratified sample of citizens aged 15 and older were randomly selected from the 2014 voter registration lists (*n* = 8,395,132). A comparative analysis of data collected in 2011 and 2014 was performed.

**Results**

Overall, 76.0% (*N* = 1365) vs. 87.0% (*N* = 1565) of the respondents reported use of one or more CAM modalities during the past 30 days in 2011 and 2014, respectively (*p* < 0.001). In both years, the top five CAM modalities used were vitamins/minerals, herbal remedies, massage, dietary supplements excluding vitamins/minerals, and relaxation. Nevertheless, only herbal teas (48.0% vs. 53.0%, *p* = 0.002), massage (20.0% vs. 26.0%, *p* < 0.001), and relaxation (10.0% vs. 19.0%, *p* < 0.001) showed a positive trend in use. On the other hand, there was a decrease in dietary supplements use (excluding vitamins/minerals) (9.0% vs. 3.0%, *p* = 0.007), with vitamins/minerals use remaining unchanged (55.0% vs. 56.0%, *p* > 0.05).

**Conclusions**

The prevalence of CAM use in the Czech Republic is increasing, especially in the areas of biologically, body, and mind based CAM therapies. There is a growing need to educate health care professionals about the efficacy and safety of CAM to match the patients' demand for CAM.

### P350 Daily symptom obtainment technology and processes in Korean Medicine Personal Health Record Platform

#### Jinsoon Seo, Hyunchul Jang

##### Korea Institute of Oriental Medicine, Daejeon, South Korea

###### **Correspondence:** Hyunchul Jang (hcjang@kiom.re.kr)

**Purpose**

Symptoms are used as an important basis for diagnosis in Korean medicine. However, in the process of diagnosis, it is not easy to accurately identify the symptoms of daily life. Also, patients often fail to answer correctly because they rely on uncertain memories. Therefore, recording the symptoms of daily life can be helpful for diagnosis and prognosis. The purpose of this study is to derive the daily symptom obtainment techniques and processes in Korean medicine personal health record platform that records symptoms easily and conveniently, records and manages its own health information, and owns health information.

**Methods**

In order to provide personalized health care services based on Korean medicine, significant symptom collection items were derived. Significant symptom means a symptom which is useful for the patient to record among the symptoms used for the diagnosis of the Korean medicine. To do this, we examined the classical literature and previous research analysis items of Korean medicine, and then selected symptom items to be collected considering the ease of symptom recording.

**Results**

The selected significant symptoms are divided into original symptoms (素證) and daily symptoms. The original symptoms are usual physiological symptoms. The original symptom items were selected such as complexion, cold (warm) hands and feet, urine color, feces pattern, chills, amount of water to drink, urinary frequency, digestive status, frequent symptom groups, and etc. Daily symptoms are symptoms that are perceived in everyday life. They are divided into daily emotion, stool/urine/sleep, and subjective symptoms. Because of the nature of the symptoms, it is difficult to collect them automatically. Therefore, the survey type, daily emotion, human body, symptom classification, and natural language collection technique are derived as a method for user to collect symptoms. The user can enter the region and intensity on the human body for symptoms such as pain, pulling, and itching in the skin.

**Conclusion**

In this study, we selected significant symptoms that are meaningful when recorded by users in the symptoms required for diagnosis in Korean medicine. The symptoms are used as individual health care indexes through classification and scoring. We have configured the Korean Medicine Personal Health Record (KM PHR) platform to record significant symptoms. And we developed it to record the symptoms with a simple operation on the platform to increase the rate of symptom obtainment. Patients can share their symptoms with their doctor and can be connected to a diagnosis. Doctors can refer to shared symptoms to help with diagnosis and prognosis. In the future, we plan to use PHR health records as health information for EMR, EHR, CDSS and so on.

### P351 Organic food consumption during pregnancy and consumer profiles, food patterns and intake: The KOALA birth cohort study

#### Ana P Simões-Wüst^1,2^, Carolina Moltó-Puigmartí^3^, Martien van Dongen^3,4^, Pieter Dagnelie^3,4^, Carel Thijs^3^

##### ^1^ Obstetrics, University Hospital Zurich, Zurich, Switzerland; ^*2*^*Research, Clinic Arlesheim, Arlesheim, Switzerland;*^*3*^*Epidemiology,CAPHRI School for Public Health and Primary Care, Maastricht University, Maastricht, Netherlands;*^*4*^*CARIM School for Cardiovascular Disease, Maastricht University, Maastricht, Netherlands*

###### **Correspondence:** Ana P Simões-Wüst (anapaula.simoes-wuest@usz.ch)

Possible associations between consumption of organic foods and demographic characteristics, lifestyle, dietary patterns and macro- and micronutrient intakes during pregnancy have been investigated.

Women participating in the Dutch KOALA Study filled in questionnaires on lifestyle and food frequency (*n* = 2786). Based on the origin of various food groups, organic participant groups were defined, which were then compared with the group of participants consuming conventional food only (reference). Consumers of organic food exhibit sociodemographic characteristics and lifestyle characteristics that differ from people consuming conventional foods: e.g. more often adhered to specific living rules, such as vegetarianism or anthroposophy. Consumption of organic food was associated with food patterns comprising more products of vegetable origin – soy/vegetarian products, vegetables, cereal and cereal products, bread, fruits, legumes – and fewer animal products – milk and milk products, meat and meat products – than conventional diets. In addition, the consumption of sugar, sweets and sweet sauces as well as of potatoes was lower among the participants with an at least partially organic diet. These differences reflected those in the intake of macro- and micronutrients, including vitamin D and B12 intake that was only partially compensated by vitamin supplementation. Organic food users showed differences in the origin of proteins, iron and *trans* fatty acids compared with conventional food users.

Our results show that a wide variety of characteristics – including specific dietary patterns and food intake – are associated with the consumption of organic food. These aspects should be taken into consideration when studying possible effects of organic food consumption on health-related characteristics.

### P352 The practice of transcendental meditation as self-care technique in small town

#### Eva Tihanyi^1^, Gabriella Hegyi^2^

##### ^1^ Faculty of Health Sciences, University of Pecs, Pecs, Hungary; ^*2*^*Confucius Institute of TCM, University of Pecs, Pecs, Hungary*

###### **Correspondence:** Eva Tihanyi (evatihanyi2010@gmail.com)

The study examines a complex, naturopathic health program. The patients who regularly visited phytoterapist for herbal medicine, vitamins, minerals, herbs had the opportunity to participate in Transcendental Meditation (TM) courses. The aim was to offer self-care technique to improve physical health. TM is seemed to be new and unique for the patients. As we offered it reduces stress and supports healing process. Motivation and attitudes of practicing TM for self-healing were measured in a period of twelve months. The survey was self-reporting, used anonym questionnaires of TM practices with opened and multiple choices. The population was relatively small, but covered everybody who was trained by TM exercise. The results were processed on a statistical method. The results showed that the patients were not really motivated to practice TM. Comparing to their continuous consumption of vitamins probable it was the only belief to heal psychical health. The place of the survey was in a small town where people are unfamiliar with meditation practices. Most of the people refer the lack of time if they practice TM more often it would influence positively their health. Nonetheless, everybody would offer TM as stress-management technique to others. The strong personality of the TM trainer was determinative. It needs further researches, how people could be interested promoting their health with TM. The conclusion is that patients believe more in biologically based herbal products than in meditation, which they could do themselves.

Keywords: meditation, stress-management, motivation, self-healing

### P353 Considerations on multiple testing procedures in randomized controlled trials of Chinese Herbal Medicine

#### Ying Zhang, Xun Li, Yutong Fei, Jianping Liu

##### Center for Evidence Based Chinese Medicine, Beijing University of Chinese Medicine, Beijing, China

###### **Correspondence:** Ying Zhang (novelzhang@sina.com)

**Question**

Chinese Herbal Medicine (CHM) is an increasing popular remedy, and more and more randomized controlled trials (RCTs) are being carried out to confirm the efficacy and safety of CHM. Due to the complex formula, prescriptions of CHM usually contain more than one single herbs, which may affects different Zang, and then, improve several symptoms which related to the disease. Many investigators hope to set more than one endpoint in a RCT. Three or more arms may be necessary for dose exploring. Multiple time points often were planned to long duration trials. All of above scenarios should consider the problem of multiple testing.

**Methods**

Our simulations and discussions were based on a RCT with total of 120 diabetes patients. They were randomly allocated to receive either Gegen Qinlian Decoction (GQD) or placebo for 12 weeks. Study outcomes include HbA1c, FPG, 2hPG, blood lipids, HOMA insulin resistance (HOMA-IR) and β cell function (HOMA-β).

**Results**

Various approaches for correcting the alpha error were used, including Bonferroni, Tukey, Hochberg and Holm's step-down methods. Bonferroni was too far conservative. With the increasing of post hoc testing, the false significant results were more likely.

**Conclusions**

Limiting comparisons between groups is necessary to control false discovery rate. In addition, to identify a single primary endpoint or use a composite endpoint is an acceptable alternative to multiple endpoints.

### P354 The role of machine learning in the diagnosis of diabetic nephropathy and prescriptions of Chinese Herbal Medicine

#### Ying Zhang^1^, Jianping Liu^1^, Xiaolin Tong^2^

##### ^1^ Center for Evidence Based Chinese Medicine, Beijing University of Chinese Medicine, Beijing, China; ^*2*^*China Academy of Chinese Medical Sciences, Guang’anmen Hospital, Beijing, China*

###### **Correspondence:** Jianping Liu (jianping_l@hotmail.com)

**Question**

Diabetic nephropathy (DN) is the leading cause of end-stage kidney disease. The pathogenesis of DN is diverse and yet to be fully clarified. Chinse Herbal Medicine (CHM) has particular advantages to reach well prognostic outcomes in blood pressure, glucose and lipid level. Despite the popularity of CHM in China, the diagnosis and prescriptions are still complex, and will depend on the specific Zheng, laboratory examinations, physical profile and patient self-reported outcomes etc. Our study aimed to construct a classifier based on machine learning method to optimize the procedure of diagnosis and prescription.

**Methods**

A consecutive data recording was carried out on a phase III DN case from 2010 till now. This case was male, aged 45. He used simplex TCM for more than six years, and completed 60 return visits. The creatinine (Cr) was 132 umol/L in 2010. At the last visit in 2016, the CR was 122.7 umol/L. No disease progress happened in the six years. Many symptoms were improved.

**Results**

Several machining learning methods were used to classify the Zheng of TCM and formula. For each visits, the collected information included height, weight, BMI, blood pressure, HbA1c, 24 h urine protein, 24 h urine volumn, GLU, BUN, CR, ALT, AST, TBIL, DBIL, UA, CHO, TG, LDL, HDL etc. Patients self-reported symptoms, pulse and tongue picture were also captured. More than 20 herbal medicines were combined as a prescription in different visits.

**Conclusions**

Machining learning covers a various techniques including Bayesian classification, decision trees, regression and neural network etc. It is very helpful to explore the different stages for a certain disease and prescribe the targeted TCM formula.

**Publisher’s Note**

Springer Nature remains neutral with regard to jurisdictional claims in published maps and institutional affiliations.

